# Short tandem repeats in populations of the Qinghai-Tibet Plateau and adjacent regions provide insights into high-altitude adaptation

**DOI:** 10.1126/sciadv.adx1590

**Published:** 2025-10-17

**Authors:** Yuguo Huang, Mengge Wang, Zhiyong Wang, Xiaojun Liu, Yuhang Feng, Jie Zhong, Hailin Huang, Jia Geng, Ting Tang, Chao Liu, Yu Lu, Jing Cheng, Fengxiao Bu, Guanglin He, Huijun Yuan

**Affiliations:** ^1^ Institute of Rare Diseases, West China Hospital of Sichuan University, Sichuan University, Chengdu, China.; ^2^ Center for Archaeological Science, Sichuan University, Chengdu, China.; ^3^ Department of Forensic Medicine, College of Basic Medicine, Chongqing Medical University, Chongqing, China.; ^4^ Anti-Drug Technology Center of Guangdong Province, Guangzhou, China.; ^5^ Shanghai Key Laboratory of Gene Editing and Cell Therapy for Rare Diseases, Fudan University, Shanghai, China.

## Abstract

Short tandem repeats (STRs) confer evolutionary advantages across various species, yet their roles in human high-altitude adaptation remain largely unexplored. In this study, we analyzed over 1.1 million STRs in 7876 whole-genome sequencing samples, including 3808 newly sequenced Tibeto-Burman (TB) speakers and Han Chinese individuals inhabiting the Qinghai-Tibet Plateau and neighboring regions. We characterized more than 570,000 polymorphic STRs across coding/noncoding genomic and functional contexts, revealing distinct STR variation patterns specific to TB speakers. Notably, divergent STRs in TB speakers were predominantly located in high-altitude adaptive genes and frequently associated with gene expression levels. Additionally, we identified over 17,000 STRs strongly associated with environmental conditions on the Qinghai-Tibet Plateau. These STRs exerted multilevel gene regulation through modulating protein coding, fine-tuning cis-regulatory elements, and modulating biological pathways. Furthermore, we observed substantial contributions of functional and adaptive STRs to human phenotypic diversity. Collectively, our findings provide genomic STR resources of TB speakers and genetic insights into their roles in human adaptive evolution.

## Teaser

Analyzing 7876 genomes reveals that short tandem repeats contribute to high-altitude adaptation via multilevel gene regulation.

## INTRODUCTION

More than half of the human genome consists of repetitive sequences (*1*), and unraveling the variation patterns and functional roles of this genetic “dark matter” has emerged as a central focus in genomic research. Short tandem repeats (STRs), characterized by consecutive motifs ranging from 1 to 6 bp, are ubiquitously distributed across genomes throughout the tree of life (*2*). Because of their repetitive structure, STRs exhibit higher mutation rates compared to other genomic elements, thereby substantially contributing to genomic variability and diversity (*3*). Historically, the roles of STRs in shaping human genomic and phenotypic characteristics have been rarely explored because of technical challenges in accurately profiling these repetitive regions. Recent advances in whole-genome sequencing (WGS) technologies and bioinformatics methodologies largely addressed these limitations, enabling more accurate genotyping of genome-wide STRs (*4*). On the other hand, large-scale population and medical cohorts, such as the UK Biobank (*5*), launched in recent years, provided invaluable resources for investigating the variation patterns and biological functions of STRs across global populations. Existing genome-wide association studies (GWASs) and phenome-wide association studies (PheWASs) focusing on STRs have uncovered widespread associations between STRs and human traits, including body height, blood and serum traits, organ functions, and psychiatric outcomes (*6*–*8*). Moreover, STR variations are also implicated in polygenic human disorders such as Parkinson’s disease (*9*), Alzheimer’s disease (*10*), and autism spectrum disorder (*11*). These findings highlight the critical roles of STRs in influencing human complex traits and diseases, contributing to a better understanding of the missing heritability inherent in current genetic studies (*3*).

Emerging evidence indicates that polymorphic STRs (pSTRs) can influence genomic structures and functions through diverse mechanisms, such as modulating the spatial proximity of regulatory elements, splicing efficiency, alternative splicing, methylation patterns, and chromatin states (*12*), which have profoundly driven the evolution of species. For instance, ~8 to 9% of vertebrate and plant proteins contain homopolymer tracts encoded by STRs (*13*). In mammals, the *Runx2* gene features a variable polyglutamine/polyalanine repeat domain that regulates the transactivation potential of local DNA sequences, thus functioning as an evolutionary “tuning knob” to accelerate morphological trait evolution in mammals (*14*). Similarly, in the *Arabidopsis thaliana* genome, length variations of STRs encoding glutamine and asparagine amino acid repeats can alter protein functions and enhance adaptation to local bioclimatic conditions (*15*). These findings collectively underscore the essential roles of STRs in maintaining genomic functionality and promoting biodiversity.

Adapting to and thriving in highland regions represent a milestone in the history of human evolution. Both genetic and archaeological data support the Paleolithic occupation and late Neolithic expansion of human beings on the Qinghai-Tibet Plateau (*16*, *17*). The extreme environmental conditions, including low oxygen levels, low temperature, and high ultraviolet radiation, have largely influenced the genetic architecture and phenotypic diversity of these populations. For instance, high-altitude Tibetans exhibit well-documented adaptive traits such as reduced hematopoiesis, enhanced lung function, and improved reproductive fitness compared to lowland Han Chinese (*18*, *19*). Deep screening of genomic variations in populations centered on the Qinghai-Tibet Plateau has revealed the genetic landscape underlying these adaptive phenotypes. Notably, haplotypes of *EGLN1* and *EPAS1*, which are involved in the hypoxia-inducible factor (HIF) pathway, have been identified as selectively advantageous genetic variants in Tibetans that contribute to their relatively lower hemoglobin levels and better ventilation (*20*–*23*). Recently, Zheng *et al.* (*24*) conducted WGS on 1001 indigenous Tibetans and identified 4320 short variants and 192 genes under natural selection, further supporting the polygenic and pleiotropic nature of high-altitude adaptation. In parallel, increasing evidence highlights the widespread effects of structural variation (SV) rewiring in high-altitude adaptive phenotypes (*25*–*27*). For example, Ouzhuluobu *et al.* (*25*) conducted de novo assembly of a Tibetan genome and identified a 163-bp intronic deletion in the *MKL1* gene. This variant shows marked divergence between highland Tibetans and lowland Han Chinese and is associated with lower systolic pulmonary arterial pressure in Tibetans. Another example is the Tibetan-specific deletion identified by Shi *et al.* (*27*), which disrupts a superenhancer and down-regulates *EPAS1* through both proximal and distal interactions. These findings indicate that diverse genetic variants and mechanisms have contributed to high-altitude adaptation, and further functional characterization of these variants can enhance our understanding of the genetic basis of human adaptive evolution. However, despite their biological importance, the variation patterns of STRs in highland populations and their roles in influencing high-altitude adaptation remain largely uncharacterized.

Tibeto-Burman (TB) speakers constitute a culturally cohesive group predominantly inhabiting the Pan-Tibetan Highlands and adjacent regions in East and Southeast Asia, where geographical barriers and harsh environmental conditions have profoundly shaped their genetic backgrounds and phenotypic characteristics (*28*). Linguistic and genetic studies suggest a shared origin of TB- and Sinitic-speaking populations (e.g., Han Chinese) in the Yellow River basin of northern China (*29*, *30*). Consequently, the distinct demographic history and evolutionary trajectory of these populations since their divergence from Sinitic speakers make them ideal for investigating the genetic basis of local adaptation. Recently, Shi *et al.* (*31*) mapped STR variations in 3983 lowland Chinese individuals in the NyuWa Genome Resource, revealing extensive population-specific and functional variability of STRs. However, genome-wide STR variations in the geographically and genetically distinct TB-speaking populations are underexplored.

In this study, we systematically characterized the variation of over 1.1 million STRs across 7876 WGS samples, including 3808 newly sequenced TB speakers and Han Chinese individuals residing on the Qinghai-Tibet Plateau and in neighboring regions at elevations up to ~4500 m above sea level. We identified more than 570,000 pSTRs and elucidated their genomic contexts and functional semantics in gene regulation. Our results revealed distinct STR variation patterns in TB-speaking populations, including genomic variability, ancestry admixture, population divergence, and allelic expansion. By integrating genetic and geographical data, we demonstrated the associations between STR variations and the harsh environmental conditions of high-altitude regions, highlighting their regulatory potential in shaping high-altitude adaptation in TB-speaking populations. Furthermore, we explored the contribution of functional and adaptive STRs (aSTRs) to human phenotypic variation. Our findings delineate the distinctive STR variation spectrum across multiple highland and lowland populations, providing invaluable insights into the genetic mechanisms underlying high-altitude adaptation.

## RESULTS

### Genome-wide STR genotyping in high-coverage genomes

**Fig. 1.**
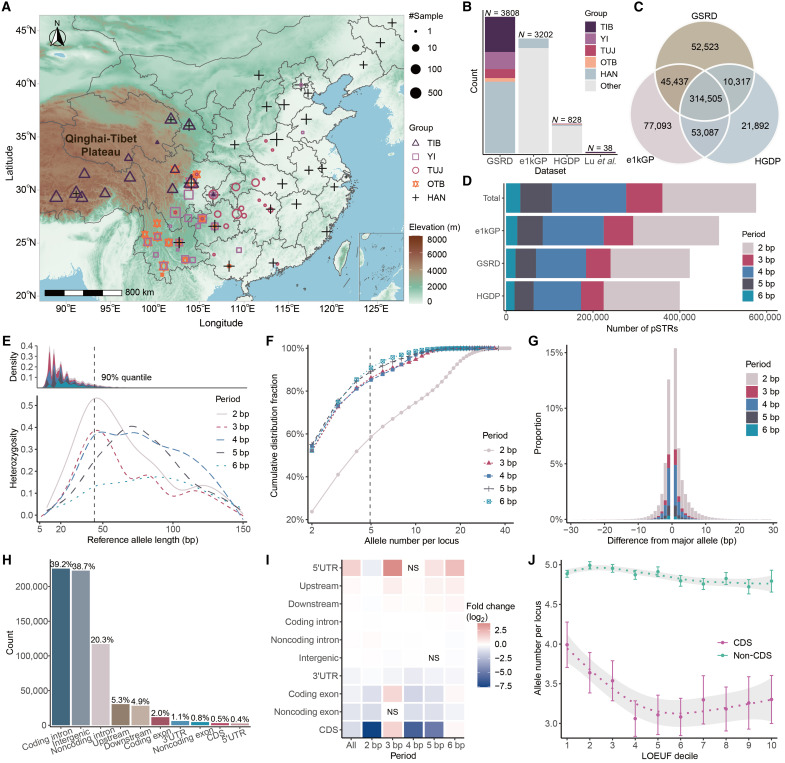
Discovery of STR variations. (**A**) Geographical distribution of 3808 newly sequenced TB-speaking and Han Chinese samples in the GSRD project. (**B**) Number of samples included in different WGS projects analyzed in this study. (**C**) Overlaps of pSTRs among different WGS projects. (**D**) Cumulative number of pSTRs found across WGS projects. (**E**) Correlation between heterozygosity and reference allele length for pSTRs. (**F**) Cumulative fraction of allele numbers across different pSTR loci. The dashed line indicates the mean number of alleles across all loci. (**G**) Distribution of allele repeat differences relative to major alleles of pSTRs. (**H** and **I**) Constitution (H) and enrichment (I) of pSTRs in different genomic regions. Unlabeled tiles indicate significant enrichment or depletion (2000 permutations, Benjamini-Hochberg–adjusted *P* < 0.05), while NS denotes nonsignificant results. (**J**) Mean allele number per locus for pSTRs in CDS and non-CDS regions of genes across different LOEUF deciles. Error bars denote 95% confidence intervals.

Genome-wide STR variations were jointly analyzed in 1811 TB-speaking and 1997 Han Chinese (referred to as HAN) samples in the Genome Sequencing of Rare Disease - 100K^West China Hospital^ project (hereafter referred to as GSRD). The TB-speaking individuals were primarily located on the Qinghai-Tibet Plateau in the adjacent regions of southwestern China, whereas the Han Chinese samples were broadly distributed across central and eastern China (Fig. 1A). In addition, 3202 samples from the expanded 1000 Genomes Project (e1kGP) (*32*), 828 samples from the Human Genome Diversity Project (HGDP) (*33*), and 33 Tibetan and 5 Sherpa samples previously reported by Lu *et al.* (*34*) were included as reference populations (Fig. 1B and data S1). Principal components analysis (PCA) based on genome-wide biallelic single-nucleotide polymorphism (SNPs) revealed that the newly reported GSRD samples clustered closely with East Asian populations (fig. S1A). Major TB-speaking groups, including Tibetan (TIB), Yi (YI), and Tujia (TUJ) people, formed distinct clusters, indicating their specific ancestral backgrounds (fig. S1B). In contrast, other TB speakers (referred to as OTB) displayed more heterogeneous genetic patterns but still shared strong affinities with the three major groups (fig. S1C). These results confirm the representativeness of our sampling strategy.

We applied stringent quality control to the WGS data to accurately call STR genotypes. After filtering, the GSRD samples achieved a mean sequencing coverage of 42.0 ± 5.2× (SD), with multiple quality metrics meeting the filtering thresholds (fig. S2). We implemented the EnsembleTR (*35*) pipeline, which leverages the strengths of two widely used tools, namely, HipSTR (*36*) and GangSTR (*37*), to call STRs from these WGS data. The performance of this pipeline on our data was validated using orthogonal approaches (text S1). First, we observed 97.6% concordance between EnsembleTR and capillary electrophoresis calls for 390 Marshfield STR loci in the Human Genome Diversity Project-Centre d′Etude du Polymorphisme Humain (HGDP-CEPH) cell line panel (*38*) (fig. S3). Second, the EnsembleTR pipeline achieved ~99.8% Mendelian inheritance (MI) consistency and 99.6% monozygotic genotype concordance among the Chinese Quartet reference DNA samples (*39*) (fig. S4). Third, compared to STR calls derived from PacBio HiFi long reads, the EnsembleTR pipeline demonstrated 94.7% accuracy (fig. S5). These results support the reliability of the WGS-based STR–genotyping pipeline adopted in this study.

We applied this pipeline to call 1,130,989 autosomal STR loci in 7876 WGS samples. Following call-level filtering (fig. S6), 1,069,996 high-quality STRs were retained for downstream analysis, with a mean call rate of 95.2 ± 1.6% (SD) per sample and 97.2 ± 8.0% (SD) per locus. We conducted multiple assessments to further evaluate the quality of the STR dataset. First, allele transmission concordance was assessed in 576 GSRD parent-child trios. In these family samples, the MI consistency rate reached 99.3 ± 0.08% (SD), closely matching the 99.4 ± 0.08% (SD) observed in 602 trios from the e1kGP project (fig. S7A). Meanwhile, consistent MI patterns were observed across motif and allele length distributions in both datasets (fig. S7, B to D). Second, major allele frequencies of pSTRs showed a strong correlation between Han Chinese samples in the GSRD project and those from the e1kGP and HGDP projects (Pearson’s *r* = 0.983, *P* < 2.2 × 10^−16^; fig. S8A). This correlation was also evident when comparing GSRD samples with East Asian samples from the e1kGP and HGDP projects (fig. S8B). Third, a robust correlation was observed between the lengths of major alleles and reference alleles up to 150 bp (Pearson’s *r* = 0.979, *P* < 2.2 × 10^−16^; fig. S8C). These results confirm the accuracy and reliability of the genome-wide STR genotypes.

### Landscape of STR variations

In the final STR call set, 575,433 (53.8%) STRs were polymorphic (i.e., pSTRs), each exhibiting at least two distinct alleles, while the remaining 494,563 (46.2%) loci were monomorphic STRs (mSTRs) (data S2). Specifically, 422,782, 490,122, and 399,801 pSTRs were identified from GSRD, e1kGP, and HGDP samples, respectively. Most pSTRs were shared across different WGS projects, whereas a greater number of distinct pSTRs were observed in the e1kGP project (Fig. 1C), likely due to its relatively larger sample size and broad population coverage. Notably, 52,523 pSTRs were exclusively identified in GSRD samples, representing a 1.4-fold increase compared to the number of distinct pSTRs identified in HGDP samples. When restricted to East Asian samples, 150,259 pSTRs were exclusively detected in GSRD samples (fig. S9A). While most pSTRs were common across East Asian populations, 43,728 of these pSTRs were specifically observed in TB-speaking populations (figs. S9B and S10). These findings highlight the substantial contribution of the GSRD project in capturing the extensive genomic diversity of East Asians, particularly among TB speakers who were underrepresented in previous studies.

The most abundant pSTRs were di- and tetranucleotide repeats (Fig. 1D), whereas tetra- and pentanucleotide repeats were more common in mSTRs (fig. S11, A and B). Consistent with their greater genetic variability, pSTRs generally contained more repeat units and exhibited longer alleles than mSTRs (fig. S11, C and D). Most of pSTRs were short with reference alleles of <50 bp (Fig. 1E). In addition, 174,744 pSTRs (30.4%) were classified as hypervariable with a heterozygosity of >0.1, and pSTRs with shorter motifs and longer alleles exhibited higher polymorphisms (Fig. 1E and fig. S12, A to C). Furthermore, over half of the pSTRs had more than five distinct alleles, highlighting their multiallelic nature (Fig. 1F). On average, 4.93 ± 4.93 (SD) alleles were observed per locus, ranging from 3.16 ± 1.93 (SD) for hexanucleotide STRs to 7.43 ± 6.63 (SD) for dinucleotide STRs. We further investigated the mutation patterns of pSTR alleles and found a higher proportion of expansions (52.4%) than contractions (47.6%), with allele frequencies decreasing monotonically as they deviated further from the major alleles (Fig. 1G). Most pSTR alleles were rare [allele frequency (AF) < 0.001, 36.9%] or low frequency (AF < 0.01, 18.0%), and only 33.1% alleles were common variants with an AF of >0.05. In line with previous observations (*31*), only 9.7% of pSTRs had a major AF of <0.5 (fig. S12D). We specifically analyzed dimorphic pSTRs and observed that 33.6% of trinucleotide pSTRs were singletons (fig. S12E), significantly higher than those with other motif lengths (chi-square test, χ^2^ = 79.9, *P* < 2.2 × 10^−16^). Similarly, frequencies of trinucleotide alleles with allele count (AC) = 1 or AC = 2 were also increased, suggesting stronger mutational constraints at these loci (fig. S12F).

To extrapolate the genomic contexts of these STRs, we evaluated their distribution across different genomic regions. We found that most STR loci were located in intergenic and intronic regions, and only 2991 (0.5%) pSTRs and 4130 (0.8%) mSTRs were in coding sequence (CDS) regions (Fig. 1H and fig. S13A). In addition, a considerable proportion of pSTRs resided in potential regulatory regions, including upstream (5.3%), downstream (4.9%), and untranslated regions (UTRs; 1.5%) of genes. As expected, tri- and hexanucleotide STRs were predominantly found in CDS and 5′UTR regions (fig. S13, B and C). These STRs were more frequently polymorphic, likely reflecting the reduced impact of triplet repeats on the integrity of open reading frames. Permutation enrichment analysis revealed that pSTRs were more enriched in 5′UTR and upstream genic regions but depleted in regions such as 3′UTR and CDS compared to mSTRs (Fig. 1I and fig. S13D). Consistent with previous findings (*31*), di-, tetra-, and pentanucleotide pSTRs were significantly depleted in CDS regions, whereas tri- and hexanucleotide pSTRs were significantly enriched in CDS and 5′UTR regions (2000 permutations, Benjamini-Hochberg–adjusted *P* < 0.05).

Notably, CDS pSTRs exhibited lower heterozygosity and sequence entropy (fig. S14, A and B), indicating stronger genomic constraints acting on these variants. We took a further step to score the tolerance of genes to inactivation using the loss-of-function (LoF) observed/expected upper-bound fraction (LOEUF) (*40*). Genes with lower LOEUF values are more intolerant to LoF mutations and likely have key biological functions. We found that genes containing CDS pSTRs exhibited higher LOEUF scores compared to those without these variants (fig. S14C). In addition, CDS pSTRs showed reduced variability than non-CDS pSTRs, as indicated by their lower allele numbers and polymorphisms (Fig. 1J and fig. S14D). These results confirm the presence of stronger mutational constraints on coding region variants. Nonetheless, we observed an apparent increase in allele numbers for CDS pSTRs located in genes within the first three LOEUF deciles (Fig. 1J). These genes were significantly enriched in fundamental biological processes, such as neuronal system development (fig. S15). Therefore, these multiallelic CDS pSTRs might contribute to the functionality of biologically essential genes.

On the other hand, CDS variations can disrupt open reading frames or alter transcript splicing, which causes LoF of genes. To assess the occurrence of LoF variants in our dataset, we used Ensembl Variant Effect Predictor (VEP) (*41*) to predict the functional consequences of STR variations. Variants annotated as “frameshift”, “splice-acceptor”, “splice-donor”, “start-lost”, or “stop-gained” were classified as predicted LoF (pLoF) variants. In total, we identified 2825 pLoF variants across 1043 pSTR loci, affecting 919 genes (data S3). Enrichment analysis revealed that these affected genes were enriched in key pathways, e.g., those related to organismal development and cell signaling, suggesting their potentially important biological roles (data S3). Most of pLoF variants were classified as frameshift (33.3%), splice-donor (32.5%), and splice-acceptor (24.7%) variants, while only 6.6 and 2.9% were categorized as start-lost and stop-gained variants, respectively (fig. S16A). Among these, dinucleotide alleles were more frequently predicted as splice-donor variants, whereas most tetra- and pentanucleotide alleles were classified as splice-acceptor variants (fig. S16B). In contrast, frameshift, start-lost, and stop-gained variants were predominantly observed among tri- and hexanucleotide pSTRs (fig. S16B).

Compared to neutral variants, pLoF variants are often subjected to stronger selection pressures, resulting in their relatively lower frequencies in the general population. We found that pSTRs harboring pLoF variants exhibited lower heterozygosity and sequence entropy, which was indicative of purifying selection acting on these variants (fig. S16, C and D). We further evaluated the pLoF burden across individual genomes (fig. S17A). As a result, the highest number of frameshift variants was observed in Tibetans (95% confidence interval, 16.9 to 17.2). In addition, although rare, more stop-gained variants were also detected in Tibetans than in other non-Tibetan individuals, except for Central and South Asians. Furthermore, 76 pLoF variants affecting 66 genes were exclusively characterized among Tibetans (fig. S17B). We assessed genomic constraints on these pLoF genes using multiple metrics, including LOEUF, pLI [the probability of being LoF intolerant (*42*)], pHaplo [the probability of haploinsufficiency (*43*)], and pTriplo [the probability of triplosensitivity (*43*)]. We found that Tibetan-specific pLoF genes were under greater genomic constraints and more intolerant to LoF mutations, as evidenced by the relatively lower LOEUF (two-sided Wilcoxon test, *P* = 0.002) and higher pLI (two-sided Wilcoxon test, *P* = 0.095), pHaplo (two-sided Wilcoxon test, *P* = 0.160), and pTriplo (two-sided Wilcoxon test, *P* = 0.280) compared to other pLoF genes (fig. S17C), suggesting their high genomic conservation and important biological functions.

### Genetic structure and admixture of TB-speaking populations

**Fig. 2.**
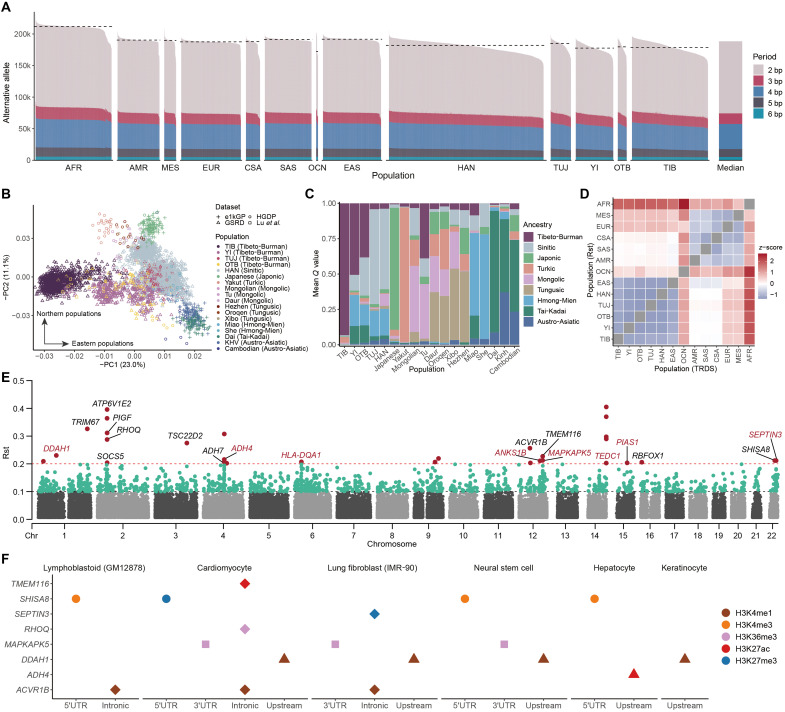
STR variation patterns in TB-speaking populations. (**A**) Mean number of alternative alleles per genome across different population groups. Horizontal dashed lines indicate the median values. (**B**) Genetic structure of East Asian populations revealed by PCA analysis based on hypervariable pSTRs. (**C**) Admixture patterns of ancestry components (*Q*) among ethnolinguistically distinct East Asian populations inferred from the top 10 principal components. (**D**) Mean Rst values (upper triangle) and TRDS scores (lower triangle) between TB-speaking populations and reference population groups. (**E**) Divergence of pSTRs between Tibetans and Han Chinese measured by Rst. pSTRs located within genes are labeled with the corresponding gene name. Genes not included in the high-altitude adaptive gene set curated by Zheng *et al.* (*24*) are marked in red. (**F**) Epigenetic marks overlapping highly divergent pSTRs between Tibetans and Han Chinese (Rst_TIB-HAN_ > 0.2) in protein-coding genes across different cell lines. AFR, African; AMR, American; MES, Middle Eastern; EUR, European; CSA, Central and South Asian; SAS, South Asian; OCN, Oceanian; EAS, East Asian.

Complex demographic events and environmental pressures have shaped the distinct genomic characteristics of TB speakers, as evidenced by studies based on SNPs and SVs (*24*, *26*, *27*, *34*, *44*). In this study, we resolved the genetic structures and admixture patterns of TB speakers using genome-wide STR data. In total, we characterized 2,486,015 alternative (nonreference) alleles from global populations, with a median of 188,280 alleles per genome (Fig. 2A). Individuals from African ancestry carried the highest number of alternative alleles, while TB-speaking individuals had comparable alternative alleles to the Han Chinese and other East Asians, reflecting their relatively close genetic relationships. To further explore the genetic distinctions and connections among these populations, we conducted PCA and Uniform Manifold Approximation and Projection (UMAP) analyses on hypervariable pSTRs (heterozygosity > 0.1). The results revealed clear genetic structures among geographically and ethnolinguistically distinct populations (Fig. 2B and fig. S18A). The first and second principal components generally corresponded to the longitudinal and latitudinal dispersions of East Asian populations, with linguistically related populations more tightly clustered. Consistent with SNP-based findings (fig. S1, B and C), PCA and UMAP distinguished Tibetan and Yi individuals from Han Chinese individuals, whereas most Tujia individuals overlapped with Han Chinese individuals (Fig. 2B and fig. S18A). The Tibetan, Yi, and Tujia populations were distinguishable from each other, with other TB speakers dispersed across the three major groups (Fig. 2B and fig. S18B). These results confirmed the genetic heterogeneities among different TB-speaking populations, indicating the apparent differentiation of Tibetan and Yi populations from Han Chinese.

Further, ancestry analysis of global populations highlighted the distinct genetic backgrounds of TB speakers (fig. S19). To investigate genetic interactions and admixture patterns between TB-speaking populations and their East Asian neighbors, we quantified the admixture profiles of these populations by assigning genetic ancestry components to different language families (Fig. 2C). As a result, linguistically related populations generally shared similar ancestry profiles. Among these, Tibetans presented the highest proportion of TB-related ancestry, followed by Yi individuals. In contrast, Tujia individuals carried minimal TB-related ancestry, which was predominantly replaced by Sinitic-related ancestries. Notably, we observed extensive yet distinct admixture patterns across TB-speaking populations (Fig. 2C and fig. S20). For example, a noticeable proportion (~4%) of Turkic-related ancestries was detected in Tibetan and Sherpa samples, which is consistent with their northern demographic origins and recent population movements and admixtures (*30*, *45*). In addition, populations such as Lisu, Hani, and Lahu shared substantial ancestries with Tai-Kadai–speaking populations, whereas Hmong-Mien–related ancestries were prevalent in the Naxi and Bai people, indicating considerable gene flow among TB speakers and their indigenous neighbors.

### STR divergence between TB speakers and Han Chinese

The evolving variation of STRs can reshape the trajectories of evolutionary adaptations across species (*2*, *12*). Therefore, we supposed that highly divergent pSTRs in TB speakers may redefine the genetic footprints of local adaptations to extreme environments. We used the fixation index (Rst) and tandem repeat disparity score (TRDS) to quantify genome-wide disparities of pSTR alleles between different populations. Both metrics revealed close genetic distances among intracontinental populations, whereas intercontinental populations exhibited greater discrepancies (fig. S21, A and B). As expected, we observed strong genetic proximity among TB speakers, Han Chinese, and other East Asian populations (Fig. 2D and fig. S21, C and D). When conditioned on Han Chinese (the GSRD-HAN group), Tibetan and Yi populations consistently exhibited higher Rst and TRDS values than Tujia and other TB speakers (fig. S21, C and D). On the basis of a threshold of Rst_TBs-HAN_ > 0.1, we identified 1450 nonredundant pSTRs that were divergent in TB speakers (data S4). Notably, most TB-divergent pSTRs were private to the target populations, with 90.2% (1225 of 1450) exclusively identified in Tibetans. With a tightened threshold of Rst_TBs-HAN_ > 0.2, we characterized 30 highly divergent pSTRs between Tibetans and Han Chinese (Fig. 2E), among which 19 pSTRs resided in non-CDS regions of genes. Notably, 11 of these pSTRs were found within previously known high-altitude adaptive genes characterized by multiple studies (*24*), predominantly intronic variants (9 of 11).

Further, we observed abundant active histone modifications overlapping with these pSTRs across multiple cell lines (Fig. 2F), suggesting their potential involvement in gene regulation. For example, the chr22:41915070(AGCCGG)n locus is located in the 5′UTR of *SHISA8*, a gene linked to protective and adaptive responses after hypoxia (*46*), and overlaps with a proximal enhancer (enhP)–like signature (EH38E2166737) in the registry of candidate cis-regulatory elements (cCREs) from the Encyclopedia of DNA Elements (ENCODE) project (*47*). This locus also intersects with H3K4me3 peaks in the lymphoblastoid, neural stem cell, and hepatocyte cell lines, further supporting the enhancer activity of the flanking region (fig. S22A). Meanwhile, this STR was highly divergent between Tibetans and Han Chinese (Rst_TIB-HAN_ = 0.212; fig. S22B); thus, it may act as an active cis-regulator of the *SHISA8* gene in Tibetans. Another example is the chr12:111894767(AC)n locus, which overlaps with a distal enhancer (enhD)–like cCRE signature (EH38E1644830) and resides in the 3′UTR region of the *MAPKAPK5* gene, whose expression level responds to environmental hypoxia conditions (*48*). In addition, other genes harboring highly divergent pSTRs may also contribute to the local adaptation of TB speakers. For instance, the *DDAH1* gene encodes the subunit of dimethylarginine dimethylaminohydrolase, which was shown to promote neurogenesis and neural repair via HIF-1α under ischemia (*49*). Likewise, the *PIAS1* gene encodes the inhibitor of activated signal transducers and activators of transcription protein, a negative regulator of *HIF-1*α transcription that suppresses angiogenesis under hypoxic conditions (*50*). These findings suggest that TB-divergent pSTRs could have promoted high-altitude adaptation in TB speakers, particularly in Tibetans.

### Effect of TB-divergent STRs on gene expression

**Fig. 3.**
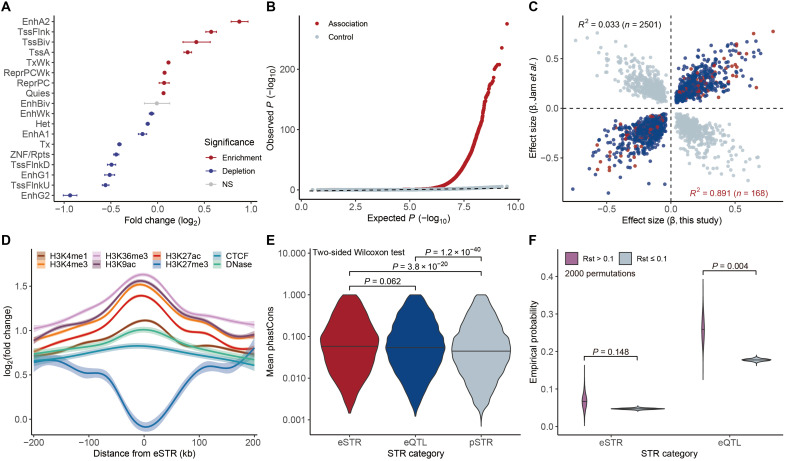
Contribution of STRs to gene expression. (**A**) Enrichment of pSTRs in chromatin states in the GM12878 cell line. Empirical *P* values were calculated on the basis of 2000 permutations and adjusted using the Benjamini-Hochberg method. Red and blue dots indicate significant enrichment and depletion (adjusted *P* < 0.05), respectively. Error bars represent 95% confidence intervals. (**B**) Quantile-quantile plot of *P* values from STR dosage and gene expression association tests. Red dots represent observed *P* values, gray dots indicate *P* values derived from permutation controls, and the black dashed line shows the null distribution of *P* values. (**C**) Correlation between effect sizes of eSTRs identified in this study and a previous study by Jam *et al.* (*35*). Blue dots denote eSTRs with concordant effects between the two studies, gray dots denote eSTRs with opposite effects, and red dots denote eSTRs identified in both studies. (**D**) Enrichment of epigenetic marks within a ±200-kb window of eSTR loci in the GM12878 cell line. CTCF, CCCTC-binding factor. (**E**) Mean phastCons scores of STRs in different categories. (**F**) Empirical probability that TB-divergent pSTRs (Rst_TBs-HAN_ > 0.1) are scored as eQTLs or eSTRs based on 2000 permutations.

Inspired by the potential impact of TB-divergent STRs on high-altitude adaptive genes, we sought to explore the comprehensive regulatory roles of genome-wide pSTRs. We examined the colocalization of pSTRs with 18 chromatin states across six cell lines from the National Institutes of Health (NIH) Roadmap Epigenomics project (*51*). We found that pSTRs were significantly enriched in active enhancers, transcription start sites, and their flanking regions, whereas they were depleted in inactive regions such as heterochromatin (2000 permutations, Benjamini-Hochberg–adjusted *P* < 0.05), highlighting their widespread regulatory potential (Fig. 3A and fig. S23). Thus, we hypothesized that STRs can mediate high-altitude adaptation by regulating the expression of implicated genes.

To verify this hypothesis, we next associated STR length variations with gene expression levels in 731 lymphoblastoid cell lines from the e1kGP project, known as the multi-ancestry analysis of gene expression (MAGE) resource (*52*). By adjusting for ancestry, sex, and other hidden covariates estimated from the expression data, we tested associations between dosages (summed repeat units of sister alleles) of hypervariable pSTRs (heterozygosity > 0.1) and the expression levels of flanking genes within a ±500-kb window using a simple linear regression model. In total, 30,532 nonredundant significant expression quantitative trait loci (eQTLs) were identified from 1,295,338 pSTR-gene pairs with Bonferroni-adjusted *P* < 0.05. By controlling the gene-level false discovery rate (FDR) at <5%, we identified 7948 nonredundant expression-associated STRs (eSTRs) for 9745 genes, including 6728 eSTRs linked to 7798 protein-coding genes (data S5).

We repeated the association test using negative controls by permuting sample identifiers. This yielded uniformly distributed *P* values that aligned with the null hypothesis, confirming the robustness of our findings by controlling for inflation of test statistics and confounding factors (Fig. 3B). Our results were further validated by comparing them to eSTRs identified by Jam *et al.* (*35*) based on samples from the Geuvadis project. We found that eSTRs found in both studies (*n* = 168) showed perfect concordance in the direction of regulatory effects, i.e., either up- or down-regulating gene expression (Fig. 3C). Compared to randomly permuted pSTR controls, we observed a strong enrichment of active epigenetic modifications in eSTR regions, such as H3K4me3 and H3K27ac, while the repressive histone mark H3K27me3 was depleted across different cell lines (Fig. 3D and fig. S24). In addition, we used the phastCons conservation score across 100 vertebrate genomes (*53*) to assess the evolutionary constraints of local sequences, where higher values indicate stronger conservation. As a result, significantly higher phastCons scores were observed for eQTL loci and STRs (two-sided Wilcoxon test, *P* < 0.05; Fig. 3E), indicating strong evolutionary constraints on these functionally relevant elements.

Building on these findings, we evaluated whether TB-divergent pSTRs also exhibited activities in regulating gene expression. Among the 1450 TB-divergent pSTRs, 376 (25.9%) were identified as eQTLs, including 98 loci classified as eSTRs. Furthermore, 121 of these loci overlapped with at least one active epigenetic mark or cCRE element (fig. S25), consistent with their potential cis-regulatory roles. To determine whether TB-divergent pSTRs were more likely to be associated with gene expression, we divided pSTRs into two groups based on the Rst threshold: those with Rst_TBs-HAN_ > 0.1 (divergent) and those with Rst_TBs-HAN_ ≤ 0.1 (nondivergent). We construct the empirical distributions with 2000 permutations (Fig. 3F) and found that TB-divergent pSTRs (Rst_TBs-HAN_ > 0.1) were more likely to be identified as eQTLs and eSTRs compared to nondivergent pSTRs (Rst_TBs-HAN_ ≤ 0.1), supporting their functional involvement in gene regulation.

For example, the chr14:105700849(AG)n locus was associated with the expression of *TMEM121* (β = 0.172, Bonferroni-adjusted *P* = 6.7 × 10^−6^; fig. S26A), which encodes the transmembrane protein 121 (TMEM121) that plays a crucial role in angiogenesis to promote oxygen and nutrient delivery under hypoxic conditions (*54*). This locus overlaps with an enhD-like cCRE signature (EH38E1747040) and exhibited the highest Tibetan-Han divergence (Rst_TIB-HAN_ = 0.405); thus, it may act as a cis-regulatory modulator in Tibetans. In addition, chr2:46518759(AC)n (Rst_TIB-HAN_ = 0.396) and chr2:46578896(AC)n (Rst_TIB-HAN_ = 0.289) were associated with the expression of high-altitude adaptive genes *ATP6V1E2*, *PIGF*, *CRIPT*, and *RHOQ*, among which the chr2:46578896(AC)n locus was also identified as an eSTR regulating both *PIGF* and *RHOQ* (fig. S26, B and C). These results provide further support for our hypothesis that genome-wide pSTRs can contribute to high-altitude adaptation through cis-regulating gene expression.

### Association of STR variations with high-altitude environments

**Fig. 4.**
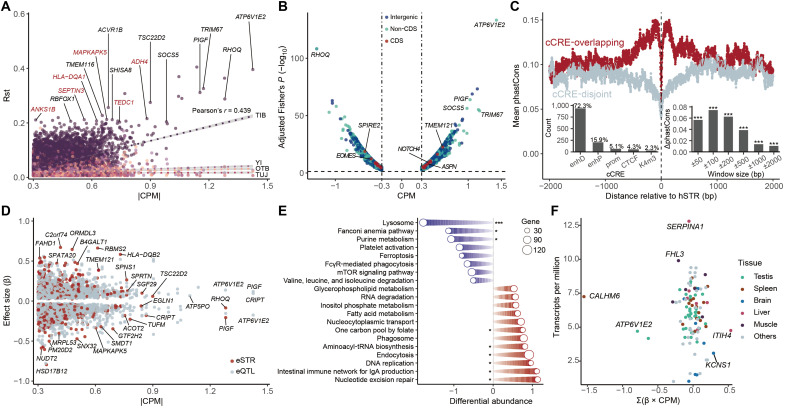
Association between STR dosages and high-altitude environments. (**A**) Correlations of |CPM| and Rst values for hSTRs between TB speakers and Han Chinese. (**B**) Effects of hSTRs on the composite environment. (**C**) Mean phastCons conservation scores in regions flanking hSTRs. The insets show the intersection of hSTRs with cCRE elements (left) and the difference in phastCons scores between cCRE-overlapping and cCRE-disjoint control hSTRs as a function of window size around the loci. Two-sided Wilcoxon test, ***P < 0.001. enhD, distal enhancer-like signature; enhP, proximal enhancer-like signature; prom, promoter-like signature; CTCF, CTCF-only; K4m3, DNase-H3K4me3. (**D**) Effects of hSTRs on gene expression. (**E**) Differential abundance of genes affected by aSTRs in significantly enriched Kyoto Encyclopedia of Genes and Genomes (KEGG) pathways. ***P < 0.001; *P < 0.05 based on 2000 permutations. FcγR, Fcγ receptor; mTOR, mammalian target of rapamycin. (**F**) Expression level of tissue-enriched genes affected by aSTRs. Tissues with fewer than five tissue-enriched genes are grouped as “Others” for presentation.

To further identify pSTRs that may contribute to the genetic determinants of high-altitude adaptation, we performed linear regression analyses of pSTR dosages against multiple environmental variables characteristic of high-altitude regions, including altitude, temperature, and solar radiation. This analysis was restricted to a down-sampled GSRD dataset comprising 874 unrelated TB speakers and Han Chinese individuals (data S1). These individuals were sampled from regions ranging from sea level to ~4500 m above sea level and were evenly distributed across varying environmental conditions, thereby reducing the risk of overfitting in the regression models (fig. S27A). Our results showed that ~10% of pSTRs were significantly associated with at least one environmental variable (Benjamini-Hochberg–adjusted *P* < 0.05), with substantial overlap among independent tests (fig. S27, B and C). To account for collinearity and variability in measurements, we applied multivariate adaptive shrinkage to integrate the results of independent tests, generating combined posterior means (CPMs) of association effects on the composite environment (fig. S27D). We identified 17,195 significant high-altitude environment-associated STRs (hSTRs), defined by |CPM| > 0.3 and Benjamini-Hochberg–adjusted Fisher’s *P* < 0.05 (data S6). Notably, hSTRs that exhibited population differentiation among TB speakers generally displayed stronger associations with environmental variables, as indicated by higher absolute CPM values (Fig. 4A and fig. S28). We found that the absolute CPM values of hSTRs were significantly correlated with Rst_TIB-HAN_ (Pearson’s *r* = 0.439, *P* < 2.2 × 10^−16^), Rst_YI-HAN_ (Pearson’s *r* = 0.114, *P* < 2.2 × 10^−16^), and Rst_OTB-HAN_ (Pearson’s *r* = 0.067, *P* = 3.8 × 10^−8^), whereas no correlation was found for Rst_TUJ-HAN_ (*P* = 0.370). These findings highlight the connections between population differentiation and local adaptation in TB speakers, particularly among Tibetans, which concord with the higher natural selection stress of their living environments.

Although most hSTRs were intergenic or non-CDS variants, we identified 28 hSTRs that resided in the CDS regions of genes (Fig. 4B). Using Fisher’s exact test, we assessed their enrichment across different STR categories and found that coding hSTRs were enriched in TB-divergent pSTRs (odds ratio = 3.40, *P* = 0.024), eQTL loci (odds ratio = 2.73, *P* = 0.010), and eSTRs (odds ratio = 2.50, *P* = 0.094) compared to non-CDS hSTRs, suggesting an important role in gene functions. In general, those coding hSTRs exhibited high levels of polymorphisms with a mean heterozygosity of 0.382 ± 0.167, thus potentially altering protein structures and contributing to diverse phenotypes. For example, the highest CPM of coding hSTRs was observed for the dimorphic chr14:105529713(CCG)n in exon 2/2 of *TMEM121* (CPM = 0.617, Benjamini-Hochberg–adjusted Fisher’s *P* = 2.3 × 10^−23^). We used AlphaFold3 (*55*) and D-I-TASSER (*56*) to predict the structures and functions of the two repeat-containing TMEM121 isoforms. We found that the (CCG)5/(CCG)6 repeats encoded a polyproline in the C-terminal compositional bias region with high solvent accessibility, potentially influencing the binding affinity between TMEM121 and its ligands (fig. S29A). Furthermore, the (CCG)5/(CCG)6 repeats were correlated with the altitude of the sampling sites (fig. S29B), and the longer repeat was more prevalent among Tibetans (fig. S29C), which probably plays a role in their local adaptation. Other affected genes are also tightly connected to critical biological functions. For example, the *EOMES* gene is highly expressed in CD8^+^ T and natural killer cells to regulate the expression of downstream genes. A suppressed regulon activity of *EOMES* was observed during high-altitude mountaineering, reflecting the impaired immune functionality (*57*). In addition, the *NOTCH4* gene has been implicated in vascular remodeling to protect against hypoxic injury (*58*). Collectively, these results support the roles of coding STRs as essential contributors to protein diversity, putatively bearing evolutionary significance (*12*).

To elucidate the potential mechanisms by which STRs modulate the genetic basis of high-altitude adaptation, we analyzed their roles within candidate ENCODE cCREs. We identified 1281 nonredundant hSTRs overlapped with cCREs, predominantly located in enhD and enhP (Fig. 4C, left inset). These cCRE-overlapping hSTRs exhibited higher heterozygosity and sequence entropy than cCRE-disjoint counterparts (fig. S30), suggesting their essential contribution to the variability of cCREs. We assessed phastCons scores for sequences centered on both cCRE-overlapping and cCRE-disjoint hSTRs (Fig. 4C). Within a ±2000-bp window, sequences flanking cCRE-overlapping hSTRs showed significantly higher mean phastCons scores (two-sided Wilcoxon test, *P* < 2.2 × 10^−16^). Similar patterns of conservation were also observed using smaller window sizes (Fig. 4C, right inset). Overall, regions centered on hSTRs displayed lower phastCons scores, with a sharp increase within the ±100-bp flanking regions. These findings suggest that hSTRs within cCRE regions are subject to relatively weaker evolutionary constraints, which may function as modulators that fine-tune the sequence and functional variability of genomic cis-regulatory elements. To illustrate this mechanism, we conducted in silico mutations on three enhD-overlapping hSTRs that were identified as eSTRs for *PLCG1*, *EDN1*, and *VEGFA* genes in the HIF pathway and predicted their impact on local epigenetic patterns using the deep learning–based Sei model (*59*). This analysis revealed that these hSTRs influence histone modifications and transcription factor (TF) binding affinities of the local DNA sequences (fig. S31). Our findings reveal the widespread involvement of environment-associated STRs in shaping the variability of genomic cis-regulatory elements, thereby providing a foundation for gene expression regulation and human phenotypic diversity.

We next examined the impact of hSTRs on gene expression to further explore their biological importance. Our analysis revealed that hSTRs were significantly enriched in eQTL loci (Fisher’s exact test, odds ratio = 1.33, *P* < 2.2 × 10^−16^) and eSTRs (Fisher’s exact test, odds ratio = 1.44, *P* < 2.2 × 10^−16^) compared to pSTRs not associated with environmental conditions. A total of 3380 hSTRs were identified as eQTLs for 2740 protein-coding genes, of which 918 were eSTRs (Fig. 4D). These affected genes are involved in multiple important biological functions, such as oxygen sensing (*EGLN1*), energy metabolism (*ATP6V1E2*, *ATP5PO*, *ACOT2*, *FAHD1*, and *HSD17B12*), mitochondrial function (*TUFM*, *MRPL53*, and *SMDT1*), and cell proliferation (*PIGF* and *SPATA20*). We categorized these hSTRs as potential aSTRs, given their dual association with high-altitude environments and gene expression levels. We further analyzed their influence on specific biological pathways. Compared to randomly sampled background genes, the expression levels of genes in nine Kyoto Encyclopedia of Genes and Genomes (KEGG) pathways were significantly influenced by aSTRs (2000 permutations, Benjamini-Hochberg–adjusted *P* < 0.05; Fig. 4E and data S7). Notably, the lysosome pathway (hsa04142) exhibited the most pronounced disturbance (differential abundance = −1.78, 2000 permutations, *P* = 5.0 × 10^−4^). Lysosomes play a critical role in maintaining cellular homeostasis by degrading surplus or malfunctioning proteins and organelles under hypoxic conditions, such as mitochondrial self-digestion (*60*). In addition, gene expression in the one-carbon pool by folate (hsa00670) and purine metabolism (hsa00230) pathways was also affected. Folate is essential for cell proliferation and metabolic processes, and alterations in folate biosynthesis and utilization may serve as compensatory mechanisms for its degradation under high-altitude ultraviolet exposure (*61*). Meanwhile, purine levels increase under hypoxic and hypothermic conditions due to adenosine 5′-triphosphate (ATP) degradation, ultimately leading to increased uric acid production and oxygen free radicals. This observation also aligns with the high prevalence of gout in Tibetan highlanders (*62*).

We further explored the expression of genes associated with aSTRs across 29 human tissues from the Genotype-Tissue Expression (GTEx) project (*63*). We identified 112 nonredundant tissue–enriched genes in 21 tissues. Among these, more than five tissue-enriched genes were observed in testis (40 genes), spleen (16 genes), brain (9 genes), liver (7 genes), and muscle (6 genes) (Fig. 4F). These tissue-enriched genes play essential roles in the normal development and functional regulation of these tissues. For example, the ion channel *CALHM6* is predominantly expressed in the spleen and controls cellular cross-talk at the immunological synapse during bacterial infection (*64*). The liver-enriched gene *SERPINA1* encodes SerpinA1, a serine protease inhibitor that regulates preadipocyte proliferation and uncoupling protein 1 expression, thereby enhancing energy and glucose metabolism (*65*). Together, these results highlight the critical involvement of aSTRs in regulating gene expression within key biological pathways, potentially through tissue-specific regulatory mechanisms.

### STR expansion dynamics

**Fig. 5.**
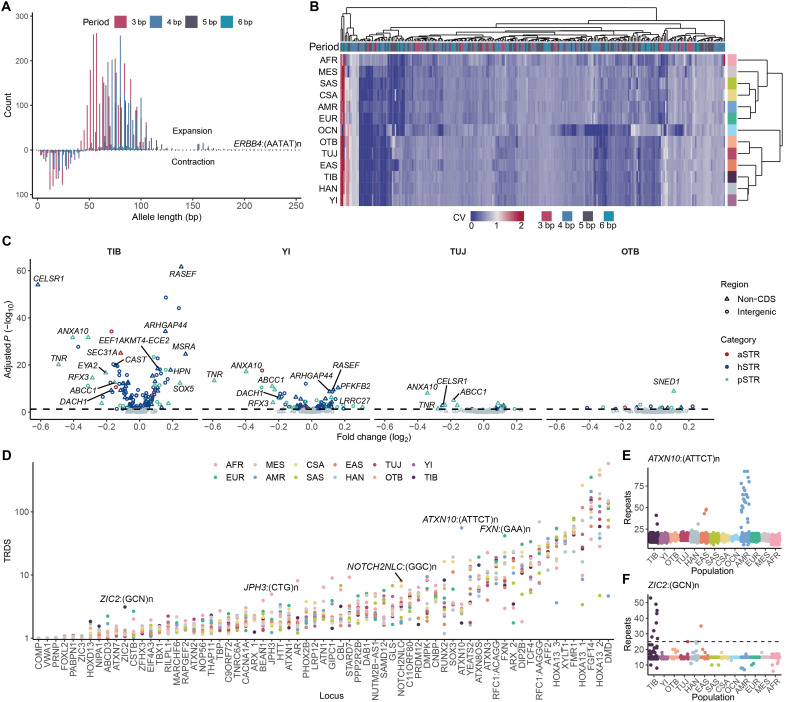
STR expansions. (**A**) Length distribution of contracted and expanded STR alleles. (**B**) Clustering of highly expanded STRs (expansion score ≥ 2) across population groups based on locus variability. (**C**) Pairwise comparison of lengths between the TB-speaking and Han Chinese populations for highly expanded STRs. STRs are labeled by the corresponding gene name. The dashed line indicates the threshold of Benjamini-Hochberg–adjusted *P* = 0.05. (**D**) Pairwise TRDS scores of different pathogenic loci compared to randomly sampled null distribution controls. (**E** and **F**) Repeat unit distributions of *ATXN10*:(ATTCT)n (E) and *ZIC2*:(GCN)n (F) across population groups. The dashed line indicates the pathogenic threshold.

STR expansion represents an important evolutionary driving force and is associated with dozens of human disorders (*2*, *3*, *66*). We investigated the underexplored STR expansion patterns in TB speakers to unravel their potential implications for local adaptation and health outcomes. Focusing on pSTRs with motif lengths of ≥3 bp, we identified 4741 expanded alleles and 897 contracted alleles across 2541 pSTR loci. Among these, we detected 82 nonredundant expanded alleles at 32 loci that exceeded the read length of WGS (150 bp). Regenotyping these loci via ExpansionHunter (*67*), a sequence graph–based tool for accurate long STR expansion analysis from short-read data, revealed a significant linear correlation between the two datasets (Pearson’s *r* = 0.433, *P* = 2.0 × 10^−11^; fig. S32A). The longest expansion occurred at chr2:212192150(AATAT)n in the first intron of *ERBB4* (Fig. 5A and fig. S32B), which encodes a receptor tyrosine kinase enriched in interneurons and is linked to familial amyotrophic lateral sclerosis 19 (*68*). Most expansions and contractions occurred at tri- and tetranucleotide loci (Fig. 5A and fig. S32C). In addition, CDS expansions and contractions were exclusively observed at tri- and hexanucleotide repeats (fig. S32D). The highest number of expanded alleles was observed in the African population, while TB-speaking and Han Chinese populations exhibited similar counts of these outliers (fig. S32E).

We subsequently used the expansion score to evaluate the extent of STR expansion. As expected, CDS pSTRs demonstrated lower expansion levels compared to those in non-CDS and intergenic regions (fig. S32F). In addition, 269 highly expanded loci (expansion scores ≥ 2) were identified in at least one population, where TB speakers generally exhibited similar variation patterns to those of Han Chinese and other East Asians (Fig. 5B). We found that 164 of these expanded loci exhibited significant differences in mean allele lengths between TB speakers and Han Chinese (two-sided Wilcoxon test, Benjamini-Hochberg–adjusted *P* < 0.05). In addition, 124 loci were associated with high-altitude environments (i.e., hSTRs), including four loci that were linked to gene expression and considered potentially adaptive (i.e., aSTRs) (Fig. 5C). Overall, more outlier pSTRs were identified in populations with high TB-specific ancestry, such as Tibetans and Yi people. Notably, pSTRs in the *ANXA10* and *TNR* genes were frequently identified as outliers. The *ANXA10* gene regulates cellular growth and signal transduction pathways and has been implicated in cancer cell propagation (*69*). Furthermore, the *TNR* gene, which is specifically expressed in the central nervous system, plays crucial roles in neurite outgrowth, neural cell adhesion, axonal guidance, and synaptic plasticity (*70*). Therefore, the observed expansion of these pSTRs in TB speakers may contribute to local adaptation and health-related traits in these populations.

We next analyzed the variation of 67 known pathogenic STRs and evaluated their AF distributions using the TRDS score. Compared to randomly permuted null distributions, most of pathogenic STRs exhibited apparent deviations across populations, among which several loci showed population-specific expansion patterns as previously reported (Fig. 5D). For instance, expansions of the (ATTCT)n repeats in intron 9 of the *ATXN10* gene, which causes spinocerebellar ataxia type 10 (SCA10), were most frequently observed in American samples (Fig. 5E). This observation is consistent with the higher prevalence of SCA10 in American populations and supports the presence of a common founder effect (*71*). Similarly, expansions of *JPH3*:(CTG)n, *NOTH2NLC*:(GGC)n, and *FXN*:(GAA)n are more commonly observed in Africans, East Asians, and Europeans, respectively, in agreement with earlier reports (*72*). Furthermore, we identified 14 alleles that expanded beyond the pathogenic threshold for the (GCN)n repeat in exon 3/3 of the *ZIC2* gene, which is linked to holoprosencephaly 5 (HPE5) phenotypes (*73*). All of these expansions were detected in East Asian samples, with 8 of 14 (57.1%) occurring in Tibetans (Fig. 5F and fig. S33). However, none of the eight carriers displayed HPE5-related phenotypes, suggesting a potential Tibetan-specific founder effect at this locus that requires further investigation in future studies.

### STR variations contribute to human complex traits

**Fig. 6.**
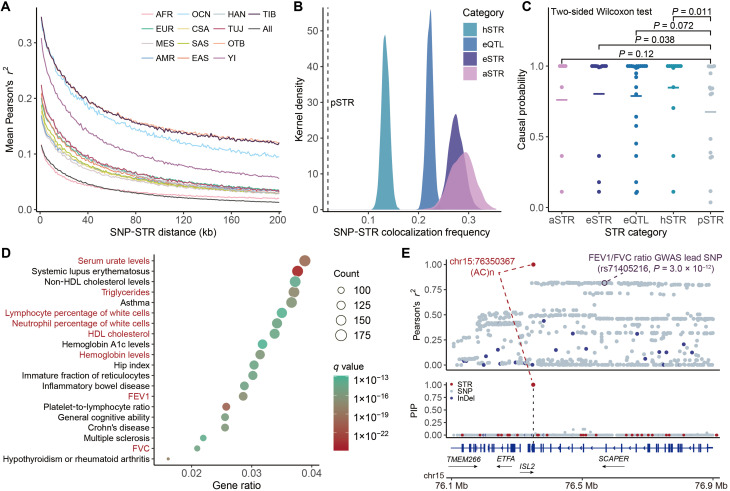
Implication of STR variations in human complex traits. (**A**) LD decay patterns between pSTRs and GWAS risk SNPs across diverse population groups. (**B**) Frequency of GWAS risk SNP and STR colocalization across different STR functional categories. The dashed line represents the mean colocalization frequency for randomly permuted pSTRs. (**C**) Causal probabilities of STRs in the PheWAS study reported by Manigbas *et al.* (*8*), stratified by STR functions. Horizontal bars represent mean causal probabilities. (**D**) Enrichment analysis of GWAS Catalog traits linking to GWAS risk genes tagged by eQTL loci. Traits showed significant difference between Tibetan and Han Chinese reported by He *et al.* (*18*) are marked in red. (**E**) Regional plot of the *SCAPER* gene showing the LD pattern (top track) and fine-mapping posterior probabilities (middle track) for chr15:76350367(AC)n, SNPs, and InDels.

STRs have been proposed as major contributors to complex traits and polygenic disorders (*3*). To explore whether these functionally relevant STRs identified in this study can drive the genetic determinants of complex traits and disease risks, we evaluated the linkage disequilibrium (LD) between pSTRs and risk SNPs from the GWAS Catalog (*74*). As a result, we identified 18,391 pSTRs that were colocalized with GWAS risk SNPs with high LD (Pearson’s *r*^2^ > 0.8) in 4846 GWAS datasets from diverse ancestries. When the analysis was restricted to 381 GWAS datasets from individuals of East Asian ancestry, 6491 pSTRs were found to colocalize with GWAS risk SNPs. The traits with the highest numbers of tagged pSTRs included height, body mass, bone mineral density, blood pressure, and blood/serum biochemical markers (fig. S34). Furthermore, we observed a marked decay in LD with increasing physical distance between SNPs and STRs, and this pattern varied in a population-dependent manner (Fig. 6A and fig. S35A). Notably, higher LD was detected between pSTRs and GWAS risk SNPs in Tibetans, Yi, and other TB speakers, highlighting their distinct genetic LD patterns that may account for the missing heritability in GWAS studies.

We next linked the regulatory effects of STRs to their implications in complex traits. Among these tagged pSTRs identified in GWAS datasets from diverse ancestral populations, 6791 (36.9%) were classified as eQTLs, and 2307 (12.5%) were associated with high-altitude environments (i.e., hSTRs). Compared to randomly permuted pSTRs, STRs that potentially harbor functional or adaptive significance were more frequently tagged by GWAS risk SNPs (Fig. 6B and fig. S35B). To further explore the connection between STR functions and human traits, we intersected our findings with fine-mapped causal STRs characterized by a PheWAS study conducted on UK Biobank participants (*8*), identifying 33 fine-mapped STRs that influence 25 phenotypes. We observed higher causal probabilities in the fine-mapping analysis for these potentially functional or aSTRs (Fig. 6C). These results suggest that STRs can drive human phenotypic variation through the regulation of gene expression. To validate this, we examined the enrichment patterns of risk genes tagged by eQTL loci in certain GWAS traits. We found that these genes were enriched in 1208 GWAS traits (*q* < 0.05; data S8). Among these, several traits showed significant differences between Tibetans and Han Chinese as reported by He *et al.* (*18*), such as serum lipid levels [triglycerides and high-density lipoprotein (HDL) cholesterol] and lung functions [forced expiratory volume in one second (FEV1) and forced vital capacity (FVC)], which may reflect phenotypic adaptation in highland Tibetans (Fig. 6D and data S8).

To further identify STRs contributing to human phenotypic variation, we performed a fine-mapping analysis to infer the causal probability of STRs for the expression levels of GWAS risk genes, along with nearby SNPs and insertions/deletions (InDels). As a result, we identified 32 potential causal STRs linked to 98 traits, which exhibited a high posterior inclusion probability (PIP) of >0.5 and had the highest PIP scores among all STR, SNP, and InDel variants tested (data S9). For example, chr15:76350367(AC)n was significantly associated with the expression of *SCAPER* (Benjamini-Hochberg–adjusted *P* = 1.1 × 10^−8^) and showed strong colocalization with rs71405216 (Pearson’s *r*^2^ = 0.817), a risk SNP associated with FEV1/FVC ratio (*P* = 3.0 × 10^−12^) (*75*). However, this STR was the only variant identified as causal (PIP = 1.00), representing a promising candidate for mediating lung functions (Fig. 6E). Another example is chr17:51297965(AC)n, an hSTR (CPM = 0.416, Benjamini-Hochberg–adjusted Fisher’s *P* = 1.9 × 10^−9^). This STR exhibited the highest causal probability for the expression of *MBTD1* (PIP = 0.568) and strongly colocalized with the risk SNP rs4794213 (Pearson’s *r*^2^ = 0.957), which was linked to coronary artery disease (*P* = 4.0 × 10^−8^) (*76*). Collectively, our findings highlight the functional and evolutionary importance of STR variations in driving human phenotypic diversity.

## DISCUSSION

Large-scale human cohort studies have highlighted the critical roles of STRs in human health and diseases. However, existing genome-wide STR research has predominantly focused on populations of European ancestry (*6*–*8*, *77*), thereby perpetuating underrepresentation of other ancestral groups and health disparities. This study presents the most comprehensive catalog of STR variations in TB-speaking populations residing in the Qinghai-Tibet Plateau and surrounding regions. By analyzing over 1.1 million genome-wide STRs, we characterized more than 570,000 pSTRs from 7876 deep WGS samples, including over 50,000 pSTRs exclusively identified in newly sequenced individuals from the GSRD project. Using this high-quality STR dataset, our analyses revealed nonuniform distributions of pSTRs across different genomic regions, showing a marked enrichment in active chromatin states and putative regulatory regions. In contrast, pSTRs located in CDS regions exhibited reduced diversity and stronger mutational constraints, likely reflecting their roles in critical biological processes. Furthermore, the genome-wide dataset enabled a deeper understanding of STR variation patterns in TB-speaking populations. For example, we observed a higher prevalence of LoF variants in Tibetan genomes affecting genes under high genomic constraint. LoF variants are recognized as a potential source of adaptive advantages in organisms (*78*, *79*). Thus, their increased occurrence in Tibetan genomes might reflect genomic responses to the ubiquitous environmental stressors associated with high-altitude living. In future studies, elucidating the mechanisms and functional consequences of these LoF variants could further enhance our understanding of human genomic regulation under extreme environmental conditions. Collectively, our findings not only expand the genetic resources available for STR variations in East Asians, particularly among previously understudied TB-speaking populations, but also provide a foundation for exploring the functional and evolutionary significance of STRs in human adaptation.

Using the established STR dataset, we redefined the footprints of STR variations, revealing complex genetic connections and admixture patterns among TB speakers. Notably, we found that Tibetans, Yi, and other TB speakers exhibited relatively close genetic relationships, whereas Tujia individuals showed greater genetic affinity with Han Chinese individuals. In addition, we observed evident shared ancestry components between TB speakers and other ethnolinguistically distinct populations, including those from Sinitic-, Tai-Kadai–, and Hmong-Mien–speaking populations. These findings corroborate previous SNP-based studies, reinforcing the complex demographic histories and admixture events that have shaped TB-speaking populations (*28*, *30*, *34*, *45*). Furthermore, we identified a substantial number of pSTRs that were divergent between TB speakers and Han Chinese individuals. The most divergent variants were predominantly located in genes and genomic regions with potential high-altitude adaptive effects. For example, several highly divergent pSTRs near the *RHOQ*, *ATP6V1E2*, and *PIGF* genes are located in proximity to a known Tibetan-specific deletion variant that disrupts the enhancer of *EPAS1* (*27*), a well-characterized high-altitude adaptive gene under strong selection (*21*, *22*). These divergent pSTRs likely reflect multiple selection events in this genomic region, indicating a synergetic effect in shaping high-altitude adaptive phenotypes.

To further extrapolate the biological functions of TB-divergent pSTRs, we performed the eQTL analysis using RNA sequencing (RNA-seq) data from multiancestry lymphoblastoid cell lines. We found that a substantial proportion of pSTRs were significantly associated with the expression of nearly half of the expressed genes, and the regulatory potentials of these eQTLs were largely consistent across different cell lines. This supports the previous finding that most eQTLs are shared across tissues (*80*), emphasizing the extensive regulatory effects of STRs on gene expression. We found that TB-divergent pSTRs were more frequently associated with gene expression than random loci, which confirmed their regulatory activity and suggested potential biological roles in the local adaptation of TB speakers. Overall, this study provides an STR-focused perspective on the genetic architecture of TB-speaking populations, which has been largely shaped by demographic events, genetic interactions, and environmental pressures.

The distinct evolutionary trajectories of STRs across species highlight their critical roles in mediating adaptive traits and phenotypes that confer survival advantages (*2*, *12*, *66*). In this study, we characterized widespread associations between pSTRs and high-altitude environmental conditions. We found that TB-divergent STRs generally exhibited stronger association effects, underscoring the close relationship between genetic differentiation and local adaptation to extreme high-altitude environments. Notably, dozens of these environment-associated STRs were located within the CDS regions of genes. These coding STRs were largely divergent in TB speakers and tightly associated with gene expression levels, thus potentially contributing to local adaptation through directly influencing protein sequences. Coding STRs are known to facilitate adaptive evolution across a wide range of species from plants to mammals (*12*, *14*, *15*). Specifically, human-specific coding STR expansions also played fundamental roles during primate evolution (*66*). In addition, previous studies revealed extensive associations between coding STRs and human phenotypes, such as blood traits, cardiovascular function, cognition, and psychiatric outcomes (*6*–*8*). Compared to these studies, our findings highlight the connection between human coding STR variations and high-altitude environmental conditions, thereby broadening the scope of their biological implications.

It is estimated that 10 to 25% of promoters in eukaryotic genomes harbor unstable STR sequences with high affinities for TFs, leading to their strong regulatory activities on gene expression (*81*). Meanwhile, the presence of STRs is associated with increased silencer activities in T cells (*82*). These findings emphasize the extensive influence of STRs on the functions of cis-regulatory elements. In this study, we found that environment-associated STRs frequently overlapped with known cCREs. These STRs displayed relaxed evolutionary constraints and potentially functioned as evolvable entities capable of modulating the activity of cis-regulatory elements; thus, they may play a role in regulating gene expression and phenotypic variation. Furthermore, we found that a substantial number of environment-associated STRs were linked to gene expression, which have potentially participated in the local adaptation of highland TB speakers. By integrating their effects on gene expression and environmental associations, we found that genes involved in several key biological pathways were significantly influenced by these aSTRs, including lysosome functionality, folate biosynthesis, purine metabolism, and energy metabolism. Moreover, dozens of these affected genes exhibited tissue-specific expression and functions. These results are consistent with the multisystem phenotypic adaptations observed in indigenous highland populations, such as improved lipid metabolism, elevated inflammatory responses, and reduced serum folate and urate levels in highland Tibetans compared to Han Chinese individuals (*18*). Our findings underscore the fine-tuned genetic basis of high-altitude adaptation driven by STR variations, expanding the spectrum of genetic variants known to contribute to the local adaptation of highland TB-speaking populations.

The expansion of STR tracts is a well-recognized source of genetic diversity, and extreme STR expansions are directly linked to human disorders (*2*, *3*).WGS has emerged as a first-tier method for screening these variations (*83*). In this study, we elucidated the distinguishable expansion patterns of both nonpathogenic and known pathogenic loci within TB-speaking populations. We found that many expanded loci were associated with high-altitude environmental conditions. In addition, the outlier analysis demonstrated a functional linkage between these loci and genes of biological importance, suggesting their potential roles in local adaptation and disease susceptibility among TB speakers.

On the other hand, compared to non–TB-speaking populations, we observed distinct LD patterns between STRs and GWAS risk SNPs among different TB-speaking populations that are underrepresented in existing GWAS studies. These differences suggest discrepant susceptibilities to diseases and health-related traits across these populations. Furthermore, we found that STRs exhibiting potential functional or adaptive roles were more frequently tagged by GWAS risk SNPs and showed stronger associations with a wide range of human phenotypes. GWAS risk genes tagged by eQTL loci were significantly enriched in nominated adaptive traits in highland Tibetans, such as serum lipid levels and lung functions, highlighting their indispensable contributions to human phenotypic adaptation. We also identified dozens of STRs as the candidate causal variants driving corresponding GWAS traits through the fine-mapping analysis. These casual STRs were often linked to more than one trait, suggesting a pleiotropic effect of STRs in mediating human phenotypic variation. Collectively, these findings emphasize the importance of incorporating STR analysis to address the missing heritability problem in future GWAS and PheWAS studies.

In summary, this study provides comprehensive insights into STR variations in high-altitude populations and demonstrates their potential role in adaptive evolution. However, there are several limitations that warrant further improvement in future research. First, current genotyping tools heavily rely on catalogs of known STRs derived from the reference genome. However, the precise number of STRs in the human genome remains undetermined due to inconsistencies in definitions and curation pipelines, which often fail to capture the full diversity of STR variations across human populations (*84*). Graph-based approaches constructed upon pangenomes, which integrate multiple genome assemblies from diverse populations, offer a promising avenue for the discovery and characterization of previously unknown STRs (*85*). In addition, accurately resolving long expansions and variations in motif composition represents a major challenge for short-read WGS technologies. In contrast, third-generation sequencing technologies generate longer reads capable of spanning most repeat expansions, thereby enabling high-resolution characterization of noncanonical STRs (*86*). Moreover, although this study highlights a strong association between STR variations and high-altitude adaptive phenotypes, systematic functional validation remains a major technical challenge. Comprehensive integration of multiomics datasets (*87*), such as those from the ENCODE and Human Cell Atlas projects (*88*), could provide fundamental insights into the functional roles of STRs across tissues and cell types. In addition, high-throughput methods such as massively parallel reporter assays (*82*) may also facilitate large-scale functional investigations of genome-wide STRs. Last, given the underrepresentation of TB speakers in existing genomic studies, another caveat is the Eurocentric bias in GWAS and PheWAS data used here. Therefore, future studies should prioritize the unbiased exploration of genetically diverse populations, including TB-speaking populations not present in this work, to capture the full spectrum of genetic variation and advance our understanding of human health and disease.

## MATERIALS AND METHODS

### Sample enrollment and WGS

This study was approved by the Medical Ethical Committee of West China Hospital, Sichuan University [approval number: 2021 Audit (190)] and conducted in compliance with the Declaration of Helsinki. All newly reported WGS data were obtained from the GSRD project, a research initiative designed to explore the molecular mechanisms underlying rare diseases and identify genetic risk factors within the Chinese population using high-coverage WGS technology. At recruitment, venous blood was collected from each participant after written informed consent was obtained. Genomic DNA was extracted using the MGIEasy Genomic DNA Extraction Kit (MGI Tech, Shenzhen, Guangdong, China) on an MGISP-NEX automated nucleic acid extraction system. The sequencing libraries were prepared using the MGIEasy Universal DNA Library Prep Set (MGI Tech) according to the manufacturer’s protocol. Specifically, qualified DNA was ultrasonically fragmented, size selected, end repaired, and 3′-adenylated. MGIEasy adapters were ligated to the A-tailed DNA fragments, followed by polymerase chain reaction (PCR) amplification. Single-strand amplicons were separated and cyclized for rolling circle amplification to produce DNA nanoballs, which were quantified and loaded into patterned nanoarrays on the DNBSEQ-T7 platform (MGI Tech) for 150-bp paired-end sequencing. The raw sequencing images were converted to FASTQ files using DNBSEQ base-calling software with default parameters. Additional FASTQ files from 33 Tibetan and 5 Sherpa genomes were obtained from Lu *et al.* (*34*). The sequencing reads were trimmed and filtered using fastp v0.20.0 (*89*) with parameters “--cut_tail --cut_front -l 45 -n 10”. Clean reads were mapped to the GRCh38 reference genome by Burrows–Wheeler aligner-maximal exact matches (BWA-MEM) v0.7.17 (*90*) and sorted by coordinates via SAMtools v1.19 (*91*). Duplicated reads were marked using Picard v2.20.1 (https://github.com/broadinstitute/picard). The mapping metrics of the BAM files were evaluated using the CollectMultipleMetrics and CollectWgsMetrics modules of Picard v2.20.1.

Before the main analysis, we included a total of 4259 individuals with self-reported TB-speaking or Han Chinese ancestries in the GSRD cohort. Individuals diagnosed with severe developmental or neurological disorders were excluded to avoid overestimation of STR variations caused by pathogenic mutations, as STRs are well-established contributors to these diseases. In addition, the following criteria were applied to exclude unqualified WGS samples: mean sequencing depth < 25×, fraction of bases with > 20× depth < 80%, mean fragment insert size < 250 bp, PCR duplication rate > 10%, mapping rate < 95%, and mismatch rate > 1%. As a result, high-quality WGS data for 3808 individuals in the GSRD project were analyzed in this study, comprising 1997 Han Chinese and 1811 TB-speaking individuals (981 Tibetan, 475 Yi, 253 Tujia, 37 Qiang, 26 Bai, 15 Hani, 13 Naxi, 4 Lisu, 3 Moinba, 2 Lahu, 1 Pumi, and 1 Jingpo individuals). We also included 3202 samples from the e1kGP project and 828 samples from the HGDP project as a global reference panel. High-coverage CRAM files of these samples were aligned to GRCh38 and were obtained via the International Genome Sample Resource (www.internationalgenome.org/). Details of the samples analyzed in this study are provided in data S1.

### Genome-wide STR genotyping and quality control

To mitigate the limitations of single-method approaches, we used the EnsembleTR (*35*) pipeline that integrates results of HipSTR (*36*) and GangSTR (*37*) for genome-wide STR calling. The genome-wide STR catalogs “GRCh38.hipstr_reference.bed.gz” curated for HipSTR (https://github.com/HipSTR-Tool/HipSTR/) (*36*) and “hg38_ver17.bed.gz” for GangSTR (https://github.com/gymreklab/gangstr/) (*37*) were combined using BEDTools v2.3.0 (*92*). STRs duplicated between the two catalogs, with reference alleles of >150 bp, or of mononucleotide repeats, were excluded from further analysis. We applied HipSTR v0.6.2 (*36*) with parameters “--def-stutter-model --min-reads 3” and GangSTR v2.5.0 (*37*) with “--max-proc-read 100,000” to batches of approximately 100 samples. Batch VCF files generated by each tool were independently merged using the mergeSTR module in TRTools v6.0.1 (*93*). The merged VCFs were filtered using dumpSTR in TRTools (*93*) with parameters “--min-locus-callrate 0.5 --min-locus-hwep 1e-20.” For GangSTR VCFs, additional filters “--gangstr-filter-only-spanbound” and “--gangstr-filter-badCI” were applied to exclude unreliable calls. We used VCFtools v0.1.16 (*94*) to evaluate the genotype missingness for each sample and excluded any samples with a missing rate greater than 0.2. We then combined the results of the two STR genotyping tools using EnsembleTR v1.0.0 (*35*) to obtain consensus genotypes for each locus, following the pipeline provided by Jam *et al.* (*35*). Calls with consensus call scores below 0.9 and loci with missing rates exceeding 0.5 were further removed from analysis using BCFtools v1.19 (*91*). Per-locus statistics were computed on the basis of STR allele lengths using the statSTR module of TRTools (*93*). For single-nucleotide variant calling, we used GATK v4.1.2.0 (*95*) according to the Best Practices Workflows for germline short variant discovery (https://gatk.broadinstitute.org/hc/en-us/articles/360035535932-Germline-short-variant-discovery-SNPs-Indels).

Using the high-quality STR dataset, we aimed to characterize population-specific STR variations and explore their functional implications within TB-speaking populations. Given that STR variations may be associated with certain diseases and confounding factors, we evaluated the potential influence of key covariates, including age, sex, and disease status, on STR detection. Our results indicated that these factors had negligible effects on our major findings, including STR expansion, contraction, and pLoF variations (text S2). Therefore, in the main analysis, we did not stratify individuals by specific disease status to maximize statistical power.

### Relatedness inference and MI analysis

Sample sex was determined by estimating the ploidy of sex chromosomes using the GATK DetermineGermlineContigPloidy module (*95*). Pairwise kinship coefficients were calculated using PLINK v1.9 (*96*), and first-degree family networks were reconstructed by PRIMUS v1.9.0 (*97*). Parent-child trios were validated by integrating both genetic and recorded pedigree relationships. To ensure a maximally unrelated sample set for population genetics and association analyses, we applied PRIMUS to exclude individuals with third-degree or closer familial relationships, retaining 5976 unrelated samples. To assess the reliability of STR genotypes, we evaluated the MI patterns of STR alleles transmitted from parents to offspring in 576 GSRD and 602 e1kGP parent-child trios. In our analysis, MI was deemed valid for an STR locus when genotypes were available for all three members of the trio, and both child alleles could be assigned to one parent each. The MI consistency rate was calculated as the proportion of genome-wide STRs adhering to MI across the trios. This analysis was performed using the BCFtools (*91*) +mendelian2 plugin.

### Genomic annotation

The gene and transcript annotation file for GRCh38 was downloaded from the Ensembl Genome Browser release 111. Specifically, we defined upstream and downstream genic regions by extending 2 kb from the transcription start sites and transcription termination sites of genes. Genomic locations of STR loci were determined by intersecting their physical locations in the reference genome with the processed gene set using BEDTools v2.30.0. Only canonical transcripts were used to define genomic regions. Genes were categorized into different LOEUF deciles based on their intolerance to LoF variations as described by Karczewski *et al.* (*40*). Enrichment analysis for genes in the first three LOEUF deciles containing CDS pSTRs was conducted using clusterProfiler v4.12.6 (*98*). The consequences of STR variations were predicted using Ensembl VEP v111 (*41*). Variants annotated as frameshift, splice-acceptor, splice-donor, start-lost, and stop-gained were classified as pLoF variants.

### Population structure and admixture analysis

We conducted PCA on the pSTR dosage matrix, defined as the summed repeat units of sister alleles, to investigate the genetic structure of different populations. For this analysis, we only kept hypervariable loci (heterozygosity > 0.1) with SDs of dosages within the 99th percentile. Missing values were imputed using the median dosage of each locus. PCA was performed on the pSTR dosage matrix using pcaone v1.0.0 (*99*). UMAP analysis was conducted on the top five principal components using UMAP v0.2.1 (*100*). In addition, PCA was carried out on biallelic SNPs via PLINK v1.9 (*96*) using parameters “--pca --maf 0.05” after LD-pruning by “--indep-pairwise 200 25 0.4”.

To estimate the genetic admixture of STR variations among the investigated populations, we calculated the ancestry components of unrelated individuals using the top 10 principal components of pSTR dosages with Rye v1.0 (*101*). For comparison, an unsupervised model-based clustering analysis was performed using ADMIXTURE v1.3.0 (*102*) on the biallelic SNP dataset. In this analysis, SNPs with low population frequency, violating Hardy-Weinberg equilibrium, or in LD were excluded via PLINK v1.9 (*96*) with the parameters “--maf 0.05 --hwe 1e-8 --indep-pairwise 200 25 0.4.” The ADMIXTURE program was executed with the parameters “-B100 --cv = 10” while varying the number of populations (*K*) from 2 to 20. The optimal model was determined on the basis of the cross-validation error.

### Genetic distance and locus divergence measurement

The tandem repeat Rst was calculated as $\text{Rst}=\frac{\bar{S}-S_{w}}{\bar{S}}$ , where $\;\bar{S}$ represents the mean STR dosage variances across investigated populations and $S_{w}$ denotes the within-population variance (*103*). We defined TB-divergent pSTRs as those with an Rst of >0.1 (approximately the top 0.1% of total comparisons) between any TB-speaking populations and Han Chinese (the GSRD-HAN group). In addition, to quantify the difference in AF distributions of pSTRs between populations, we measured the TRDS using the 2-Wasserstein distance metric implemented in waddR v1.18.0 (*104*), following a similar approach to Cui *et al.* (*77*). Only hypervariable loci with a heterozygosity of >0.1 were considered in these analyses. To minimize the impact of unbalanced sample sizes across populations, we randomly subsampled each population group to a maximum of 100 unrelated individuals for Rst and TRDS calculations.

### Enrichment analysis of genomic and epigenomic features

To evaluate the enrichment of STRs in genomic and epigenetic features, narrow peak BED files of histone and TF chromatin immunoprecipitation sequencing (ChIP-seq) and deoxyribonuclease sequencing (DNase-seq) data were downloaded from the ENCODE project for six cell lines, including the cultured lymphoblastoid cell line (GM12878), hepatocyte, fetal lung fibroblast (IMR-90), keratinocyte, cardiomyocyte, and neural stem cell lines. In addition, expanded 18 chromatin state data for the GM12878, IMR-90, keratinocyte, and neural stem cell lines were retrieved from the NIH Roadmap Epigenomics project (*51*) (see the “Data and materials availability” section). The association and enrichment of STR loci with respect to these genomic and epigenomic features were tested using GAT v1.3.4 (*105*), with 2000 permutations. Empirical *P* values were adjusted using the Benjamini-Hochberg method. Known genomic gaps in GRCh38 were obtained from the UCSC Genome Browser track setting (https://genome.ucsc.edu/cgi-bin/hgTrackUi?g=gap) and excluded from all enrichment analyses to avoid bias.

### eQTL analysis

To investigate the impact of STRs on gene regulation, we obtained gene expression data for 731 lymphoblastoid cell lines in the MAGE resource (*52*). The RNA-seq data were processed as described by Taylor *et al.* (*52*). Briefly, gene expression pseudocounts were quantified and adjusted to the trimmed mean of *M* values (TMM), which was transformed to a normal distribution using rank-based inverse normalization to improve the power of association analysis. To account for known and potential confounding factors, sex and the top five principal components derived from SNP genotypes were included as covariates to adjust for sex and ancestry heterogeneities. In addition, 60 hidden covariates estimated by probabilistic estimation of expression residuals (*106*) were incorporated into the analysis to control for technical and latent biological effects. Focusing on 174,744 hypervariable pSTRs with sufficient polymorphisms to drive an association signal, we performed linear regression analyses between STR dosage and the transformed TMM matrix for each pSTR within ±500 kb from the transcription start site of the target gene using the following model

Y=Xβ+Wα+ϵ

where Y represents the transformed TMM values, X and β denote *z*-score–normalized STR dosages and corresponding effect size, W and α represent the aforementioned covariates and their respective coefficients, and ε is the normally distributed error term. Linear regression was performed using the “lm” function in R v4.4.1 (*107*). For the null hypothesis control, we randomly permuted the sample identifiers and repeated the same analysis. We used a gene-level FDR threshold of 5% to identify significant eSTRs as described previously (*31*, *80*). Original *P* values were adjusted for the number of pSTRs tested per gene using the Bonferroni method. Significant pSTR-gene pairs (i.e., eQTLs) were defined with adjusted *P* < 0.05. Subsequently, we applied the Benjamini-Hochberg method to the lowest *P* values across eQTLs to obtain a gene-level FDR for the eSTR of the target gene.

The epigenetic context within eSTR regions was evaluated as a surrogate for their regulatory activities. Variants influencing gene expression are often located in proximity to the target genes, thereby increasing the likelihood of colocalization with epigenetic features. Consequently, direct enrichment comparisons between eSTRs and randomly selected control regions may produce excessive false-positive signals. To mitigate this issue, we randomly shuffled the locations of eSTRs within a ±200-kb window centered on their original genomic locations. For the null distribution, we repeated this shuffling process for genome-wide randomly sampled pSTRs with eSTRs excluded. We intersected the shuffled genomic coordinate with epigenetic marks in various cell lines, and the number of overlaps was recorded. This process was repeated 2000 times to estimate the empirical fold changes in epigenetic modifications within eSTR regions.

To evaluate whether TB-divergent pSTRs were more likely to be identified as eQTLs, we randomly sampled 10% pSTRs with Rst_TBs-HAN_ > 0.1 and Rst_TBs-HAN_ ≤ 0.1, respectively. For each group, we calculated the proportion of variants classified as eQTLs or eSTRs. This sampling procedure was repeated 2000 times to generate the empirical distributions. The between-group difference was tested using a two-sided Wilcoxon test.

### STR dosage and high-altitude environment associations

To investigate the potential roles of pSTRs in high-altitude adaptation, we examined the associations between STR dosages and multiple harsh environmental conditions in the Qinghai-Tibet Plateau, including altitude, temperature, and solar radiation. To ensure balanced sample representation across these tested conditions, we subsampled GSRD samples from each prefecture-level city to less than 5% of the total sample size. This resulted in a final dataset of 874 unrelated individuals (315 Tibetan, 138 Yi, 73 Tujia, 38 other TB speakers, and 310 Han Chinese individuals) residing in regions with elevations ranging from sea level to ~4500 m above sea level for subsequent analysis (data S1). We obtained global digital elevation model data at 500-m resolution from the General Bathymetric Chart of the Oceans website (www.gebco.net/). Monthly mean temperature (in degrees Celsius) and solar radiation (in kilojoules per square meter per day) data in the 5-min spatial resolution were downloaded from the WorldClim v2.1 database (*108*), and the annual values were calculated as the mean of the 12 monthly values.

To maximize the discovery of hSTRs, we applied linear models separately for each *z*-score–normalized environmental variable and pSTR dosages using the lm function in R v4.4.1 (*107*). For the association analysis, we used the inverse of the annual mean temperature due to its negative correlation with the other two variables. To account for potential collinearities and measurement noise among these variables, we performed multivariate adaptive shrinkage using mashR v0.2.79 as described by Urbut *et al.* (*109*). Specifically, we obtained effect sizes and SEs for each pSTR from independent association tests. Next, we estimated correlations among effect sizes using null tests to update the original data. Last, we fitted the mash model with the estimated covariance matrix. The CPMs of effect sizes were used to assess the association strength between each pSTR locus and the composite environmental variable. *P* values from independent association tests were combined using Fisher’s method and adjusted using the Benjamini-Hochberg method. We defined hSTRs as those with |CPM| > 0.3 (roughly the 90th percentile of all tested loci) and adjusted Fisher’s *P* < 0.05. Only hypervariable loci (heterozygosity > 0.1) were included in the analysis.

### Differential abundance of genes in biological pathways

To investigate the impact of STR variations on biological pathways, we focused on potential aSTRs that showed significant associations with gene expression (Bonferroni-adjusted *P* < 0.05) and high-altitude environments (|CPM| > 0.3 and Benjamini-Hochberg–adjusted Fisher’s *P* < 0.05). We assessed the enrichment of affected genes in pathways from the KEGG database using clusterProfiler v4.12.6 (*98*). Disturbances of gene expression in significantly enriched pathways (*q* < 0.05) were quantified using the formula ∑(β × CPM) , where β and CPM represent the effect sizes of STRs for gene expression and environmental association. Pathways classified under the category “human disease” were excluded from this analysis. The differential abundance was used to measure the extent of gene expression disturbance within each pathway and was calculated as follows

$$\text{Differential abundance}=\frac{\left(U-D\right)}{\sqrt{N}}$$

Here, *U* and *D* represent the number of putatively up-regulated and down-regulated genes, respectively, and *N* denotes the number of background genes in the enriched KEGG pathway. To test the statistical significance of observed differential abundance, we randomly sampled a number of *N* genes from the entire gene set to construct the null distribution for each enriched pathway. This process was repeated 2000 times to generate the empirical *P* values. Tissue-enriched genes affected by these aSTRs were characterized across 29 human tissues from the GTEx project using TissueEnrich v1.24.1 in the “Tissue-Enriched” mode (*63*).

### Cis-regulatory elements and sequence conservation analysis

Genomic locations of ENCODE cCREs (*47*) combined from all cell types were obtained from the UCSC Genome Browser (https://hgdownload.soe.ucsc.edu/gbdb/hg38/encode3/ccre/). To evaluate the impact of environment-associated STRs on the conservation of regulatory elements, we calculated the mean phastCons scores for sequences within a ±2000-bp window centered on hSTRs that overlapped with cCREs using phastCons100way.UCSC.hg38 v3.7.1 (*53*). In addition, an equal number of hSTRs that were disjoint from cCREs were randomly selected as controls. Differences in phastCons scores across varying window stretches were evaluated via the two-sided Wilcoxon test.

To investigate the influence of hSTRs on the regulatory activities of local sequences, we extracted ±2000-bp regions flanking the target hSTRs from the GRCh38 reference genome. Subsequently, in silico modifications were made to the repeat units based on the allelic distribution in our dataset. Last, we adopted Sei (*59*), a deep learning–based model for systematically predicting sequence regulatory activities, to predict the effects of these modifications on epigenetic properties and TF-binding affinities of the local DNA sequences.

### STR expansion and contraction analysis

Similar to Reis *et al.* (*110*), we adopted the following criteria to identify highly expanded and contracted alleles: (i) a motif length of ≥3 bp; (ii) a local allele expansion or contraction of ≥10 repeats; and (iii) a local allele expansion or contraction of ≥50% of the major allele within the studied population. To evaluate the extent of pSTR expansion across populations, we calculated the expansion score introduced by Press *et al.* (*111*) for each locus as (95th percentile of allele length − median allele length) / median allele length. The coefficient of variation (CV) of each expanded locus was used to quantify the variance in allele lengths among populations. Clustering analysis was performed on CV values of highly expanded loci (expansion score of ≥2 in at least one population group) using ComplexHeatmap 2.20.0 (*112*) with the “complete” agglomeration method. Mean allele lengths between TB-speaking populations and Han Chinese (the GSRD-HAN group) were compared to estimate the fold change, and allelic differences were tested using a two-sided Wilcoxon test and adjusted using the Benjamini-Hochberg method. Outliers were identified on the basis of an adjusted *P* < 0.05.

### Analysis of known pathogenic STRs

Genomic information for 67 known pathogenic STR loci was obtained from the STRipy database (*113*) (https://stripy.org/) and the GitHub repository of ExpansionHunter (*67*) (https://github.com/Illumina/ExpansionHunter/). Genotyping of these STRs across all samples was performed via ExpansionHunter v5.0.0 (*67*) in streaming mode. Individual VCF files were merged with mergeSTR in TRtools (*93*), and genotypes supported by fewer than 10 reads were filtered out using dumpSTR in TRTools (*93*). REViewer v0.2.7 (*114*) was used to visualize the read alignments of STR expansions for manual inspection.

To quantify differences between allelic distributions in the specified populations and the null distribution, we calculated the TRDS score for each locus using 500 randomly selected samples as a control. Oceania samples were excluded from the analysis due to insufficient sample size.

### GWAS trait association analysis

Summary data for GWAS risk SNPs were downloaded from the GWAS Catalog v1.0.2 (*74*). We excluded SNPs with *P* > 5 × 10^−8^ or those associated with traits related to “educational attainment,” “mathematical ability,” “intelligence,” and “income” due to their strong associations with socioeconomic factors, leaving 4846 GWAS datasets from diverse ancestral backgrounds for subsequent analysis. Genotypes of the remaining GWAS risk SNPs were converted into a dosage matrix using PLINK v1.9 (*96*). We then calculated the Pearson correlation between each GWAS risk SNP and its flanking pSTRs within a ±200-kb window. LD was quantified using the squared correlation coefficient (*r*^2^). To assess LD across different populations, we subsampled each population group to include a maximum of 100 unrelated individuals. SNP-STR pairs with *r*^2^ > 0.8 were considered to be colocalized in strong LD and used for subsequent analysis.

To test whether functional or aSTRs were more likely to be tagged by GWAS risk SNPs, we randomly sampled 10% of STR loci across different functional categories and calculated the proportion of STRs tagged by GWAS risk SNPs. This resampling procedure was repeated 2000 times to generate the empirical distribution of GWAS risk SNP tagging patterns. We also replicated these analyses using a subset of 381 GWAS datasets derived from individuals of East Asian ancestry. To further explore the contribution of STRs to phenotypic variation, we obtained summary data on fine-mapped causal STRs associated with human phenotypes from data S5 of Manigbas *et al.* (*8*), which were identified from 168,554 individuals from the UK Biobank cohort. The probabilities of STRs in various functional categories being identified as causal variants were compared against those of non-functional pSTRs using a two-sided Wilcoxon test.

GWAS trait enrichment analysis was performed on risk genes influenced by eQTL loci using clusterProfiler v4.12.6 (*98*). To further identify causal STRs contributing to human phenotypic variation, we conducted fine-mapping analysis using susieR v0.14.2 (*115*) for these risk genes. The method uses a sum of single effects model to prioritize potential causal STRs associated with the expression levels of target genes from variants in strong LD. For each target gene, we applied the susie_rss function with the parameters “L = 10, coverage = 0.95, min_abs_corr = 0.5” to all STRs, SNPs, and InDels within a ±500-kb window of the transcription start site. Variants with a PIP of >0.5 and the highest PIP scores among all tested variants were defined as causal. This analysis was limited to the 731 samples included in the MAGE resource (*52*), and the eQTL summary data for SNPs and InDels were obtained from Zenodo (https://doi.org/10.5281/zenodo.10535719).

### Statistical analysis

Statistical analyses in this study were performed in the R v4.4.1 environment and are described in Materials and Methods.

## Supplementary Materials

### The PDF file includes:

Text S1 and S2 Tables S1 to S4 Figs. S1 to S35 Legends for data S1 to S9 References
