# Supplementary material for: Short tandem repeats in populations of the Qinghai-Tibet Plateau and adjacent regions provide insights into high-altitude adaptation
Source: Sci Adv. 2025 Oct 17;11(42):eadx1590. doi: 10.1126/sciadv.adx1590 (PMC12533570; doi:10.1126/sciadv.adx1590)
Supplement: Supplementary file 1 — Text S1 and S2 Tables S1 to S4 Figs. S1 to S35 Legends for data S1 to S9 References [file sciadv.adx1590_sm.pdf]

Science Advances
AAAS

Supplementary Materials for

Short tandem repeats in populations of the Qinghai-Tibet Plateau and adjacent regions provide insights into high-altitude adaptation

Yuguo Huang *et al.*

Corresponding author: Huijun Yuan, yuanhj301@163.com; Guanglin He, guanglinhescu@163.com; Fengxiao Bu, bufengxiao@hotmail.com; Jing Cheng, chj_grace@126.com; Yu Lu, samuelluyu@163.com

Sci. Adv. **11**, eadx1590 (2025)
DOI: 10.1126/sciadv.adx1590

The PDF file includes:

Text S1 and S2
Tables S1 to S4
Figs. S1 to S35
Legends for data S1 to S9
References

Other Supplementary Material for this manuscript includes the following:

Data S1 to S9

Supplementary Text 1

Orthogonal validation of WGS-based STR genotypes

Genome-wide STR calling from whole-genome sequencing (WGS) data is challenged by the complex structures of repeat sequences and the inherent limitations of short-read sequencing (SRS). Recently, several STR genotypers utilizing SRS data have been developed and widely applied in genomic studies, such as HipSTR (*36*) and GangSTR (*37*). HipSTR leverages the stutter profile of WGS samples and a hidden Markov model to realign reads to candidate STR-containing haplotypes, which can largely reduce the impact of PCR stuttering and provide reliable results for STRs shorter than the read length (*36*). In contrast, GangSTR integrates various sequencing information, including repeat-flanking reads, fragment length, coverage, and partially repeat-overlapping reads into a unified joint likelihood framework, enabling it to handle STRs longer than the read length (*37*). In this study, we combined the strengths of both genotypers by integrating their results using the EnsembleTR (*35*) pipeline, which employs a quality-sensitive voting scheme to derive a consensus genotype for each locus. Although EnsembleTR has been shown to outperform both HipSTR (*36*) and GangSTR (*37*) across a broad range of STRs for Illumina SRS data (*35*), a comprehensive evaluation of its performance remains limited, particularly for SRS data generated by the MGI T7 platform used in the GSRD project. Therefore, we aimed to assess the applicability and robustness of the EnsembleTR pipeline on both Illumina and MGI SRS data through orthogonal approaches.

I. The Marshfield STR dataset

1. Refining genomic contexts of Marshfield STR loci

As capillary electrophoresis (CE) remained the state-of-the-art technology for STR genotyping, we utilized CE-based STR genotypes from the Marshfield Screening Sets as a gold standard dataset. To resolve the genomic contexts and repeat structures of 627 Marshfield STR loci, we adopted strategies similar to those described in the previous studies (*36*, *116*). The genomic annotation file for these STR loci, along with information about their PCR-amplified fragments, was curated by Pemberton et al. (*38*) and downloaded from https://rosenberglab.stanford.edu/data/pembertonEtAl2009/Pemberton_AdditionalFile1_11242009.txt. The PCR fragment analysis results for these loci across 1,048 HGDP-CEPH Human Genome Diversity Cell Line Panel were obtained from https://web.stanford.edu/group/rosenberglab/data/rosenbergEtAl2005/combinedmicrosats-1048.stru.

To redefine the genomic coordinates of these loci, we performed Nucleotide BLAST (https://blast.ncbi.nlm.nih.gov/Blast.cgi?PAGE_TYPE=BlastSearch) on their annotated reference sequences against the GRCh38 genome. For multiple alignment results, we only retained hits on the primary assemblies with the lowest e-values. Alignments with sequence identity below 95% or where the aligned length differed from the original annotated sequences by more than 7 bp (95th percentile of total alignments) were excluded, leaving 575 loci for further analysis. Subsequently, we ran Tandem Repeats Finder v4.09.1 (*117*) to scan the reference sequence of each locus following the guidelines provided at https://github.com/HipSTR-Tool/HipSTR-references for repeat structures. Loci with ambiguous repeat structures or those differing by more than 5 repeats from the published repeat number

were removed. Ultimately, 390 Marshfield STR loci with clearly defined repeat structures were kept for SRS WGS-based genotyping.

2. WGS-based genotyping

We next utilized HipSTR (*36*) and GangSTR (*37*) to genotype the Marshfield STR loci for HGDP samples analyzed in this study using the same parameters as described in the main text. After merging batch VCF files, we applied dumpSTR in TRTools v6.0.2 (*93*) with "--use-length --zip --drop-filtered --hipstr-min-call-Q 0.9" for HipSTR output and "--drop-filtered --gangstr-min-call-Q 0.9 --gangstr-filter-spanbound-only --gangstr-filter-badCI" for GangSTR output to filter out low-quality calls. Subsequently, we adopted EnsembleTR v1.0.0 (*35*) to integrate the results of the two genotypers. Calls with consensus score < 0.9 were removed from the EnsembleTR output using BCFTools v2.3.0 (*91*).

3. Comparison between CE- and WGS-based genotypes

We finally assessed the concordance between CE- and WGS-based STR genotypes for 825 samples shared between the two datasets. Following the strategy outlined in our previous work (*116*), we defined the absolute locus dosage change as $\mathrm{d} = |\mathrm{a1} - \mathrm{a2}|$, where a1 and a2 represent the basepair lengths of the two sister alleles at each locus, respectively. Given that CE results may also capture variations of InDels outside STR loci, we excluded WGS genotypes with InDels located more than two repeats away from STRs within the amplified regions. As a result, we observed high correlation between CE- and WGS-based STR genotypes, achieving concordance rates of 96.7%, 97.8%, and 97.6% for GangSTR (Pearson's $r = 0.942$, $P < 2.2 \times 10^{-16}$), HipSTR (Pearson's $r = 0.972$, $P < 2.2 \times 10^{-16}$), and EnsembleTR (Pearson's $r = 0.970$, $P < 2.2 \times 10^{-16}$) across over 250,000 genotypes (**fig. S3**). These findings indicate that the EnsembleTR pipeline demonstrates significant strengths in genotyping known STR loci that are comparable to CE-based methods.

II. STR genotyping for the Chinese Quartet

The GSRD cohort has utilized DNA samples from the Chinese Quartet (*39*) as reference materials to monitor potential technical issues during the long-term implementation of the sequencing program. Consequently, pedigree information among the parents (father: F7, mother: M8) and two monozygotic daughters (D5 and D6) in the Chinese Quartet provides an invaluable resource for scrutinizing the accuracy of genome-wide STR genotypes. In this study, we used WGS from the quartet samples sequenced in the same run to further validate the EnsembleTR pipeline for genotyping STRs in family samples. Sequencing, read processing, WGS data quality control, and genome-wide STR genotyping for the quartet samples were performed as per the procedures described in the main text. We analyzed Mendelian across the two parent-child trios in the quartet using BCFtools v2.3.0 (*91*) with the +Mendelian2 plugin. For both trios, we found that the EnsembleTR (*35*) pipeline consistently achieved the lowest Mendelian error rate among the three methods, being approximately ~50% and ~90% lower than HipSTR and GangSTR outputs, respectively (**fig. S4A**). Furthermore, we evaluated the genotype concordance between the two monozygotic twins. As a result, we observed 98.1%, 98.9%, and 99.6% monozygotic concordance for GangSTR, HipSTR, and EnsembleTR, respectively (**fig. S4B**). Collectively, these results demonstrate the high performance of the EnsembleTR pipeline for genome-wide STR calling in the established quartet family samples.

III. PacBio HiFi long-read STR genotypes

PacBio HiFi long-read sequencing (LRS) has emerged as a promising technology for comprehensively characterizing STR variations across the genome for its superior read length and quality compared to SRS technologies (*4*). Therefore, we utilized PacBio HiFi data from two GSRD samples as a high-quality truth set to further evaluate the accuracy of their SRS WGS-based STR genotypes. PacBio HiFi sequencing libraries were prepared following the manufacturer's best practices (https://www.pacb.com/wp-content/uploads/Procedure-checklist-Preparing-whole-genome-and-metagenome-libraries-using-SMRTbell-prep-kit-3.0.pdf), and each sample was sequenced to ~20× depth on a PacBio Revio sequencer. Raw HiFi reads were aligned to the GRCh38 reference genome using Minimap2 in "map-hifi" mode. Genome-wide STRs were called from the aligned PcaBio HiFi data using TRGT v3.0.0 (*86*) based on an STR catalog matched to SRS data. We filtered out TRGT calls with fewer than five spanning reads supporting each allele using BCFTools v2.3.0 (*91*). After that, the absolute locus dosage changes between the SRS and LRS datasets were compared as described in Section I. Overall, 92.7%, 94.9%, and 94.7% SRS-LRS genotype concordance were achieved for GangSTR, HipSTR, and EnsembleTR for total STRs, respectively. Although the overall performance of EnsembleTR was slightly lower than that of HipSTR alone, we observed the highest genotype concordance for STRs with reference alleles >25 bp (**fig. S5**). Thus, the EnsembleTR results represent an optimized balance for both short and long STRs. Additionally, we compared the performance of these SRS WGS-based methods for CDS and non-CDS STRs. As a result, all three methods showed higher performance for CDS STRs than non-CDS STRs (**fig. S5**), probably due to the relatively smaller number and simpler structures of STRs in CDS regions.

Taken together, these results demonstrate that the EnsembleTR pipeline is suitable for accurate genome-wide STR calling from SRS WGS data.

Supplementary Text 2

Evaluating covariates affecting STR discovery

The GSRD project aims to explore molecular mechanisms underlying rare diseases and identify genetic risks specific to Chinese populations using high coverage WGS data. Accordingly, our final dataset included both individuals affected by rare diseases and healthy controls. Given that our objective was to characterize population-specific STR variations and explore their functional roles within Tibeto-Burman-speaking populations, we did not differentiate between the disease statuses of individuals in order to maximize the statistical power of discovery. To the best of our knowledge, this approach is commonly adopted in current WGS cohort studies focusing on STRs, such as when investigating population patterns (*31*), regulatory functions (*87*), or GWAS/PheWAS analyses (*6*, *8*). We assume that this study design had minimal impact on population-level STR discovery for the following reasons: First, the sample sizes of affected and unaffected individuals were relatively balanced (1,827 affected patients and 1,981 healthy control individuals). Second, the affected individuals were diagnosed with a diverse range of diseases with heterogeneous genetic etiologies (**data S1**). Therefore, the likelihood that uncharacterized pathogenic STRs from affected individuals bias into the population-specific STR profiles is negligible.

To validate this assumption, we employed a generalized linear regression model (GLM) to identify potential covariates influencing STR discovery. Since pathogenic STR variants often

manifest as outlier expansions, we first fitted a model on the number of STR expansions per sample using the following formula:

$$N_{exp} \sim \beta_1 * G + \beta_2 * D + \beta_3 * A + \beta_4 * S + \beta_5 * A{:}D + \beta_6 * S{:}D + \varepsilon$$

Where N_{exp} represents the number of expanded STR alleles per sample (adjusted for sample missingness), G denotes the population groups in the GSRD dataset (HAN, TIB, YI, TUJ, and OTB), D indicates the disease status (affected / healthy), A is the recoded age, S is the sex of the sample, β_1 to β_6 are the regression coefficients, and ε is the error term. Considering that disease prevalence may vary by age and sex, we also included interaction terms for age:disease (A:D) and sex:disease (S:D). Our results revealed that the number of expanded alleles was significantly associated with population group, whereas disease status did not contribute to the GLM model (P = 0.097, **Table S1**).

Similarly, we applied the same model to assess the number of contraction alleles (**Table S2**), frameshift variants (**Table S3**), and total pLoF variants (**Table S4**) per sample. The results indicated that sample age, sex, and disease status had no influence on the detection of these variant types ($P \geq 0.05$).

Table S1. Summary statistics for the per-sample expansion alleles GLM model.

Term	Coefficient (β)	Standard Error	Statistic	*P*
(Intercept)	28.060	0.777	36.109	4.4×10^{-209}
Group (TIB)	3.535	0.530	6.669	3.5×10^{-11}
Group (YI)	-1.788	0.549	-3.258	0.001
Group (TUJ)	2.979	0.637	4.679	3.1×10^{-6}
Group (OTB)	1.441	1.050	1.373	0.170
Disease status	1.597	0.961	1.662	0.097
Age	-8.3×10^{-4}	0.017	-0.048	0.962
Sex	0.409	0.507	0.807	0.420
Age:Disease	-0.017	0.024	-0.728	0.466
Age:Disease	-0.013	0.678	-0.020	0.984

Table S2. Summary statistics for the per-sample contraction alleles GLM model.

Term	Coefficient (β)	Standard Error	Statistic	*P*
(Intercept)	4.018	0.207	19.439	1.3×10^{-75}
Group (TIB)	-0.864	0.142	-6.105	1.3×10^{-9}
Group (YI)	0.549	0.146	3.757	1.8×10^{-4}
Group (TUJ)	-0.222	0.169	-1.311	0.190
Group (OTB)	-0.769	0.279	-2.759	0.006
Disease status	0.272	0.256	1.062	0.288
Age	-0.002	0.005	-0.359	0.720
Sex	-0.053	0.135	-0.393	0.694
Age:Disease	0.006	0.006	1.003	0.316
Age:Disease	0.033	0.181	0.184	0.854

Table S3. Summary statistics for the per-sample frameshift variants GLM model.

Term	Coefficient (β)	Standard Error	Statistic	*P*
(Intercept)	17.744	0.307	57.798	0.000
Group (TIB)	0.818	0.209	3.904	9.9×10^{-5}
Group (YI)	-0.046	0.217	-0.211	0.833
Group (TUJ)	0.243	0.252	0.967	0.334
Group (OTB)	-0.577	0.415	-1.392	0.164
Disease status	-0.475	0.380	-1.252	0.211
Age	-0.008	0.007	-1.140	0.255
Sex	-0.144	0.200	-0.718	0.473
Age:Disease	0.015	0.009	1.579	0.114
Age:Disease	0.337	0.268	1.257	0.209

Table S4. Summary statistics for the per-sample pLoF variants GLM model.

Term	Coefficient (β)	Standard Error	Statistic	*P*
(Intercept)	77.484	0.757	102.392	0.000
Group (TIB)	2.523	0.516	4.888	1.1×10^{-6}
Group (YI)	1.022	0.535	1.911	0.056
Group (TUJ)	0.462	0.620	0.745	0.456
Group (OTB)	-0.449	1.023	-0.439	0.660
Disease status	0.033	0.936	0.035	0.972
Age	2.1×10^{-4}	0.017	0.012	0.990
Sex	-0.385	0.494	-0.779	0.436
Age:Disease	-0.015	0.023	-0.636	0.525
Age:Disease	0.342	0.660	0.518	0.605

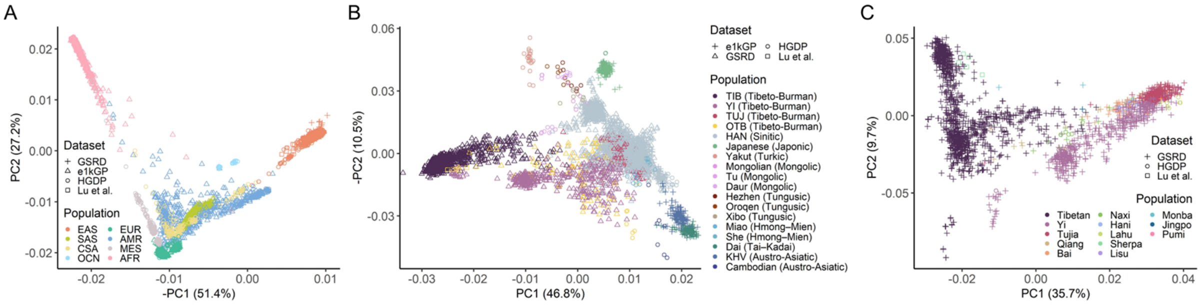

Fig. S1. PCA analysis on bi-allelic SNP genotypes of investigated populations. (**A**) Global populations. (**B**) East Asian (EAS) populations categorized by different language families. (**C**) TB-speaking populations only. The PCA results revealed that TB-speaking populations exhibited distinct population structures compared to other ethnolinguistically diverse groups. Notably, major TB-speaking populations such as Tibetan (TIB), Yi (YI), and Tujia (TUJ) displayed specific genetic structures. Other TB-speaking populations like Qiang, Bai, and Naxi exhibited and showed higher genetic affinity to the major TB-speaking populations. Consequently, these populations were classified into the Other TB speaker (OTB) group, representing a more complex TB-related ancestry background.

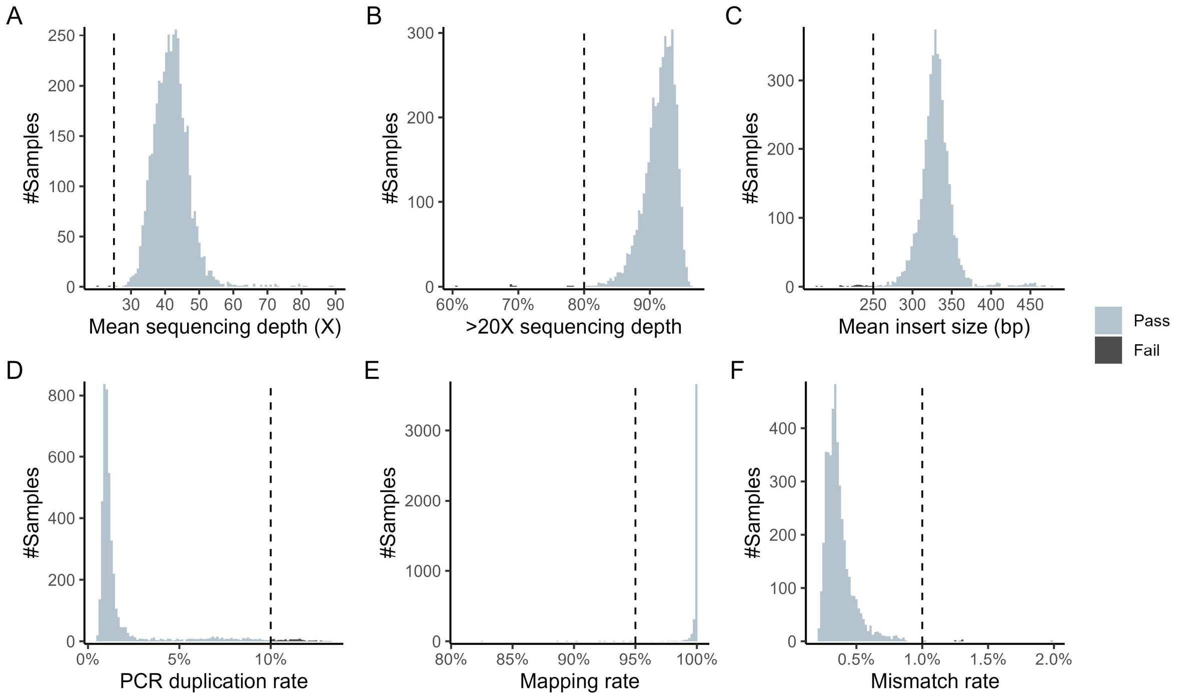

Fig. S2. Quality metrics of WGS data for newly-sequenced GSRD samples. (**A**) Mean sequencing depth per sample. (**B**) Proportion of the genome covered at >20× sequencing depth. (**C**) Mean fragment insert size. (**D**) PCR duplication rate. (**E**) Mapping rate of read bases. (**F**) Mismatch rate of read bases. The dashed lines indicate the filtering thresholds applied.

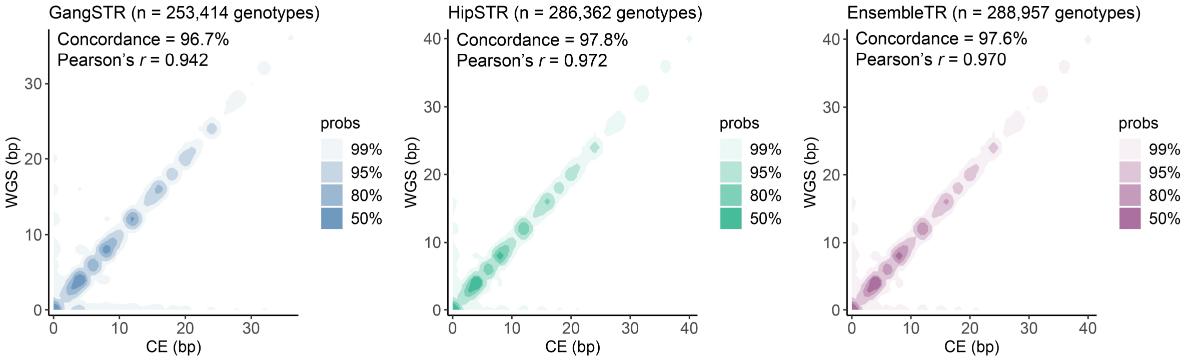

Fig. S3. Concordance of locus dosage changes between CE- and WGS-based genotypes for 390 Marshfield STRs. The absolute locus dosage change was defined as $\boldsymbol{d = |a1 - a2|}$, where a1 and a2 represent the basepair lengths of the two sister alleles at each locus, respectively.

A

Trio	Total site	Reference	Good	Missing	MERR	MI	Tool
D5-F7-M8	1,038,204	892,702	120,283	22,651	2,568	99.7%	EnsembleTR
D5-F7-M8	1,030,724	829,771	177,825	17,465	5,663	99.4%	HipSTR
D5-F7-M8	1,085,862	897,899	148,704	20,187	19,072	98.2%	GangSTR
D6-F7-M8	1,038,200	892,702	120,814	22,243	2,441	99.8%	EnsembleTR
D6-F7-M8	1,030,736	829,771	178,707	17,052	5,206	99.5%	HipSTR
D6-F7-M8	1,086,493	897,899	152,704	13,907	21,983	98.0%	GangSTR

B

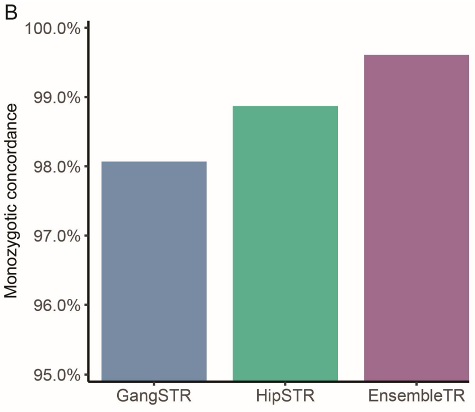

Fig. S4. Performance evaluation of WGS-based STR genotyping methods on the Chinese Quartet samples. (**A**) Genome-wide Mendelian inheritance error estimated from two parent-child trios. MERR: count of Mendelian error events; MI: Mendelian inheritance rate ($\boldsymbol{MI = MERR/(Total\ sites - Missing\ sites)}$). (**B**) Genotype concordance rate between two monozygotic twins by different genotyping methods.

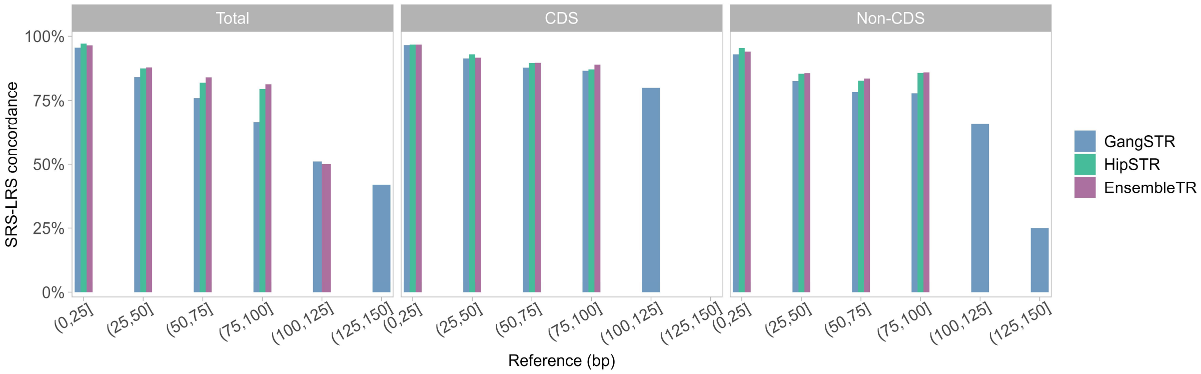

Fig. S5. Genome-wide concordance between SRS and LRS WGS-based genotypes for two GSRD samples, stratified by STR reference allele lengths and genic regions. Genotype concordance was quantified by the absolute locus dosage changes.

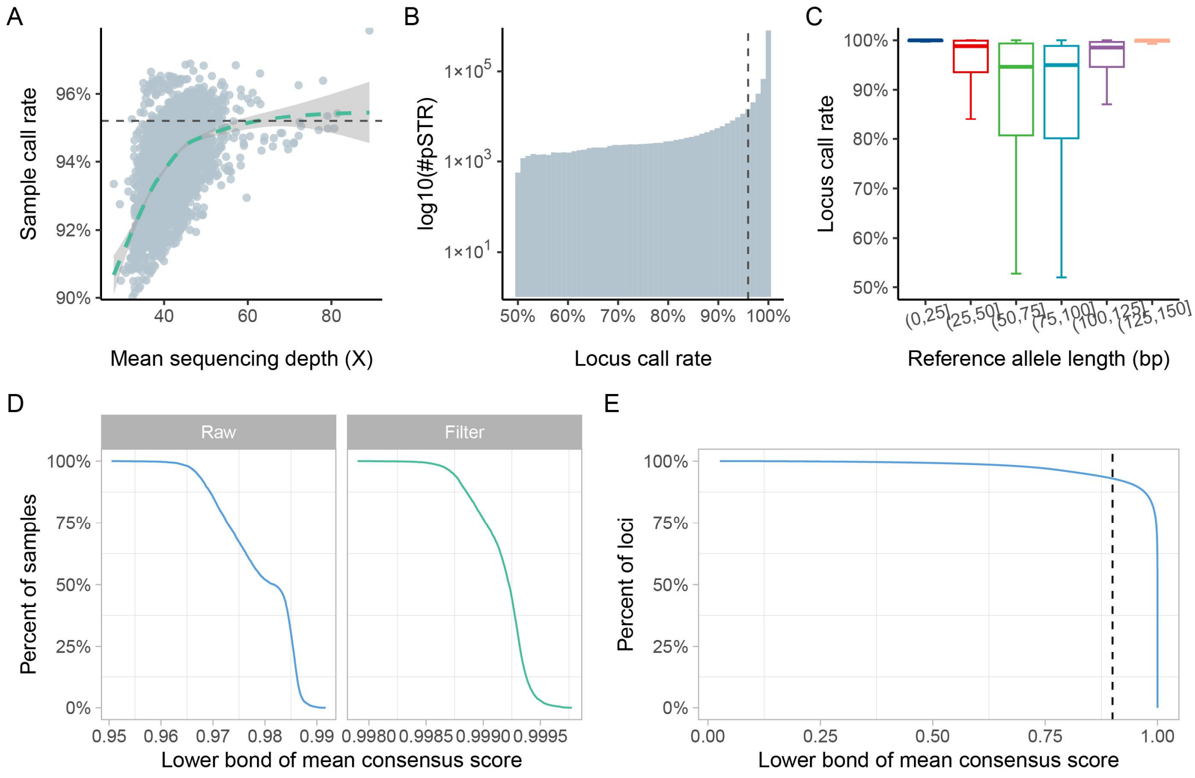

Fig. S6. Quality control of STR call set. (**A**) Correlation between mean sequencing depth and STR call rate for each sample. The black dashed line indicates the mean call rate across all samples. (**B**) Distribution of call rates per locus. The black dashed line indicates the mean call rate across all loci. (**C**) Call rate of loci stratified by reference allele lengths. (**D**) and (**E**) Cumulative distribution of mean genotype consensus scores across all loci for each sample (**D**) and across all calls for each STR locus (**E**). The black dashed line indicates the call-level filtering threshold of 0.9.

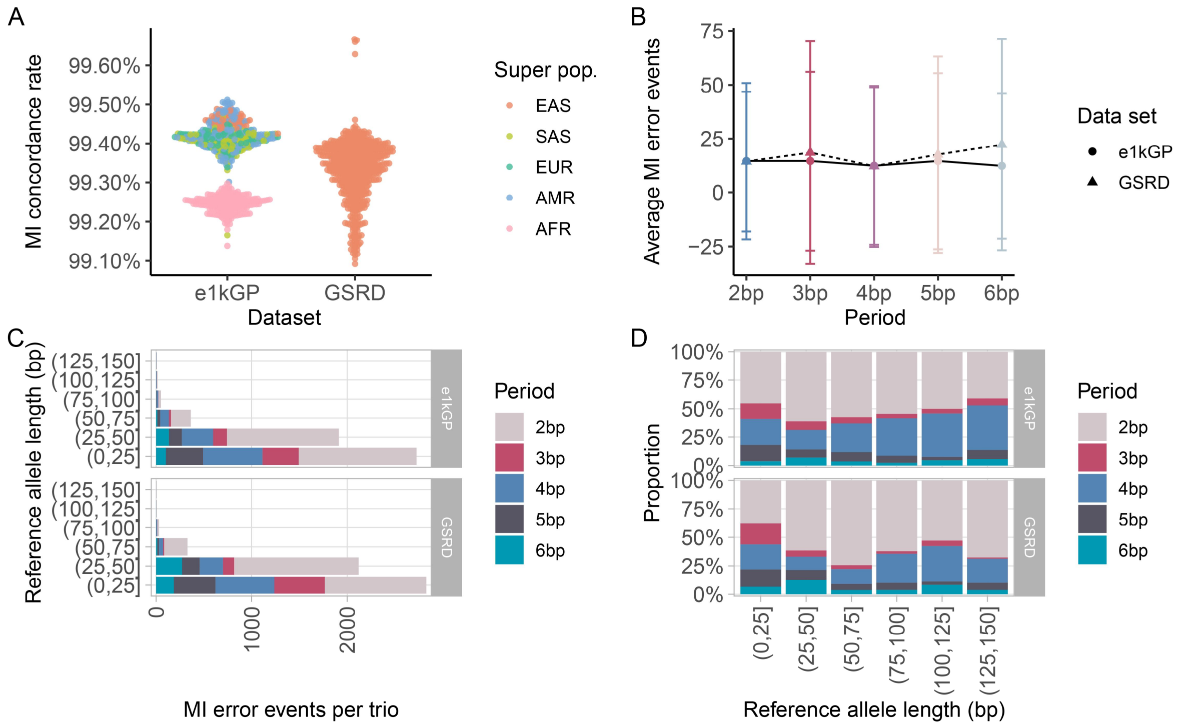

Fig. S7. MI patterns of pSTRs in parent-child trios. (**A**) MI consistency rate for trios from different continental populations. (**B**) Mean number of MI error events for STRs with different motif lengths. Error bars represent standard deviation. (**C**) MI error events per trio. (**D**) Composition of STRs with MI error events.

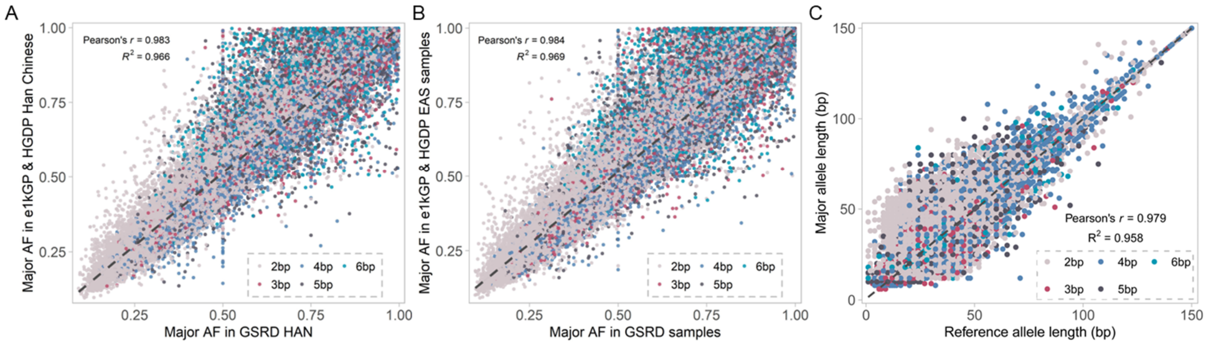

Fig. S8. Analysis of STR call set accuracy based on major alleles of pSTRs. (**A**) Correlation of pSTR major allele frequencies between Han Chinese (HAN) samples in the GSRD project and those in the e1kGP and HGDP projects. (**B**) Correlation of pSTR major allele frequencies between GSRD samples and East Asian samples in the e1kGP and HGDP projects. Comparisons were performed using population groups downsampled to the same size. (**C**) Correlation between reference and major allele lengths of pSTRs. The black dashed line indicates the gamma-smoothed trend.

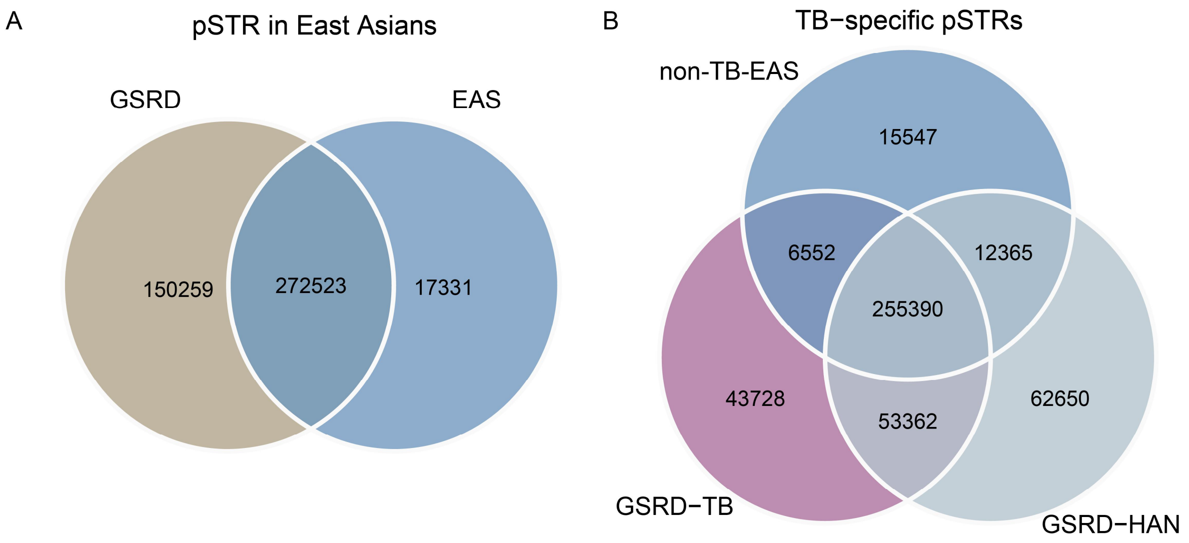

Fig. S9. Intersection of pSTRs among different sample sets. (**A**) Overlapping and distinct pSTRs between East Asian samples from the e1kGP and HGDP projects and GSRD samples. (**B**) Overlapping and distinct pSTRs among TB-speaking samples in GSRD (GSRD-TB), Han Chinese samples in GSRD (GSRD-HAN), and non-TB-speaking East Asian samples from the e1kGP and HGDP projects (non-TB-EAS).

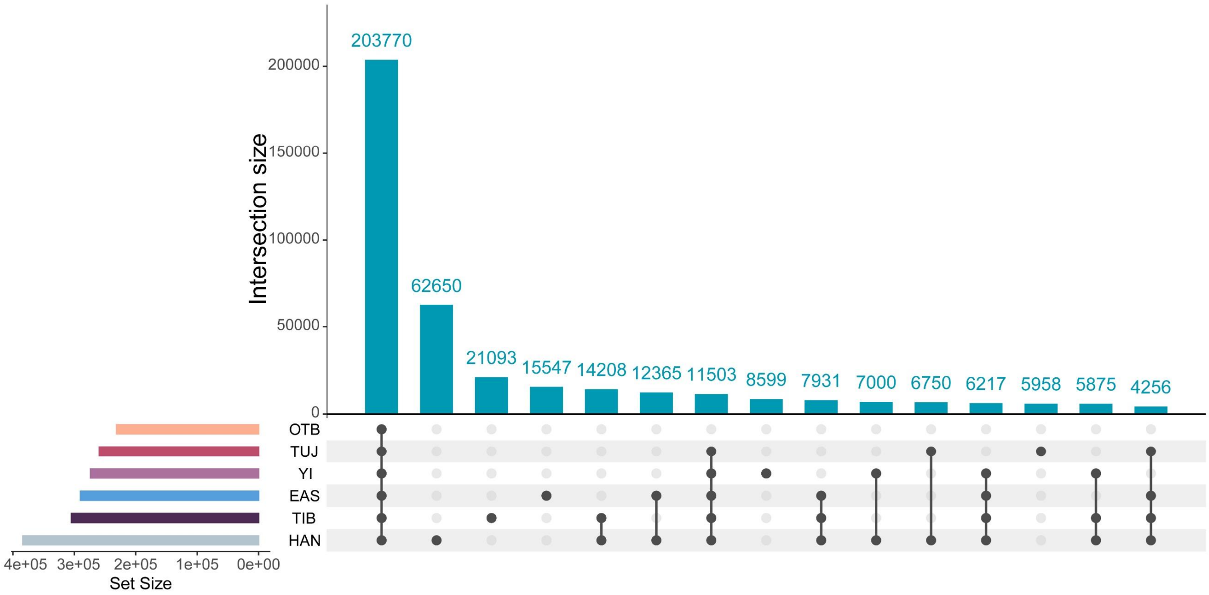

Fig. S10. Shared and unique pSTRs among investigated East Asian population groups, illustrated by an UpSet plot.

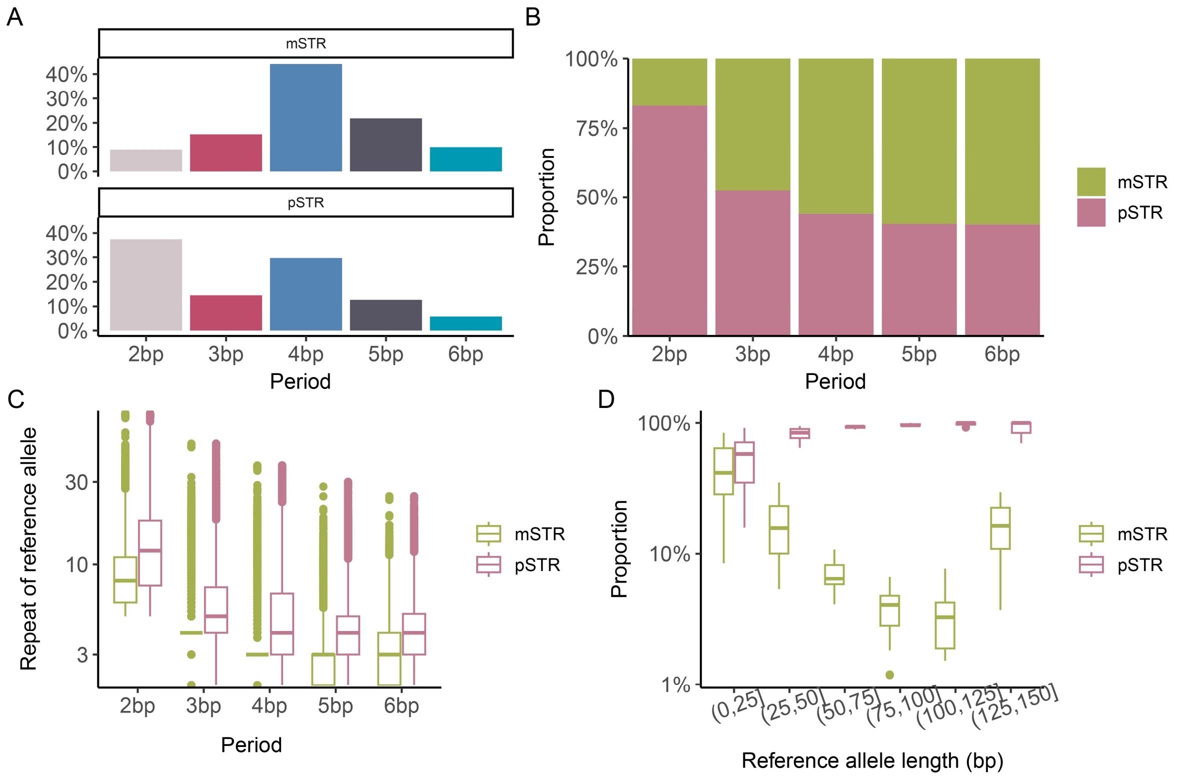

Fig. S11. Comparison of mSTR and pSTR in the call set. (**A**) Proportion of different motif types for mSTRs and pSTRs. Most pSTRs were di- and tetra-nucleotide repeats, while tetra-nucleotide repeats were preferentially observed as mSTRs. (**B**) Polymorphism status of STRs with varying motif lengths. STRs with longer motifs were less likely to be polymorphic. (**C**) Differences in repeat numbers of reference alleles between mSTRs and pSTRs. Reference alleles of mSTRs generally had fewer repeats. (**D**) Polymorphism status of STRs with varying reference allele lengths. A higher proportion of pSTRs and a lower proportion of mSTRs were observed among longer STRs.

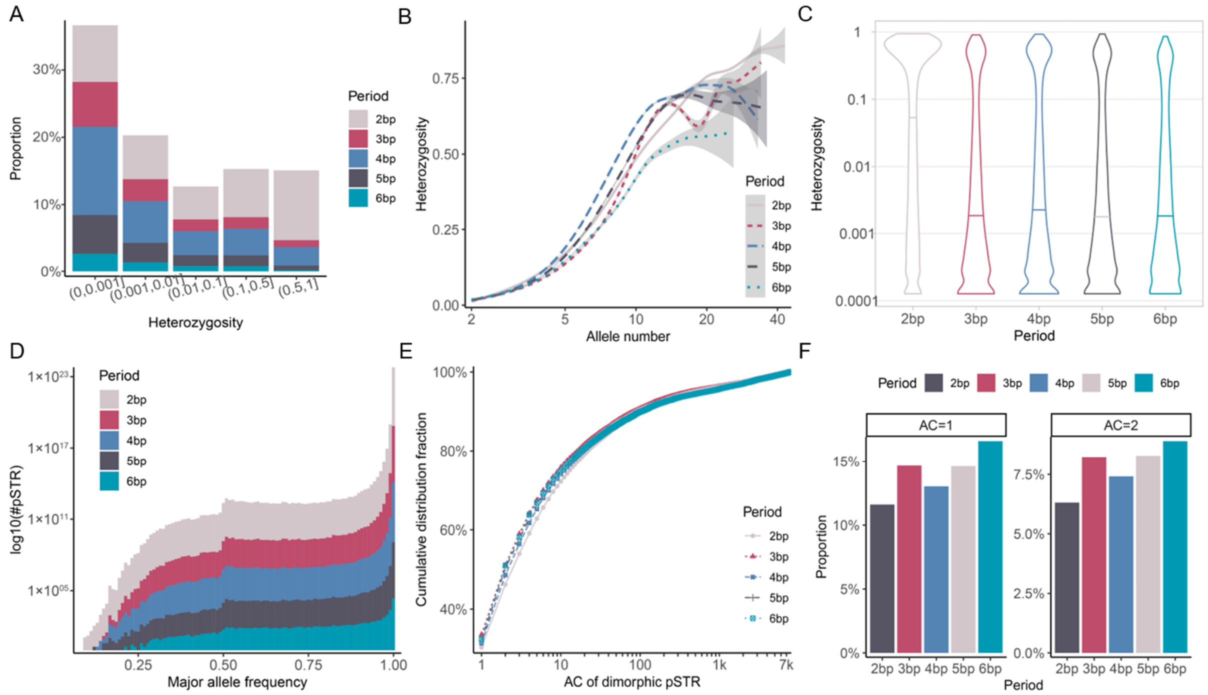

Fig. S12. Variability of pSTRs. (**A**) Distribution of heterozygosity of pSTR loci. (**B**) Correlation between heterozygosity and the number of alleles of pSTR loci. (**C**) Heterozygosity of pSTRs stratified by motif length. (**D**) Spectrum of major allele frequencies across pSTR loci. (**E**) Cumulative distribution of alternative allele counts (AC) for dimorphic pSTRs. (**F**) Proportion of alleles with AC = 1 and AC = 2 across all pSTR loci.

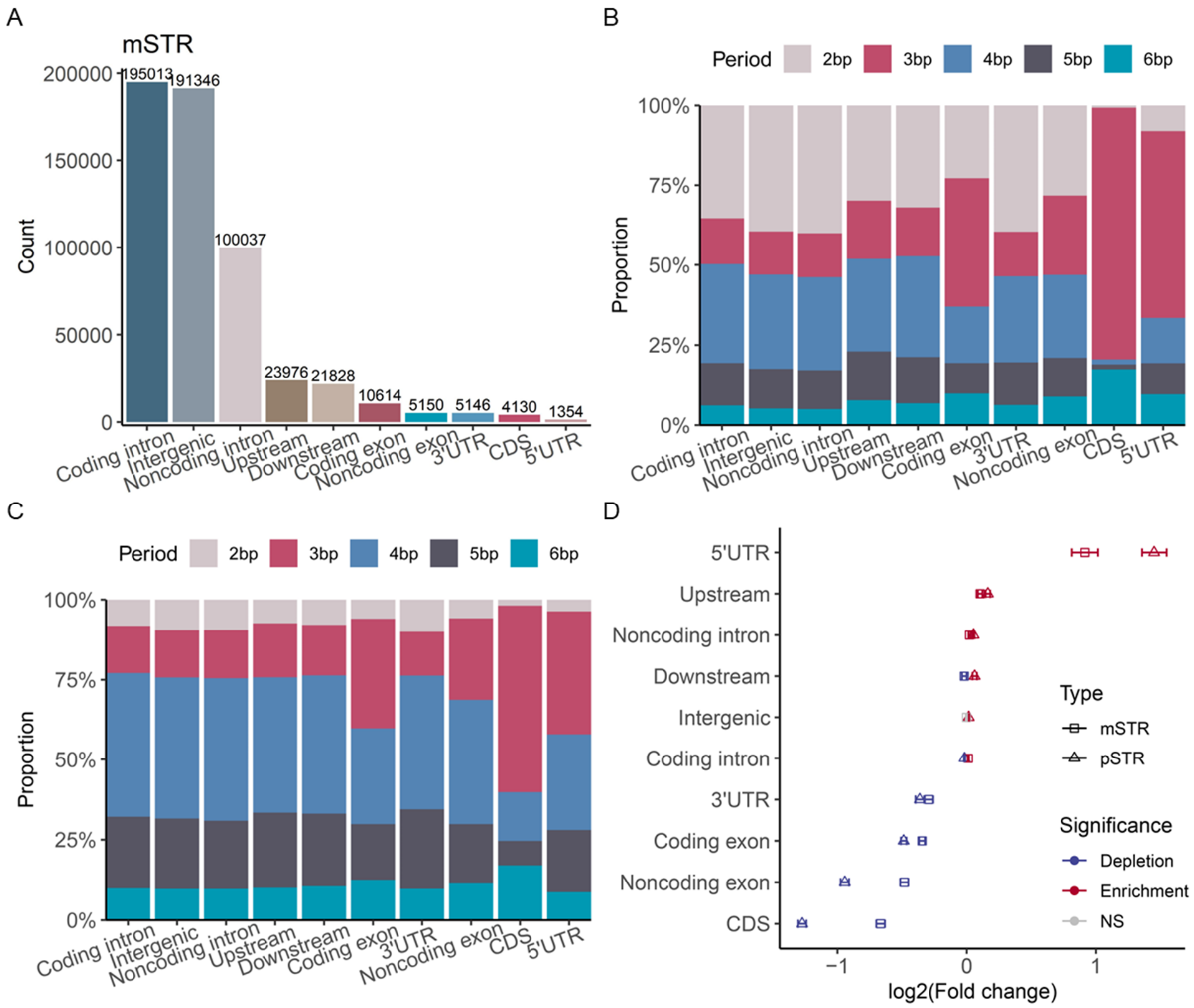

Fig. S13. Genomic contexts and characteristics of pSTRs and mSTRs. (**A**) Distribution of mSTR loci in designated genomic regions. (**B**) and (**C**) Proportion of pSTRs (**B**) and mSTR (**C**) loci in different genomic regions. (**D**) Enrichment of pSTRs and mSTRs in designated genomic regions. Empirical *P* values were calculated based on 2,000 permutations and adjusted using the Benjamini-Hochberg method. Blue and red dots indicate significant depletion and enrichment (adjusted $P < 0.05$), respectively. NS, not significant (adjusted $P \geq 0.05$).

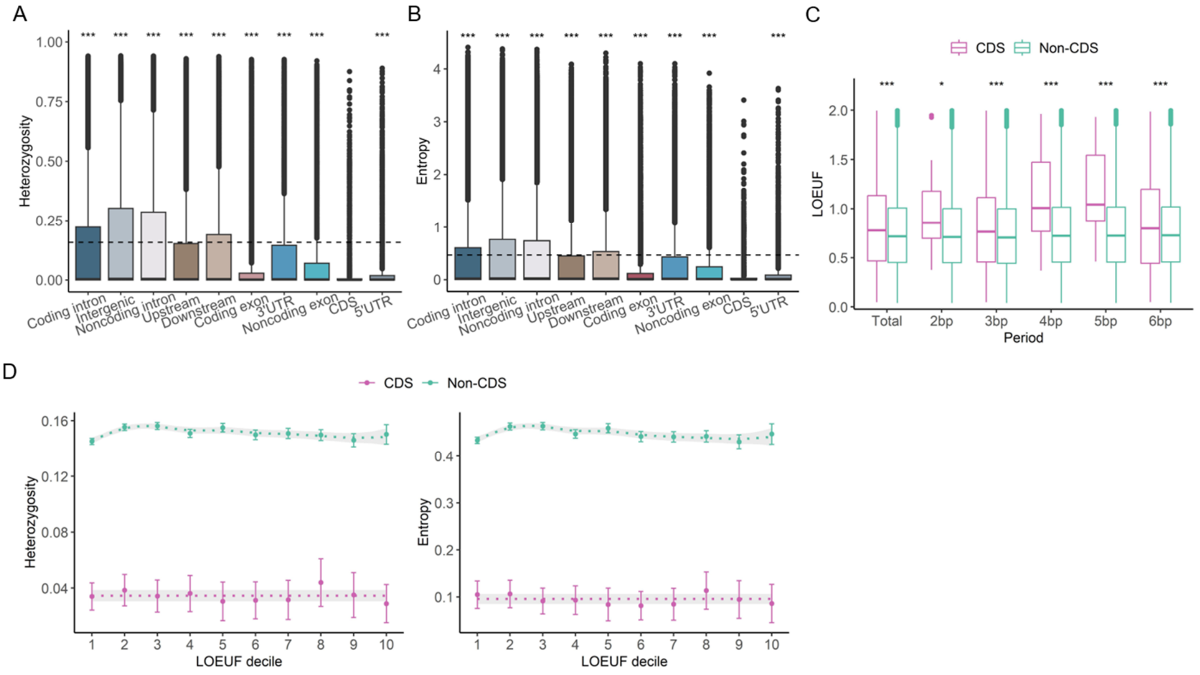

Fig. S14. Properties of CDS pSTRs. (**A**) and (**B**) Distribution of heterozygosity (**A**) and entropy (**B**) of pSTRs in different genomic regions. Two-sided Wilcoxon tests were performed with CDS pSTRs as the reference. Significance level: ***, $P < 0.001$. (**C**) Distribution of LOEUF scores for genes containing CDS and non-CDS pSTRs. (**D**) Mean heterozygosity and entropy for CDS and non-CDS pSTRs in genes across different LOEUF deciles. Error bars represent 95% confidence intervals.

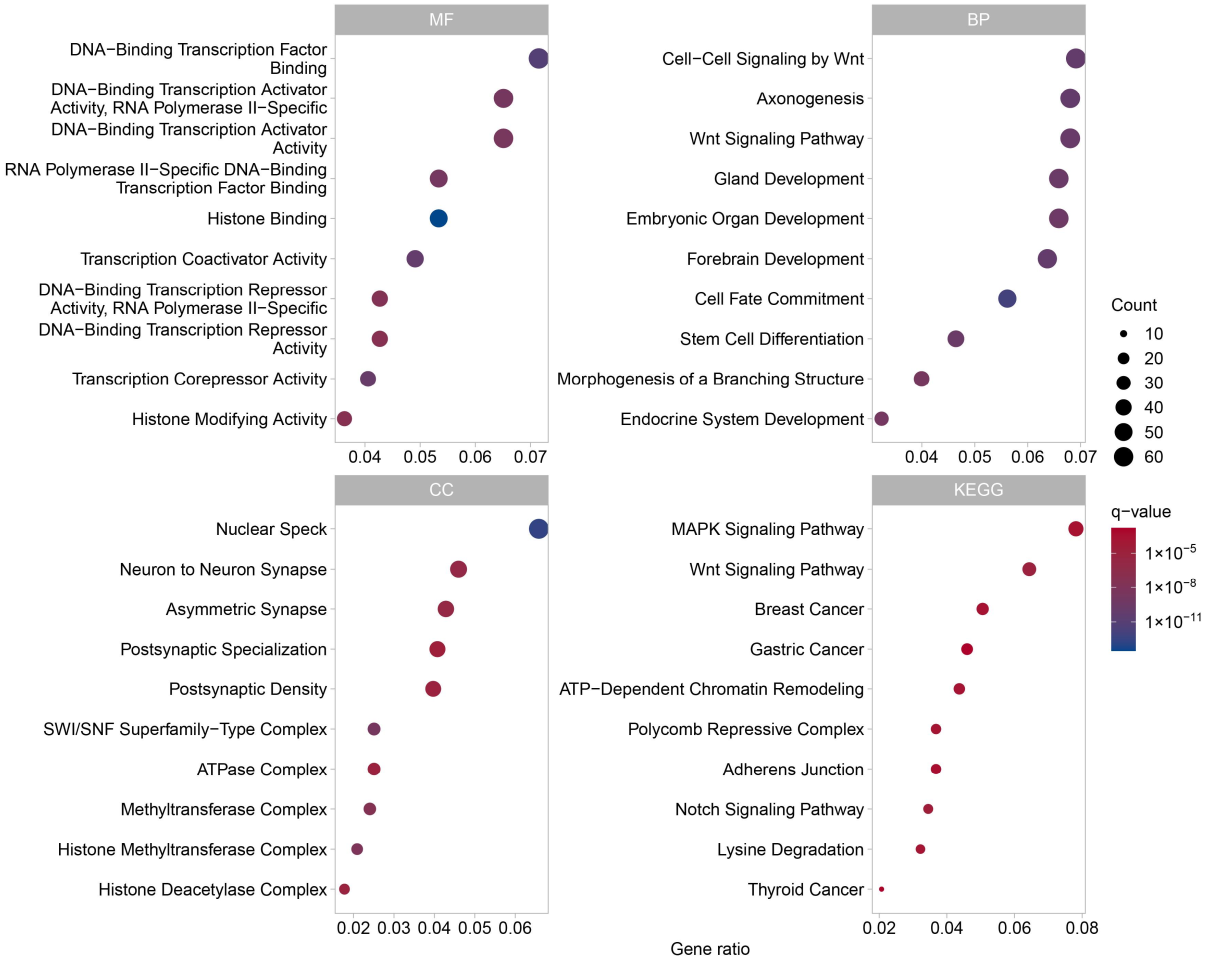

Fig. S15. Gene Ontology (GO) and Kyoto Encyclopedia of Genes and Genomes (KEGG) enrichment analysis for genes in the first three LOEUF deciles containing CDS pSTRs by clusterProfiler v4.12.6. GO sub-ontologies include molecular function (MF), biological process (BP), and cellular component (CC).

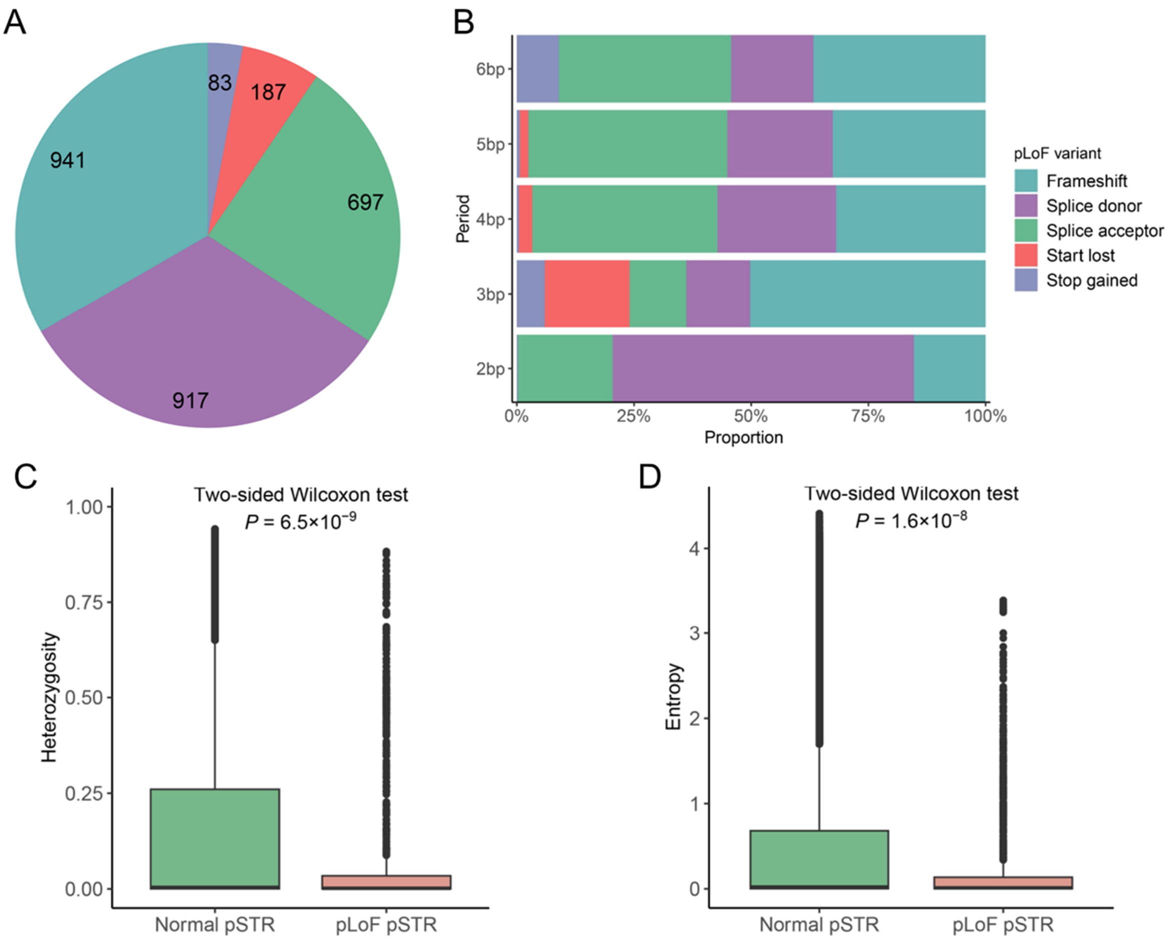

Fig. S16. Predicted loss-of-function (pLoF) pSTR variants. (**A**) and (**B**) Count (**A**) and proportion (**B**) of pLoF variants in different categories. (**C**) and (**D**) Distribution of heterozygosity (**C**) and sequence entropy (**D**) for normal pSTRs (with no pLoF variants) and pLoF pSTRs (with at least one pLoF variant).

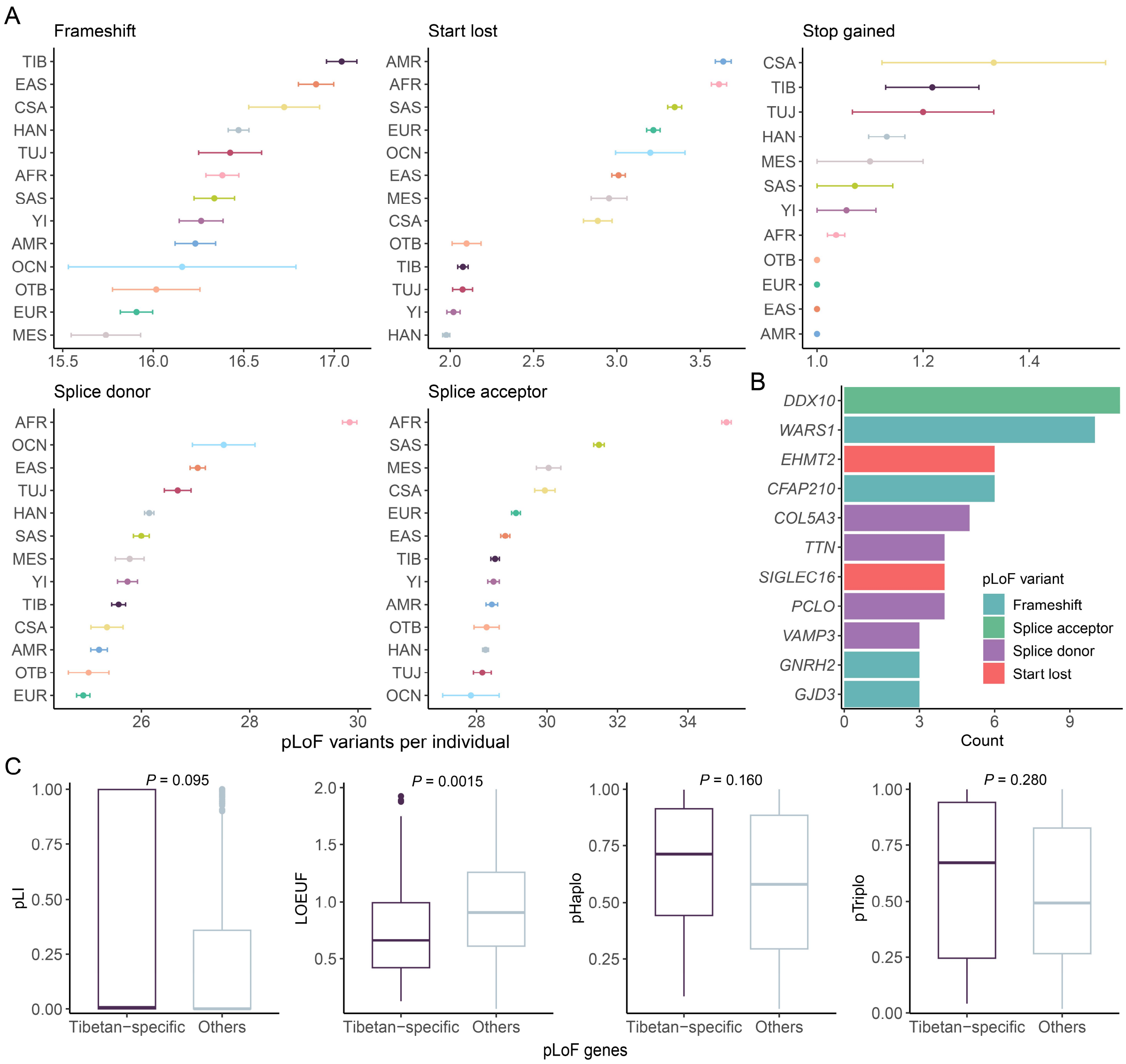

Fig. S17. pLoF burden in individual genomes. (**A**) Mean count pLoF variants per individual in different population groups. Error bars represent 95% confidence intervals. (**B**) Top genes influenced by Tibetan-specific pLoF variants. (**C**) Genomic constraint metrics of genes influenced by Tibetan-specific or other pLoF variants. A two-sided Wilcoxon test was performed for the between-group comparison.

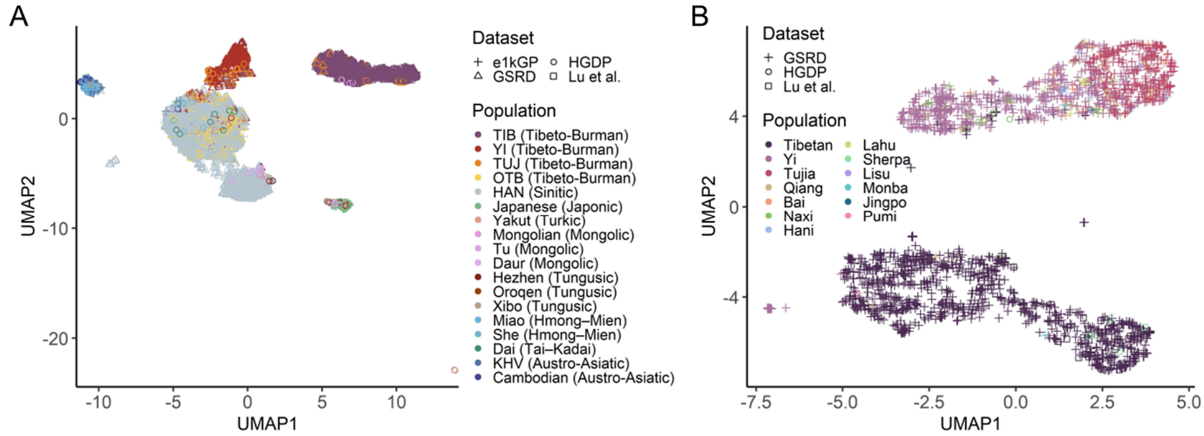

Fig. S18. Population structures revealed by UMAP analysis of hypervariable pSTRs (heterozygosity > 0.1). The analysis was performed using the top five principal components of pSTR variation for East Asian populations labeled by language families (**A**), and for TB-speaking populations only (**B**).

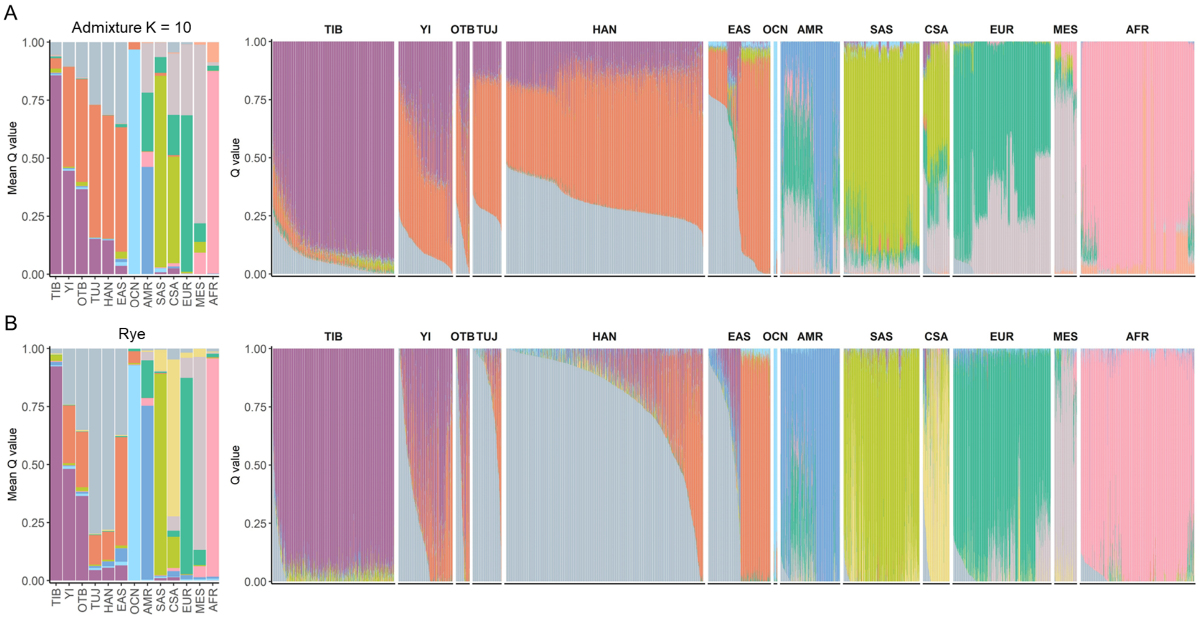

Fig. S19. Ancestry analysis of global populations. (**A**) Model-based ADMIXTURE analysis using bi-allelic SNPs. The optimal number of clusters K = 10 was determined based on cross-validation error estimation. (**B**) Genetic ancestry inferred from the top 10 principal components of hypervariable pSTRs (heterozygosity > 0.1) using the Rye package**.**

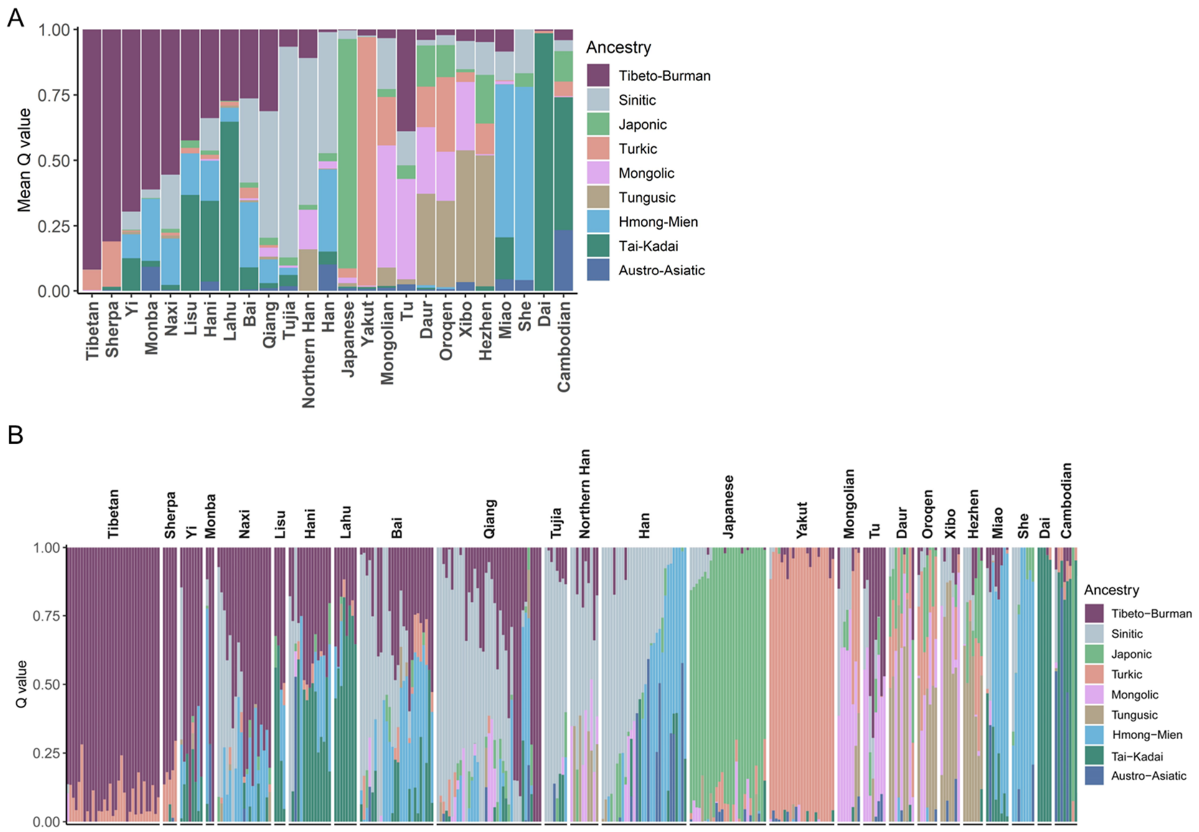

Fig. S20. Admixture patterns of ethnolinguistically distinct ancestry components (Q) among diverse East Asian populations inferred from the top 10 principal components of hypervariable pSTR variation (heterozygosity > 0.1). (**A**) Mean ancestry component (Q values) for different populations. (**B**) Individual-level ancestry compositions. For presentation purposes, this analysis included only publicly available Tibetan, Sherpa, Yi, and Tujia samples and excluded populations with fewer than three samples in the GSRD dataset (i.e., Pumi and Jingpo).

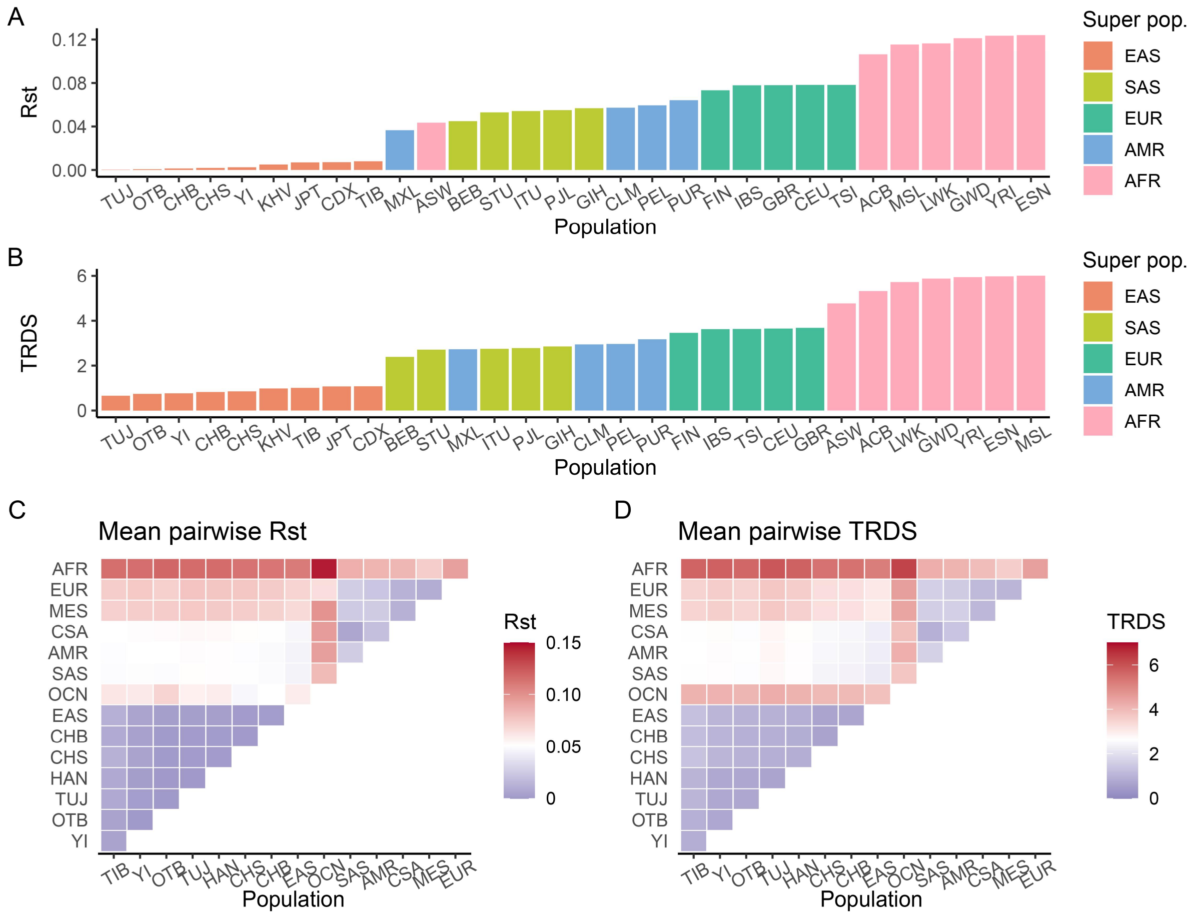

Fig. S21. Genetic distances between paired populations based on pSTR variation. (**A**) and (**B**) Mean pairwise Rst (**A**) and TRDS (**B**) values between TB-speaking populations and worldwide reference populations. CHB and CHS represent Han Chinese in Beijing and South China in e1kGP, respectively; EAS represents the remaining East Asian populations in e1kGP. (**C**) and (**D**) Mean Rst (**C**) and TRDS (**D**) values relative to the Han Chinese in GSRD (GSRD-HAN). To minimize the influence of unbalanced sample sizes, the TIB, YI, TUJ, and GSRD-HAN groups were down-sampled to 100 individuals for Rst and TRDS calculations.

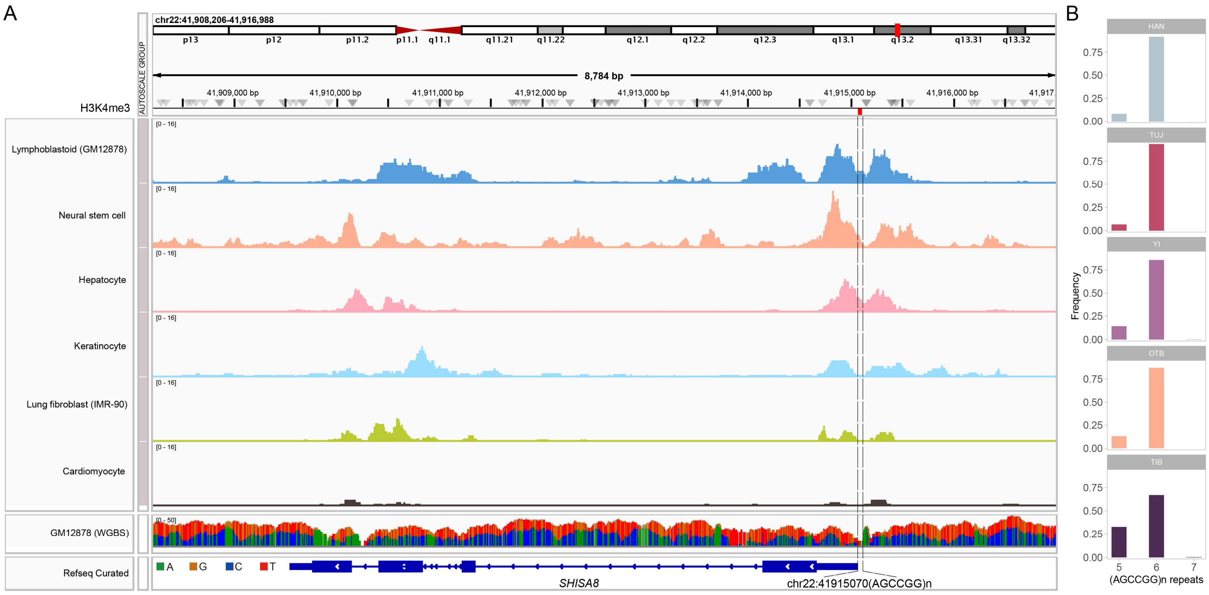

Fig. S22. Genomic and epigenetic contexts of the TB-divergent chr22:41915070(AGCCGG)n locus. (**A**) H3K4me3 histone mark peaks in regions flanking the target STR in six cell lines. (**B**) Frequency distribution of the (AGCCGG)n repeats in five investigated Chinese population groups.

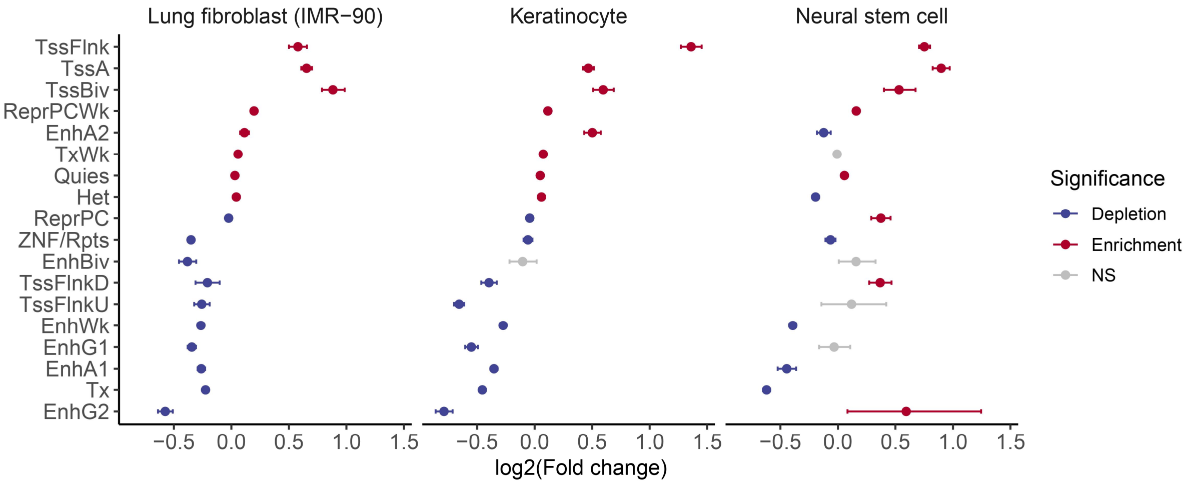

Fig. S23. Enrichment of pSTRs in chromatin states defined by ChromHMM in lung fibroblast, keratinocyte, and neural stem cell lines. Empirical *P*-values were calculated based on 2,000 permutations and adjusted using the Benjamini-Hochberg method. Red and blue dots indicate significant enrichment and depletion (adjusted $P < 0.05$), respectively. NS, not significant (adjusted $P \geq 0.05$). Error bars denote 95% confidence intervals.

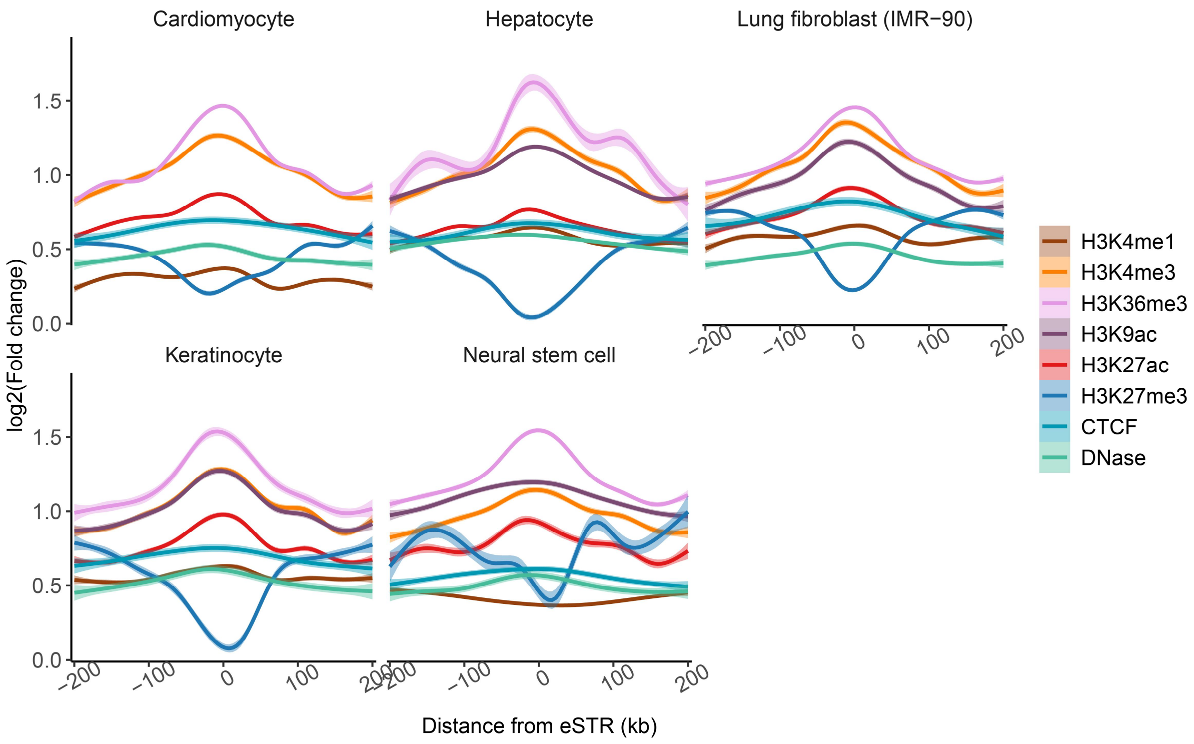

Fig. S24. Enrichment of epigenetic marks within 200 kb of eSTR loci compared to randomly sampled pSTR controls in five cell lines. Fold changes of enrichment were calculated based on 2,000 permutations.

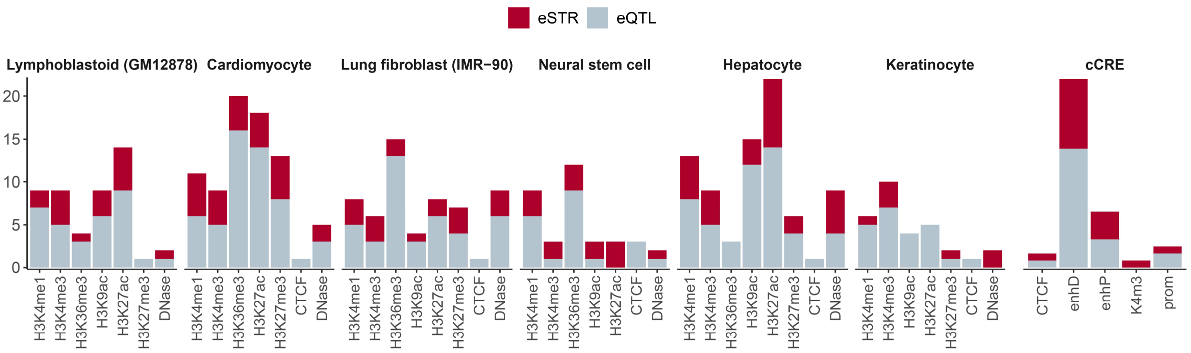

Fig. S25. Count of epigenetic marks and cCRE elements in different cell lines overlapping with TB-divergent pSTRs associated with gene expression. TB-divergent pSTRs were defined as those with an Rst > 0.1 between any TB-speaking populations and the Han Chinese (the GSRD-HAN group).

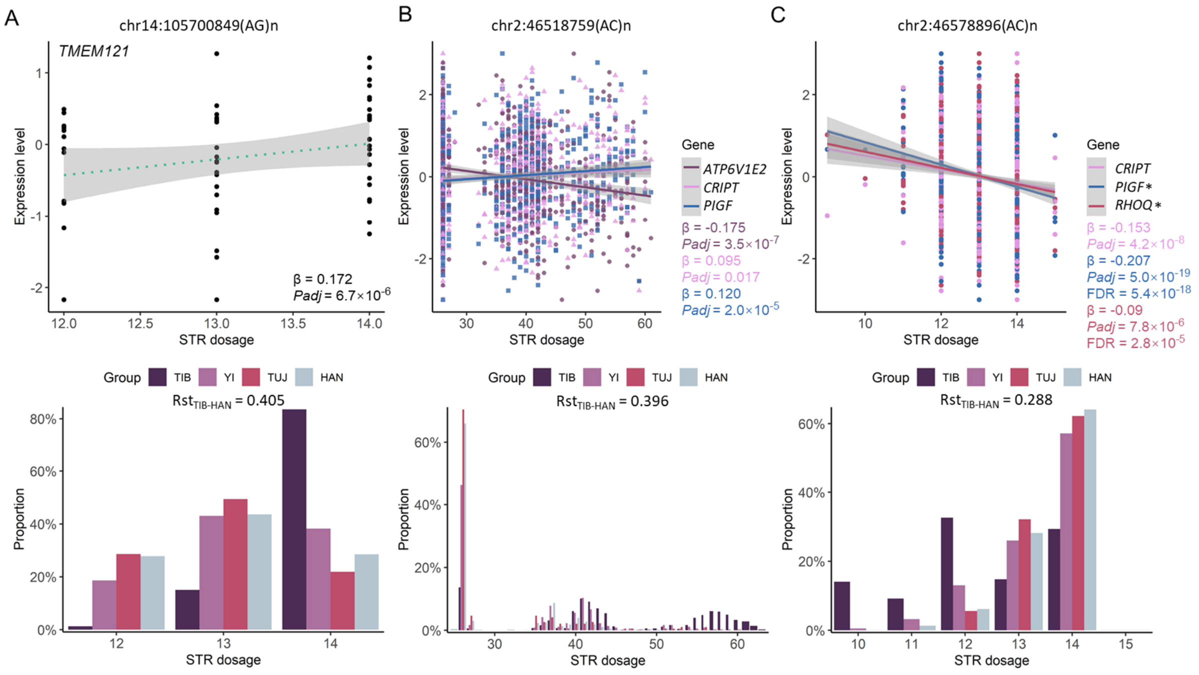

Fig. S26. Examples of highly divergent pSTRs (Rst > 0.2) between the Tibetans and Han Chinese. Top: Correlation between STR dosage and gene expression. Bottom: Distribution of STR dosage among the TIB, YI, TUJ, and HAN groups. *, the locus was identified as an eSTR for the *PIGF* and *RHOQ* genes.

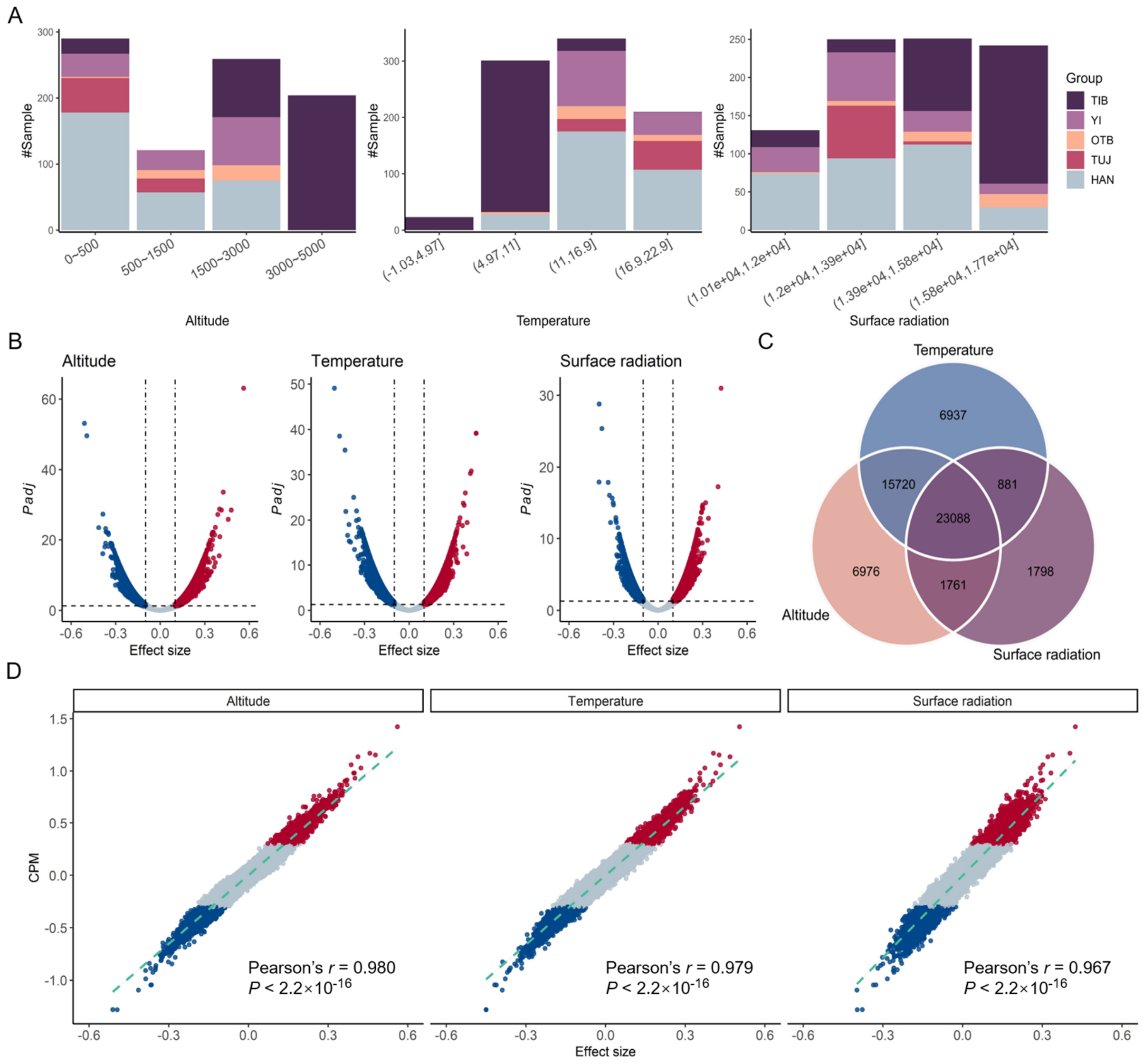

Fig. S27. Discovery of highland environments associated STRs (hSTRs). (**A**) Value ranges of environmental conditions for samples used in the STR dosage and environment association analysis. Units: altitude (meters above sea level), temperature (°C), solar radiation (kJ/m²·day). (**B**) Distribution of effect sizes from independent association tests. (**C**) Intersection of pSTRs significantly associated with different environmental conditions. (**D**) Correlation between CPM values and original effect sizes of environmental conditions for each pSTR locus. Red and blue dots indicate significant hSTRs (adjusted Fisher's $P < 0.05$, |CPM| > 0.3) with positive (red) and negative (blue) effects, respectively.

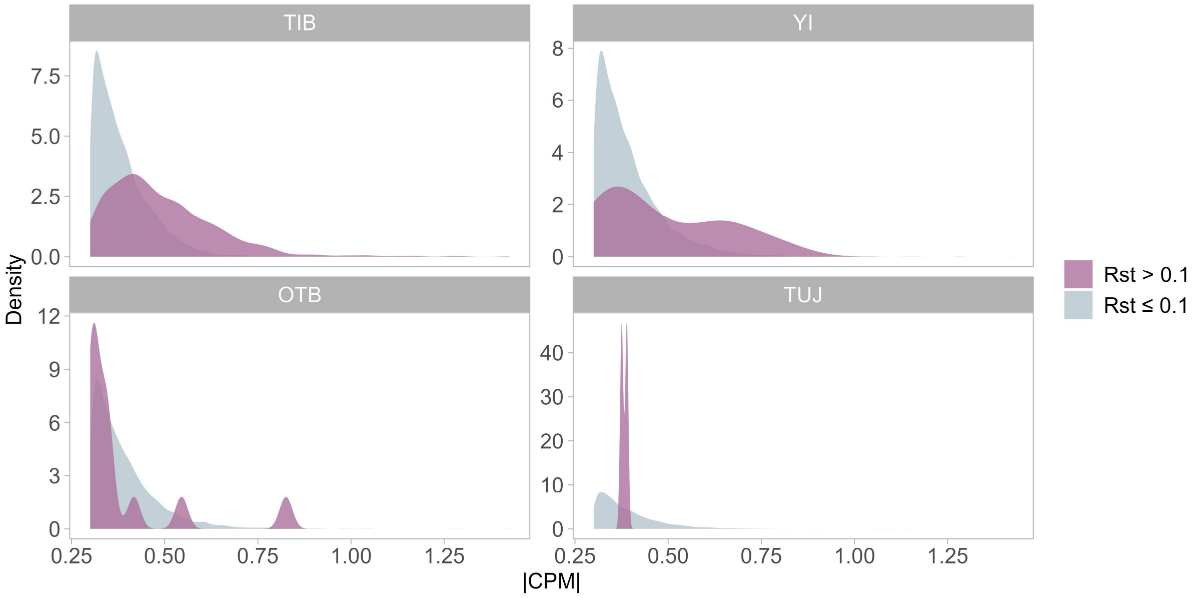

Fig. S28. Distribution of CPM values for TB-divergent (Rst > 0.1) and non-TB-divergent hSTRs (Rst ≤ 0.1). Rst values were calculated conditioning on the GSRD-HAN group.

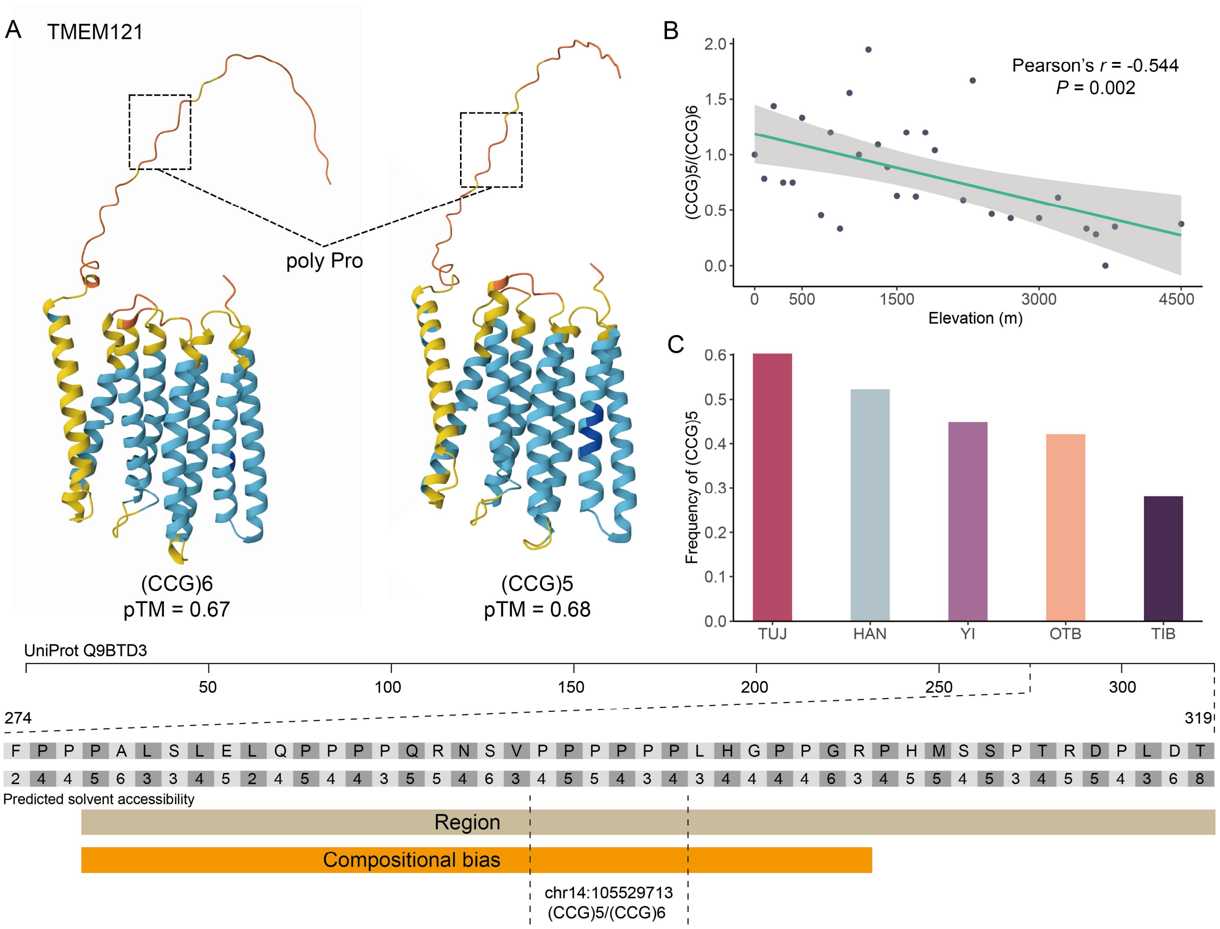

Fig. S29. Impact of the protein coding (CCG)n repeats on TMEM121 isoforms. (**A**) Predicted protein structures of (CCG)6- and (CCG)5-coding TMEM121 isoforms by AlphaFold3. (**B**) Correlation between (CCG)5/(CCG)6 allele proportion and elevation of the sampling site. (**C**) Frequency of the (CCG)5 repeats among different Chinese population groups.

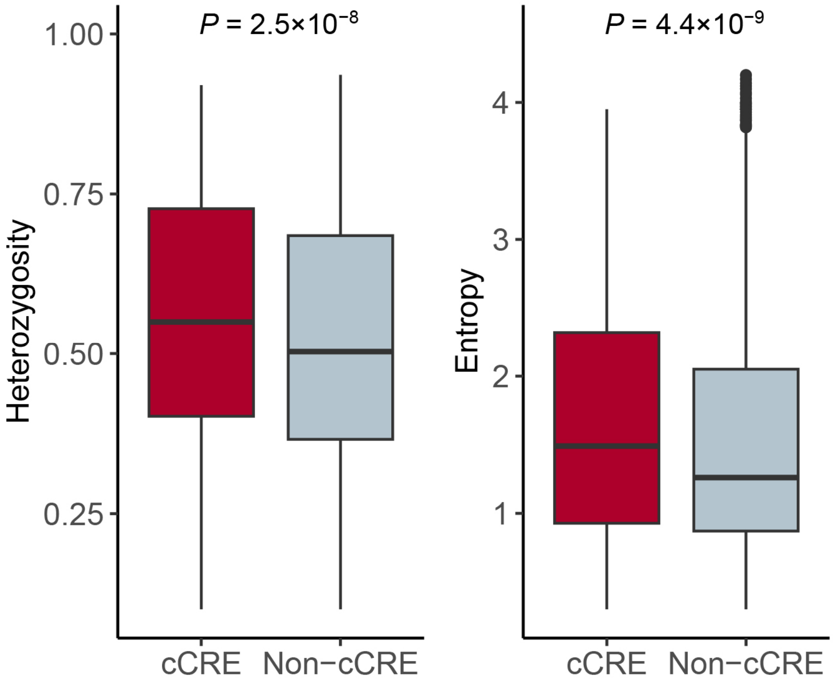

Fig. S30. Heterozygosity and sequence entropy of hSTRs overlapping with cCREs. The two-sided Wilcoxon test was performed to evaluate between-group differences.

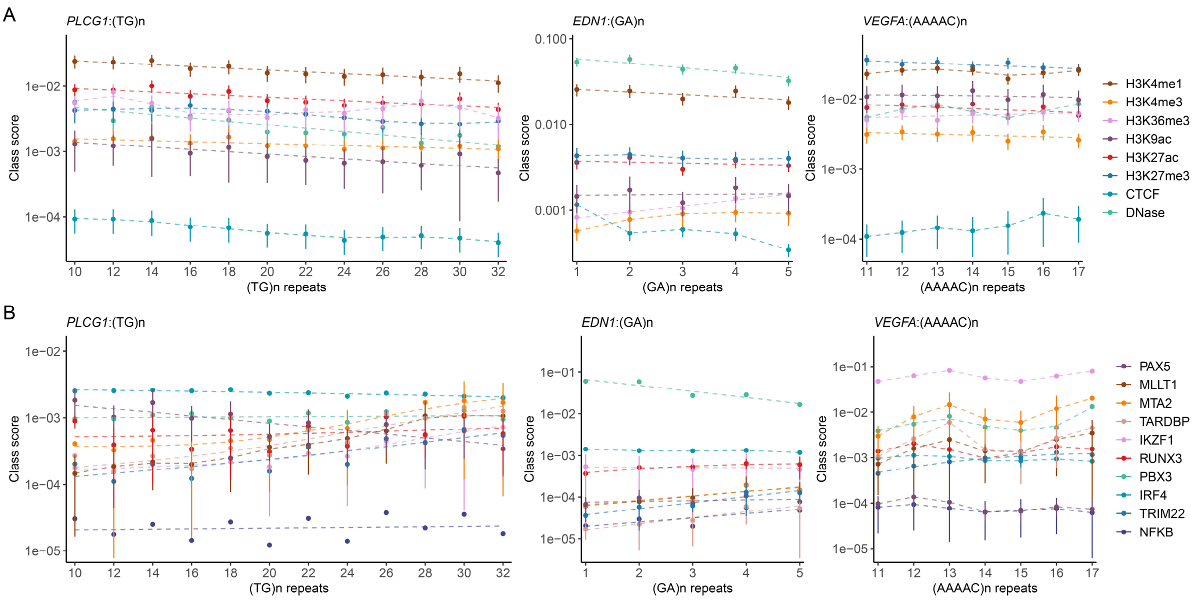

Fig. S31. Predicted effects of hSTRs on the epigenetic contexts of local sequences via the Sei model for *PLCG1*, *EDN1*, and *VEGFA* genes in the HIF pathway. (**A**) Epigenetic marks; (**B**) TF-binding affinities.

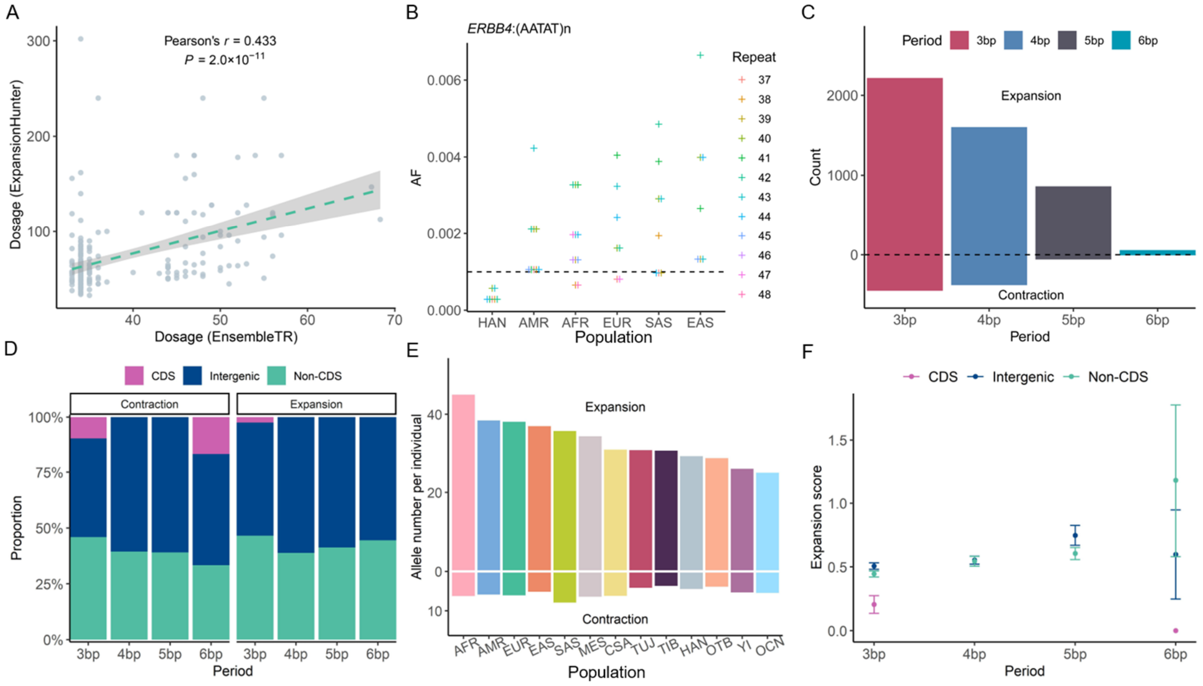

Fig. S32. Characterization of STR contractions and expansions. (**A**) Correlation between dosages of expanded loci characterized by the EnsembleTR pipeline and ExpansionHunter. (**B**) Frequency of alleles expanded at chr2:212192150(AATAT)n in the *ERBB4* gene across different populations. The dashed line denotes an allele frequency (AF) of 0.001. (**C**) Count of contracted and expanded alleles stratified by motif length. (**D**) Distribution of expanded and contracted alleles in specified genomic regions. (**E**) Number of expanded alleles per individual genome across different populations. (**F**) Mean expansion score of pSTRs with allele expansion or contraction in designated genomic regions. Error bars represent 95% confidence intervals.

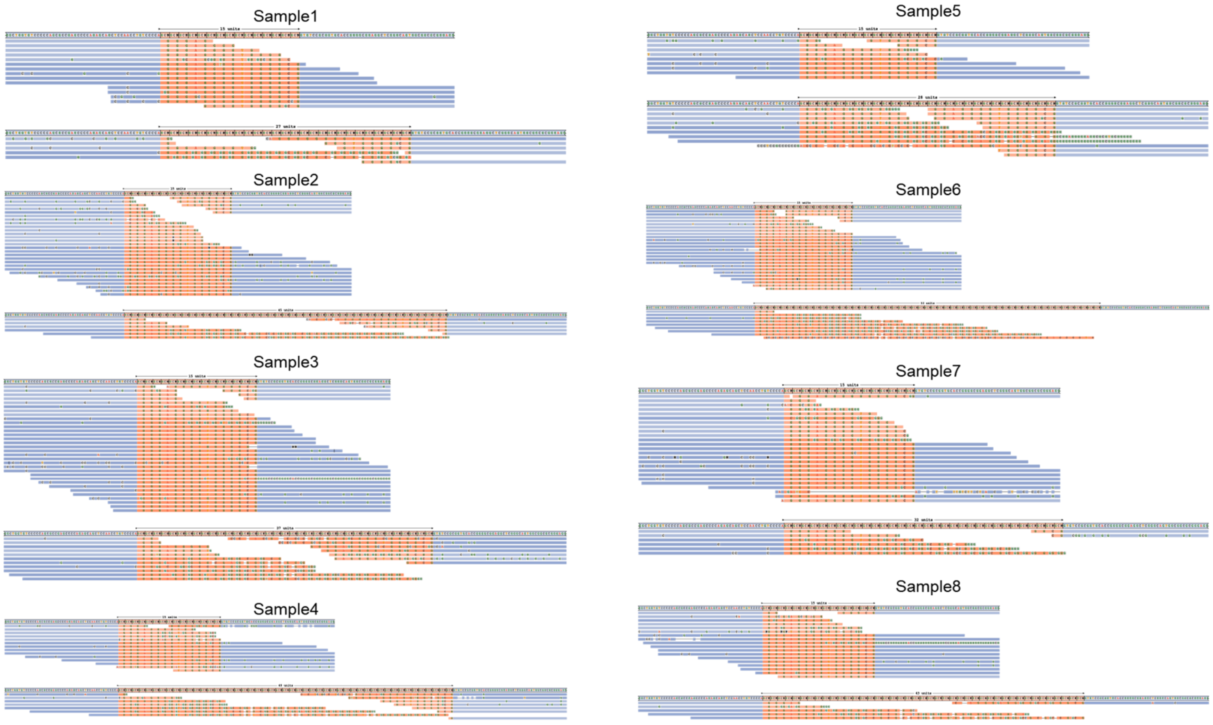

Fig. S33. Visualization of read alignment for expanded alleles of *ZIC2*:(GCN)n in eight Tibetan samples.

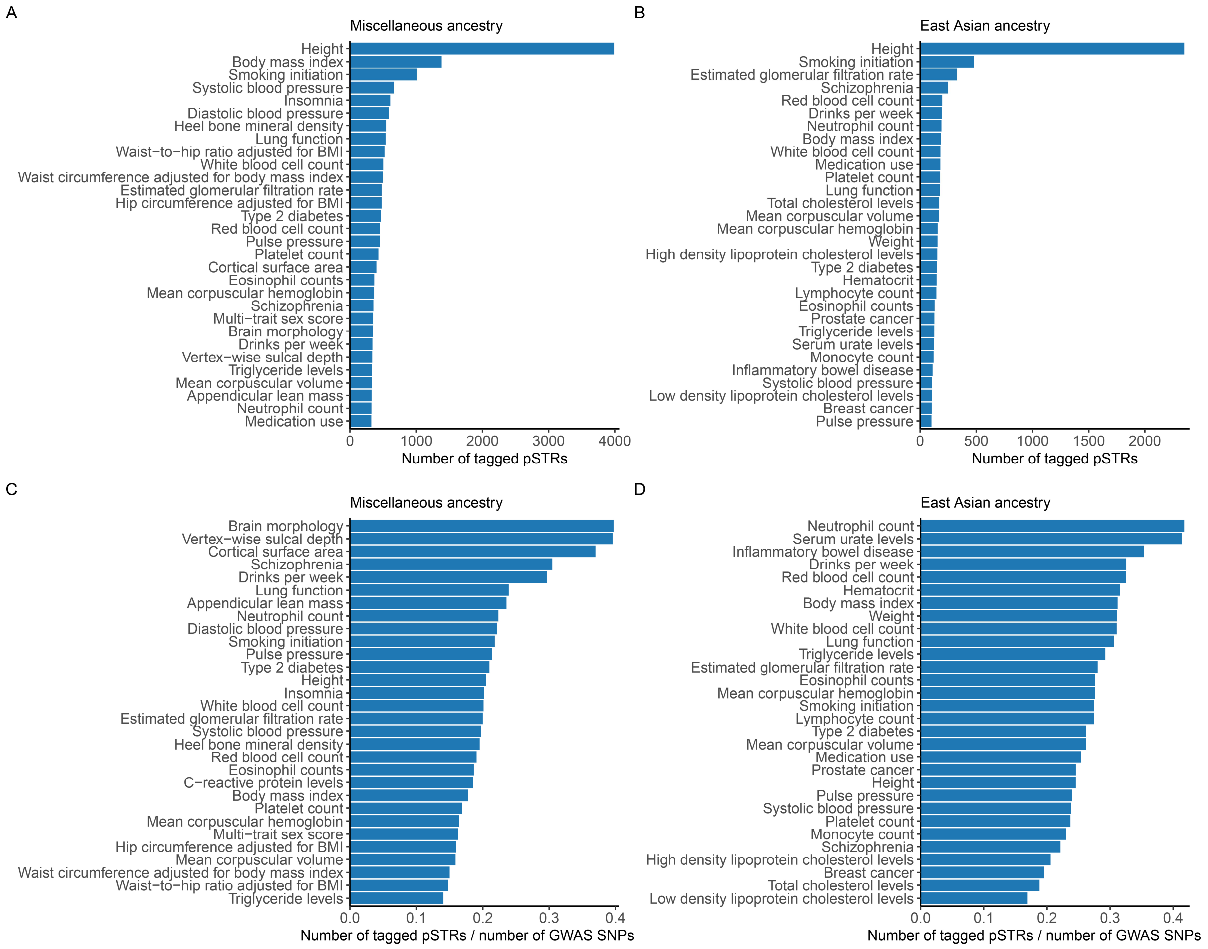

Fig. S34. Number of pSTRs in high linkage disequilibrium (Pearson's $r^2 > 0.8$) with GWAS risk SNPs associated with different human traits. (**A**) and (**B**) Raw count of tagged pSTRs. (**C**) and (**D**) Count of pSTRs adjusted for the number of GWAS risk SNPs tested for each trait. This analysis was separately performed on GWAS datasets from miscellaneous ancestry and East Asian ancestry. The top 30 traits with the highest number of tagged pSTRs are shown.

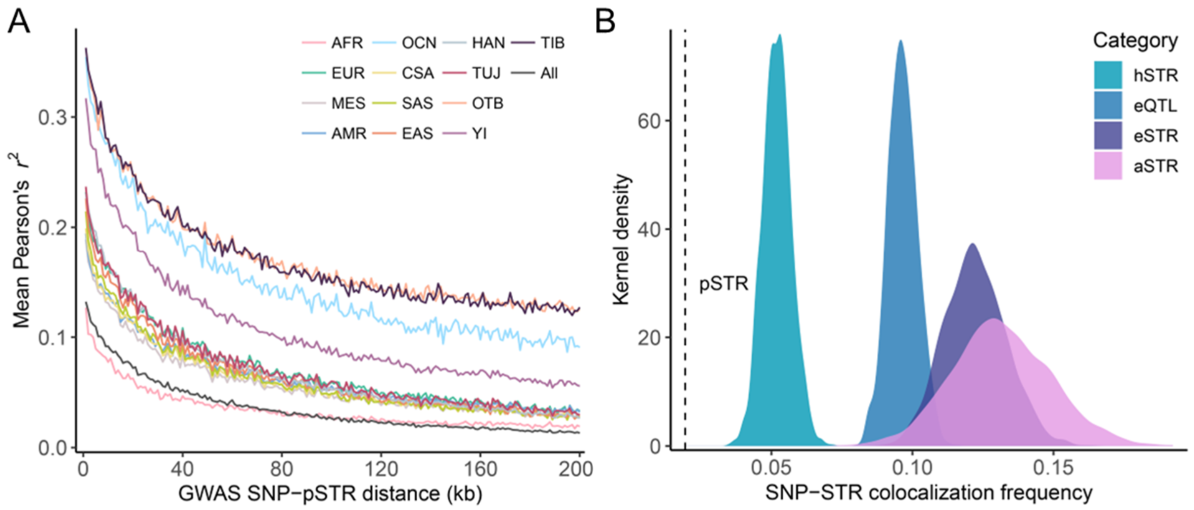

Fig. S35. Correlation of pSTRs with human health and disease traits in the GWAS catalog, focusing on studies involving participants of East Asian ancestry. (**A**) LD decay between pSTRs and GWAS risk SNPs across diverse populations. (**B**) Frequency of GWAS risk SNPs tagging for pSTRs in different functional or regulatory categories. The dashed line represents the mean tagging frequency for randomly permuted pSTRs.

Data S1. Information on samples analyzed in this study. This Excel file provides detailed information on the samples analyzed in this study that were sourced from the GSRD, e1kGP, and HGDP projects. The data include sample ID, sex, population, ancestry, language, sampling location, and whether the samples were used in different analyses. This file also provides the characteristics of the GSRD cohort.

Data S2. Population statistics of STR loci. This zipped plain text file includes allelic statistics for all pSTR and mSTR loci characterized in the integrated STR call set.

Data S3. pLoF variants annotated by VEP. This Excel file provides a summary of pLoF variants annotated by VEP and GO and KEGG enrichment results for affected LoF genes.

Data S4. Divergence of STRs between TB speakers and Han Chinese. This Excel file provides the Rst values and genomic contexts of divergent pSTRs between TB speakers (Tibetans, Tujia, and Yi people) and Han Chinese.

Data S5. Summary data for the eQTL analysis. This Excel file provides statistical summaries of the eQTL analysis.

Data S6. Summary data for STR dosage and environment association analysis. This Excel file provides statistical summaries of the STR dosage and environment association analysis.

Data S7. Information on KEGG pathways disturbed by adaptive STRs. This Excel file provides detailed information on KEGG pathways significantly disturbed by adaptive STRs.

Data S8. GWAS trait enrichment for risk genes tagged by eQTL STR loci. This Excel file provides a summary of GWAS trait enrichment analysis for GWAS risk genes tagged by eQTL loci.

Data S9. Fine-mapped STRs colocalized with GWAS signals. This Excel file provides information on fine-mapped eQTL loci that are colocalized with GWAS risk SNPs.

REFERENCES AND NOTES

1. S. Nurk, S. Koren, A. Rhie, M. Rautiainen, A. V. Bzikadze, A. Mikheenko, M. R. Vollger, N. Altemose, L. Uralsky, A. Gershman, S. Aganezov, S. J. Hoyt, M. Diekhans, G. A. Logsdon, M. Alonge, S. E. Antonarakis, M. Borchers, G. G. Bouffard, S. Y. Brooks, G. V. Caldas, N.-C. Chen, H. Cheng, C.-S. Chin, W. Chow, L. G. De Lima, P. C. Dishuck, R. Durbin, T. Dvorkina, I. T. Fiddes, G. Formenti, R. S. Fulton, A. Fungtammasan, E. Garrison, P. G. S. Grady, T. A. Graves-Lindsay, I. M. Hall, N. F. Hansen, G. A. Hartley, M. Haukness, K. Howe, M. W. Hunkapiller, C. Jain, M. Jain, E. D. Jarvis, P. Kerpedjiev, M. Kirsche, M. Kolmogorov, J. Korlach, M. Kremitzki, H. Li, V. V. Maduro, T. Marschall, A. M. McCartney, J. McDaniel, D. E. Miller, J. C. Mullikin, E. W. Myers, N. D. Olson, B. Paten, P. Peluso, P. A. Pevzner, D. Porubsky, T. Potapova, E. I. Rogaev, J. A. Rosenfeld, S. L. Salzberg, V. A. Schneider, F. J. Sedlazeck, K. Shafin, C. J. Shew, A. Shumate, Y. Sims, A. F. A. Smit, D. C. Soto, I. Sović, J. M. Storer, A. Streets, B. A. Sullivan, F. Thibaud-Nissen, J. Torrance, J. Wagner, B. P. Walenz, A. Wenger, J. M. D. Wood, C. Xiao, S. M. Yan, A. C. Young, S. Zarate, U. Surti, R. C. McCoy, M. Y. Dennis, I. A. Alexandrov, J. L. Gerton, R. J. O'Neill, W. Timp, J. M. Zook, M. C. Schatz, E. E. Eichler, K. H. Miga, A. M. Phillippy, The complete sequence of a human genome. *Science* **376**, 44–53 (2022).

2. M. Verbiest, M. Maksimov, Y. Jin, M. Anisimova, M. Gymrek, T. Bilgin Sonay, Mutation and selection processes regulating short tandem repeats give rise to genetic and phenotypic diversity across species. *J. Evol. Biol.* **36**, 321–336 (2023).

3. A. J. Hannan, Tandem repeats mediating genetic plasticity in health and disease. *Nat. Rev. Genet.* **19**, 286–298 (2018).

4. H. A. Tanudisastro, I. W. Deveson, H. Dashnow, D. G. MacArthur, Sequencing and characterizing short tandem repeats in the human genome. *Nat. Rev. Genet.* **25**, 460–475 (2024).

5. B. V. Halldorsson, H. P. Eggertsson, K. H. S. Moore, H. Hauswedell, O. Eiriksson, M. O. Ulfarsson, G. Palsson, M. T. Hardarson, A. Oddsson, B. O. Jensson, S. Kristmundsdottir, B. D. Sigurpalsdottir, O. A. Stefansson, D. Beyter, G. Holley, V. Tragante, A. Gylfason, P. I. Olason, F. Zink, M. Asgeirsdottir, S. T. Sverrisson, B. Sigurdsson, S. A. Gudjonsson, G. T. Sigurdsson, G. H. Halldorsson, G. Sveinbjornsson, K. Norland, U. Styrkarsdottir, D. N. Magnusdottir, S.

Snorradottir, K. Kristinsson, E. Sobech, H. Jonsson, A. J. Geirsson, I. Olafsson, P. Jonsson, O. B. Pedersen, C. Erikstrup, S. Brunak, S. R. Ostrowski, DBDS Genetic Consortium, S. Andersen, K. Banasik, K. Burgdorf, M. Didriksen, K. M. Dinh, C. Erikstrup, D. Gudbjartsson, T. F. Hansen, H. Hjalgrim, G. Jemec, P. Jennum, P. I. Johansson, M. A. H. Larsen, S. Mikkelsen, K. R. Nielsen, M. Nyegaard, S. R. Ostrowski, S. Sækmose, E. Sørensen, U. Thorsteinsdottir, M. T. Brun, H. Ullum, T. Werge, G. Thorleifsson, F. Jonsson, P. Melsted, I. Jonsdottir, T. Rafnar, H. Holm, H. Stefansson, J. Saemundsdottir, D. F. Gudbjartsson, O. T. Magnusson, G. Masson, U. Thorsteinsdottir, A. Helgason, H. Jonsson, P. Sulem, K. Stefansson, The sequences of 150,119 genomes in the UK Biobank. *Nature* **607**, 732–740 (2022).

6. J. Margoliash, S. Fuchs, Y. Li, X. Zhang, A. Massarat, A. Goren, M. Gymrek, Polymorphic short tandem repeats make widespread contributions to blood and serum traits. *Cell Genomics* **3**, 100458 (2023).

7. F. R. Wendt, G. A. Pathak, R. Polimanti, Phenome-wide association study of loci harboring de novo tandem repeat mutations in UK Biobank exomes. *Nat. Commun.* **13**, 7682 (2022).

8. C. A. Manigbas, B. Jadhav, P. Garg, M. Shadrina, W. Lee, G. Altman, A. Martin-Trujillo, A. J. Sharp, A phenome-wide association study of tandem repeat variation in 168,554 individuals from the UK Biobank. *Nat. Commun.* **15**, 10521 (2024).

9. B. I. Bustos, K. Billingsley, C. Blauwendraat, J. R. Gibbs, Z. Gan-Or, D. Krainc, A. B. Singleton, S. J. Lubbe, International Parkinson’s Disease Genomics Consortium (IPDGC), Genome-wide contribution of common short-tandem repeats to Parkinson’s disease genetic risk. *Brain* **146**, 65–74 (2023).

10. M. H. Guo, W.-P. Lee, B. Vardarajan, G. D. Schellenberg, J. E. Phillips-Cremins, Polygenic burden of short tandem repeat expansions promotes risk for Alzheimer’s disease. *Nat. Commun.* **16**, 1126 (2025).

11. I. Mitra, B. Huang, N. Mousavi, N. Ma, M. Lamkin, R. Yanicky, S. Shleizer-Burko, K. E. Lohmueller, M. Gymrek, Patterns of de novo tandem repeat mutations and their role in autism. *Nature* **589**, 246–250 (2021).

12. R. Gemayel, M. D. Vinces, M. Legendre, K. J. Verstrepen, Variable tandem repeats accelerate evolution of coding and regulatory sequences. *Annu. Rev. Genet.* **44**, 445–477 (2010).

13. O. K. Tørresen, B. Star, P. Mier, M. A. Andrade-Navarro, A. Bateman, P. Jarnot, A. Gruca, M. Grynberg, A. V. Kajava, V. J. Promponas, M. Anisimova, K. S. Jakobsen, D. Linke, Tandem repeats lead to sequence assembly errors and impose multi-level challenges for genome and protein databases. *Nucleic Acids Res.* **47**, 10994–11006 (2019).

14. A. H. Newton, A. J. Pask, Evolution and expansion of the RUNX2 QA repeat corresponds with the emergence of vertebrate complexity. *Commun. Biol.* **3**, 771 (2020).

15. W. B. Reinar, A. Greulich, I. M. Stø, J. B. Knutsen, T. Reitan, O. K. Tørresen, S. Jentoft, M. A. Butenko, K. S. Jakobsen, Adaptive protein evolution through length variation of short tandem repeats in *Arabidopsis*. *Sci. Adv.* **9**, eadd6960 (2023).

16. X. Qi, C. Cui, Y. Peng, X. Zhang, Z. Yang, H. Zhong, H. Zhang, K. Xiang, X. Cao, Y. Wang, Ouzhuluobu, Basang, Ciwangsangbu, Bianba, Gonggalanzi, T. Wu, H. Chen, H. Shi, B. Su, Genetic evidence of paleolithic colonization and neolithic expansion of modern humans on the Tibetan Plateau. *Mol. Biol. Evol.* **30**, 1761–1778 (2013).

17. X. L. Zhang, B. B. Ha, S. J. Wang, Z. J. Chen, J. Y. Ge, H. Long, W. He, W. Da, X. M. Nian, M. J. Yi, X. Y. Zhou, P. Q. Zhang, Y. S. Jin, O. Bar-Yosef, J. W. Olsen, X. Gao, The earliest human occupation of the high-altitude Tibetan Plateau 40 thousand to 30 thousand years ago. *Science* **362**, 1049–1051 (2018).

18. Y. He, W. Zheng, Y. Guo, T. Yue, C. Cui, H. Ouzhuluobu, K. Zhang, Z. Liu, T. Yang, J. Wu, Z.-B. Qu, J. Jin, F. Yang, X. Lu, B. S. Qi, Deep phenotyping of 11,880 highlanders reveals novel adaptive traits in native Tibetans. *iScience* **26**, 107677 (2023).

19. Y. He, Y. Guo, W. Zheng, T. Yue, H. Zhang, B. Wang, Z. Feng, C. Ouzhuluobu, K. Cui, B. Liu, X. Zhou, L. Zeng, T. Li, Y. Wang, C. Wang, S. Zhang, X. Xu, B. S. Qi, Polygenic adaptation leads to a higher reproductive fitness of native Tibetans at high altitude. *Curr. Biol.* **33**, 4037–4051.e5 (2023).

20. C. M. Beall, G. L. Cavalleri, L. Deng, R. C. Elston, Y. Gao, J. Knight, C. Li, J. C. Li, Y. Liang, M. McCormack, H. E. Montgomery, H. Pan, P. A. Robbins, K. V. Shianna, S. C. Tam, N. Tsering, K. R. Veeramah, W. Wang, P. Wangdui, M. E. Weale, Y. Xu, Z. Xu, L. Yang, M. J. Zaman, C. Zeng, L. Zhang, X. Zhang, P. Zhaxi, Y. T. Zheng, Natural selection on *EPAS1* (*HIF2*α) associated with low hemoglobin concentration in Tibetan highlanders. *Proc. Natl. Acad. Sci. U.S.A.* **107**, 11459–11464 (2010).

21. X. Yi, Y. Liang, E. Huerta-Sanchez, X. Jin, Z. X. P. Cuo, J. E. Pool, X. Xu, H. Jiang, N. Vinckenbosch, T. S. Korneliussen, H. Zheng, T. Liu, W. He, K. Li, R. Luo, X. Nie, H. Wu, M. Zhao, H. Cao, J. Zou, Y. Shan, S. Li, Q. Yang, Asan, P. Ni, G. Tian, J. Xu, X. Liu, T. Jiang, R. Wu, G. Zhou, M. Tang, J. Qin, T. Wang, S. Feng, G. Li, Huasang, J. Luosang, W. Wang, F. Chen, Y. Wang, X. Zheng, Z. Li, Z. Bianba, G. Yang, X. Wang, S. Tang, G. Gao, Y. Chen, Z. Luo, L. Gusang, Z. Cao, Q. Zhang, W. Ouyang, X. Ren, H. Liang, H. Zheng, Y. Huang, J. Li, L. Bolund, K. Kristiansen, Y. Li, Y. Zhang, X. Zhang, R. Li, S. Li, H. Yang, R. Nielsen, J. Wang, J. Wang, Sequencing of 50 human exomes reveals adaptation to high altitude. *Science* **329**, 75–78 (2010).

22. T. S. Simonson, Y. Yang, C. D. Huff, H. Yun, G. Qin, D. J. Witherspoon, Z. Bai, F. R. Lorenzo, J. Xing, L. B. Jorde, J. T. Prchal, R. Ge, Genetic evidence for high-altitude adaptation in Tibet. *Science* **329**, 72–75 (2010).

23. A. W. Bigham, F. S. Lee, Human high-altitude adaptation: Forward genetics meets the HIF pathway. *Genes Dev.* **28**, 2189–2204 (2014).

24. W. Zheng, Y. He, Y. Guo, T. Yue, H. Zhang, J. Li, B. Zhou, X. Zeng, L. Li, B. Wang, J. Cao, L. Chen, C. Li, H. Li, C. Cui, C. Bai, Baimakangzhuo, X. Qi, Ouzhuluobu, B. Su, Large-scale genome sequencing redefines the genetic footprints of high-altitude adaptation in Tibetans. *Genome Biol.* **24**, 73 (2023).

25. Ouzhuluobu, Y. He, H. Lou, C. Cui, L. Deng, Y. Gao, W. Zheng, Y. Guo, X. Wang, Z. Ning, J. Li, B. Li, C. Bai, Baimakangzhuo, Gonggalanzi, Dejiquzong, Bianba, Duojizhuoma, S. Liu, T. Wu, S. Xu, X. Qi, B. Su, De novo assembly of a Tibetan genome and identification of novel structural variants associated with high-altitude adaptation. *Natl. Sci. Rev.* **7**, 391–402 (2020).

26. C. Quan, Y. Li, X. Liu, Y. Wang, J. Ping, Y. Lu, G. Zhou, Characterization of structural variation in Tibetans reveals new evidence of high-altitude adaptation and introgression. *Genome Biol.* **22**, 159 (2021).

27. J. Shi, Z. Jia, J. Sun, X. Wang, X. Zhao, C. Zhao, F. Liang, X. Song, J. Guan, X. Jia, J. Yang, Q. Chen, K. Yu, Q. Jia, J. Wu, D. Wang, Y. Xiao, X. Xu, Y. Liu, S. Wu, Q. Zhong, J. Wu, S. Cui, X. Bo, Z. Wu, M. Park, M. Kellis, K. He, Structural variants involved in high-altitude adaptation detected using single-molecule long-read sequencing. *Nat. Commun.* **14**, 8282 (2023).

28. Z. Zhang, Y. Zhang, Y. Wang, Z. Zhao, M. Yang, L. Zhang, B. Zhou, B. Xu, H. Zhang, T. Chen, W. Dai, Y. Zhou, S. Shi, R. Nielsen, S. C. Li, S. Li, The Tibetan-Yi region is both a corridor and a barrier for human gene flow. *Cell Rep.* **39**, 110720 (2022).

29. M. Zhang, S. Yan, W. Pan, L. Jin, Phylogenetic evidence for Sino-Tibetan origin in northern China in the Late Neolithic. *Nature* **569**, 112–115 (2019).

30. C.-C. Wang, H.-Y. Yeh, A. N. Popov, H.-Q. Zhang, H. Matsumura, K. Sirak, O. Cheronet, A. Kovalev, N. Rohland, A. M. Kim, S. Mallick, R. Bernardos, D. Tumen, J. Zhao, Y.-C. Liu, J.-Y. Liu, M. Mah, K. Wang, Z. Zhang, N. Adamski, N. Broomandkhoshbacht, K. Callan, F. Candilio, K. S. D. Carlson, B. J. Culleton, L. Eccles, S. Freilich, D. Keating, A. M. Lawson, K. Mandl, M. Michel, J. Oppenheimer, K. T. Özdoğan, K. Stewardson, S. Wen, S. Yan, F. Zalzala, R. Chuang, C.-J. Huang, H. Looh, C.-C. Shiung, Y. G. Nikitin, A. V. Tabarev, A. A. Tishkin, S. Lin, Z.-Y. Sun, X.-M. Wu, T.-L. Yang, X. Hu, L. Chen, H. Du, J. Bayarsaikhan, E. Mijiddorj, D. Erdenebaatar, T.-O. Iderkhangai, E. Myagmar, H. Kanzawa-Kiriyama, M. Nishino, K. Shinoda, O. A. Shubina, J. Guo, W. Cai, Q. Deng, L. Kang, D. Li, D. Li, R. Lin, Nini, R. Shrestha, L.-X. Wang, L. Wei, G. Xie, H. Yao, M. Zhang, G. He, X. Yang, R. Hu, M. Robbeets, S. Schiffels, D. J. Kennett, L. Jin, H. Li, J. Krause, R. Pinhasi, D. Reich, Genomic insights into the formation of human populations in East Asia. *Nature* **591**, 413–419 (2021).

31. Y. Shi, Y. Niu, P. Zhang, H. Luo, S. Liu, S. Zhang, J. Wang, Y. Li, X. Liu, T. Song, T. Xu, S. He, Characterization of genome-wide STR variation in 6487 human genomes. *Nat. Commun.* **14**, 2092 (2023).

32. M. Byrska-Bishop, U. S. Evani, X. Zhao, A. O. Basile, H. J. Abel, A. A. Regier, A. Corvelo, W. E. Clarke, R. Musunuri, K. Nagulapalli, S. Fairley, A. Runnels, L. Winterkorn, E. Lowy, Human Genome Structural Variation Consortium, P. Flicek, S. Germer, H. Brand, I. M. Hall, M. E. Talkowski, G. Narzisi, M. C. Zody, High-coverage whole-genome sequencing of the expanded 1000 Genomes Project cohort including 602 trios. *Cell* **185**, 3426–3440.e19 (2022).

33. A. Bergström, S. A. McCarthy, R. Hui, M. A. Almarri, Q. Ayub, P. Danecek, Y. Chen, S. Felkel, P. Hallast, J. Kamm, H. Blanché, J.-F. Deleuze, H. Cann, S. Mallick, D. Reich, M. S. Sandhu, P. Skoglund, A. Scally, Y. Xue, R. Durbin, C. Tyler-Smith, Insights into human genetic variation and population history from 929 diverse genomes. *Science* **367**, eaay5012 (2020).

34. D. Lu, H. Lou, K. Yuan, X. Wang, Y. Wang, C. Zhang, Y. Lu, X. Yang, L. Deng, Y. Zhou, Q. Feng, Y. Hu, Q. Ding, Y. Yang, S. Li, L. Jin, Y. Guan, B. Su, L. Kang, S. Xu, Ancestral origins and genetic history of tibetan highlanders. *Am. J. Hum. Genet.* **99**, 580–594 (2016).

35. H. Z. Jam, Y. Li, R. DeVito, N. Mousavi, N. Ma, I. Lujumba, Y. Adam, M. Maksimov, B. Huang, E. Dolzhenko, Y. Qiu, F. E. Kakembo, H. Joseph, B. Onyido, J. Adeyemi, M. Bakhtiari, J. Park, S. Javadzadeh, D. Jjingo, E. Adebiyi, V. Bafna, M. Gymrek, A deep population reference panel of tandem repeat variation. *Nat. Commun.* **14**, 6711 (2023).

36. T. Willems, D. Zielinski, J. Yuan, A. Gordon, M. Gymrek, Y. Erlich, Genome-wide profiling of heritable and de novo STR variations. *Nat. Methods* **14**, 590–592 (2017).

37. N. Mousavi, S. Shleizer-Burko, R. Yanicky, M. Gymrek, Profiling the genome-wide landscape of tandem repeat expansions. *Nucleic Acids Res.* **47**, e90 (2019).

38. T. J. Pemberton, C. I. Sandefur, M. Jakobsson, N. A. Rosenberg, Sequence determinants of human microsatellite variability. *BMC Genomics* **10**, 612 (2009).

39. L. Ren, X. Duan, L. Dong, R. Zhang, J. Yang, Y. Gao, R. Peng, W. Hou, Y. Liu, J. Li, Y. Yu, N. Zhang, J. Shang, F. Liang, D. Wang, H. Chen, L. Sun, L. Hao, The Quartet Project Team, A. Scherer, J. Nordlund, W. Xiao, J. Xu, W. Tong, X. Hu, P. Jia, K. Ye, J. Li, L. Jin, H. Hong, J. Wang, S. Fan, X. Fang, Y. Zheng, L. Shi, Quartet DNA reference materials and datasets for

comprehensively evaluating germline variant calling performance. *Genome Biol.* **24**, 270 (2023).

40. K. J. Karczewski, L. C. Francioli, G. Tiao, B. B. Cummings, J. Alföldi, Q. Wang, R. L. Collins, K. M. Laricchia, A. Ganna, D. P. Birnbaum, L. D. Gauthier, H. Brand, M. Solomonson, N. A. Watts, D. Rhodes, M. Singer-Berk, E. M. England, E. G. Seaby, J. A. Kosmicki, R. K. Walters, K. Tashman, Y. Farjoun, E. Banks, T. Poterba, A. Wang, C. Seed, N. Whiffin, J. X. Chong, K. E. Samocha, E. Pierce-Hoffman, Z. Zappala, A. H. O'Donnell-Luria, E. V. Minikel, B. Weisburd, M. Lek, J. S. Ware, C. Vittal, I. M. Armean, L. Bergelson, K. Cibulskis, K. M. Connolly, M. Covarrubias, S. Donnelly, S. Ferriera, S. Gabriel, J. Gentry, N. Gupta, T. Jeandet, D. Kaplan, C. Llanwarne, R. Munshi, S. Novod, N. Petrillo, D. Roazen, V. Ruano-Rubio, A. Saltzman, M. Schleicher, J. Soto, K. Tibbetts, C. Tolonen, G. Wade, M. E. Talkowski, Genome Aggregation Database Consortium, C. A. Aguilar Salinas, T. Ahmad, C. M. Albert, D. Ardissino, G. Atzmon, J. Barnard, L. Beaugerie, E. J. Benjamin, M. Boehnke, L. L. Bonnycastle, E. P. Bottinger, D. W. Bowden, M. J. Bown, J. C. Chambers, J. C. Chan, D. Chasman, J. Cho, M. K. Chung, B. Cohen, A. Correa, D. Dabelea, M. J. Daly, D. Darbar, R. Duggirala, J. Dupuis, P. T. Ellinor, R. Elosua, J. Erdmann, T. Esko, M. Färkkilä, J. Florez, A. Franke, G. Getz, B. Glaser, S. J. Glatt, D. Goldstein, C. Gonzalez, L. Groop, C. Haiman, C. Hanis, M. Harms, M. Hiltunen, M. M. Holi, C. M. Hultman, M. Kallela, J. Kaprio, S. Kathiresan, B.-J. Kim, Y. J. Kim, G. Kirov, J. Kooner, S. Koskinen, H. M. Krumholz, S. Kugathasan, S. H. Kwak, M. Laakso, T. Lehtimäki, R. J. F. Loos, S. A. Lubitz, R. C. W. Ma, D. G. MacArthur, J. Marrugat, K. M. Mattila, S. McCarroll, M. I. McCarthy, D. McGovern, R. McPherson, J. B. Meigs, O. Melander, A. Metspalu, B. M. Neale, P. M. Nilsson, M. C. O'Donovan, D. Ongur, L. Orozco, M. J. Owen, C. N. A. Palmer, A. Palotie, K. S. Park, C. Pato, A. E. Pulver, N. Rahman, A. M. Remes, J. D. Rioux, S. Ripatti, D. M. Roden, D. Saleheen, V. Salomaa, N. J. Samani, J. Scharf, H. Schunkert, M. B. Shoemaker, P. Sklar, H. Soininen, H. Sokol, T. Spector, P. F. Sullivan, J. Suvisaari, E. S. Tai, Y. Y. Teo, T. Tiinamaija, M. Tsuang, D. Turner, T. Tusie-Luna, E. Vartiainen, M. P. Vawter, J. S. Ware, H. Watkins, R. K. Weersma, M. Wessman, J. G. Wilson, R. J. Xavier, B. M. Neale, M. J. Daly, D. G. MacArthur, The mutational constraint spectrum quantified from variation in 141,456 humans. *Nature* **581**, 434–443 (2020).

41. W. McLaren, L. Gil, S. E. Hunt, H. S. Riat, G. R. S. Ritchie, A. Thormann, P. Flicek, F. Cunningham, The Ensembl Variant Effect Predictor. *Genome Biol.* **17**, 122 (2016).

42. Exome Aggregation Consortium, M. Lek, K. J. Karczewski, E. V. Minikel, K. E. Samocha, E. Banks, T. Fennell, A. H. O'Donnell-Luria, J. S. Ware, A. J. Hill, B. B. Cummings, T. Tukiainen, D. P. Birnbaum, J. A. Kosmicki, L. E. Duncan, K. Estrada, F. Zhao, J. Zou, E. Pierce-Hoffman, J. Berghout, D. N. Cooper, N. Deflaux, M. DePristo, R. Do, J. Flannick, M. Fromer, L. Gauthier, J. Goldstein, N. Gupta, D. Howrigan, A. Kiezun, M. I. Kurki, A. L. Moonshine, P. Natarajan, L. Orozco, G. M. Peloso, R. Poplin, M. A. Rivas, V. Ruano-Rubio, S. A. Rose, D. M. Ruderfer, K. Shakir, P. D. Stenson, C. Stevens, B. P. Thomas, G. Tiao, M. T. Tusie-Luna, B. Weisburd, H.-H. Won, D. Yu, D. M. Altshuler, D. Ardissino, M. Boehnke, J. Danesh, S. Donnelly, R. Elosua, J. C. Florez, S. B. Gabriel, G. Getz, S. J. Glatt, C. M. Hultman, S. Kathiresan, M. Laakso, S. McCarroll, M. I. McCarthy, D. McGovern, R. McPherson, B. M. Neale, A. Palotie, S. M. Purcell, D. Saleheen, J. M. Scharf, P. Sklar, P. F. Sullivan, J. Tuomilehto, M. T. Tsuang, H. C. Watkins, J. G. Wilson, M. J. Daly, D. G. MacArthur, Analysis of protein-coding genetic variation in 60,706 humans. *Nature* **536**, 285–291 (2016).

43. R. L. Collins, J. T. Glessner, E. Porcu, M. Lepamets, R. Brandon, C. Lauricella, L. Han, T. Morley, L.-M. Niestroj, J. Ulirsch, S. Everett, D. P. Howrigan, P. M. Boone, J. Fu, K. J. Karczewski, G. Kellaris, C. Lowther, D. Lucente, K. Mohajeri, M. Nõukas, X. Nuttle, K. E. Samocha, M. Trinh, F. Ullah, U. Võsa, M. E. Hurles, S. Aradhya, E. E. Davis, H. Finucane, J. F. Gusella, A. Janze, N. Katsanis, L. Matyakhina, B. M. Neale, D. Sanders, S. Warren, J. C. Hodge, D. Lal, D. M. Ruderfer, J. Meck, R. Mägi, T. Esko, A. Reymond, Z. Kutalik, H. Hakonarson, S. Sunyaev, H. Brand, M. E. Talkowski, A. Metspalu, R. Mägi, M. Nelis, L. Milani, T. Esko, A cross-disorder dosage sensitivity map of the human genome. *Cell* **185**, 3041–3055.e25 (2022).

44. A. Bigham, M. Bauchet, D. Pinto, X. Mao, J. M. Akey, R. Mei, S. W. Scherer, C. G. Julian, M. J. Wilson, D. López Herráez, T. Brutsaert, E. J. Parra, L. G. Moore, M. D. Shriver, Identifying signatures of natural selection in Tibetan and Andean populations using dense genome scan data. *PLOS Genet.* **6**, e1001116 (2010).

45. C. Zhang, Y. Lu, Q. Feng, X. Wang, H. Lou, J. Liu, Z. Ning, K. Yuan, Y. Wang, Y. Zhou, L. Deng, L. Liu, Y. Yang, S. Li, L. Ma, Z. Zhang, L. Jin, B. Su, L. Kang, S. Xu, Differentiated demographic histories and local adaptations between Sherpas and Tibetans. *Genome Biol.* **18**, 115 (2017).

46. J. D. Bernstock, L. Peruzzotti-Jametti, T. Leonardi, N. Vicario, D. Ye, Y. Lee, D. Maric, K. R. Johnson, Y. Mou, A. Van Den Bosch, M. Winterbone, G. K. Friedman, R. J. M. Franklin, J. M. Hallenbeck, S. Pluchino, SUMOylation promotes survival and integration of neural stem cell grafts in ischemic stroke. *EBioMedicine* **42**, 214–224 (2019).

47. The ENCODE Project Consortium, F. Abascal, R. Acosta, N. J. Addleman, J. Adrian, V. Afzal, R. Ai, B. Aken, J. A. Akiyama, O. A. Jammal, H. Amrhein, S. M. Anderson, G. R. Andrews, I. Antoshechkin, K. G. Ardlie, J. Armstrong, M. Astley, B. Banerjee, A. A. Barkal, I. H. A. Barnes, I. Barozzi, D. Barrell, G. Barson, D. Bates, U. K. Baymuradov, C. Bazile, M. A. Beer, S. Beik, M. A. Bender, R. Bennett, L. P. B. Bouvrette, B. E. Bernstein, A. Berry, A. Bhaskar, A. Bignell, S. M. Blue, D. M. Bodine, C. Boix, N. Boley, T. Borrman, B. Borsari, A. P. Boyle, L. A. Brandsmeier, A. Breschi, E. H. Bresnick, J. A. Brooks, M. Buckley, C. B. Burge, R. Byron, E. Cahill, L. Cai, L. Cao, M. Carty, R. G. Castanon, A. Castillo, H. Chaib, E. T. Chan, D. R. Chee, S. Chee, H. Chen, H. Chen, J.-Y. Chen, S. Chen, J. M. Cherry, S. B. Chhetri, J. S. Choudhary, J. Chrast, D. Chung, D. Clarke, N. A. L. Cody, C. J. Coppola, J. Coursen, A. M. D'Ippolito, S. Dalton, C. Danyko, C. Davidson, J. Davila-Velderrain, C. A. Davis, J. Dekker, A. Deran, G. DeSalvo, G. Despacio-Reyes, C. N. Dewey, D. E. Dickel, M. Diegel, M. Diekhans, V. Dileep, B. Ding, S. Djebali, A. Dobin, D. Dominguez, S. Donaldson, J. Drenkow, T. R. Dreszer, Y. Drier, M. O. Duff, D. Dunn, C. Eastman, J. R. Ecker, M. D. Edwards, N. El-Ali, S. I. Elhajjajy, K. Elkins, *A. Emili*, C. B. Epstein, R. C. Evans, I. Ezkurdia, K. Fan, P. J. Farnham, N. P. Farrell, E. A. Feingold, A.-M. Ferreira, K. Fisher-Aylor, S. Fitzgerald, P. Flicek, C. S. Foo, K. Fortier, A. Frankish, P. Freese, S. Fu, X.-D. Fu, Y. Fu, Y. Fukuda-Yuzawa, M. Fulciniti, A. P. W. Funnell, I. Gabdank, T. Galeev, M. Gao, C. G. Giron, T. H. Garvin, C. A. Gelboin-Burkhart, G. Georgolopoulos, M. B. Gerstein, B. M. Giardine, D. K. Gifford, D. M. Gilbert, D. A. Gilchrist, S. Gillespie, T. R. Gingeras, P. Gong, A. Gonzalez, J. M. Gonzalez, P. Good, A. Goren, D. U. Gorkin, B. R. Graveley, M. Gray, J. F. Greenblatt, E. Griffiths, M. T. Groudine, F. Grubert, M. Gu, R. Guigó, H. Guo, Y. Guo, Y. Guo, G. Gursoy, M. Gutierrez-Arcelus, J. Halow, R. C. Hardison, M. Hardy, M. Hariharan,

A. Harmanci, A. Harrington, J. L. Harrow, T. B. Hashimoto, R. D. Hasz, M. Hatan, E. Haugen, J. E. Hayes, P. He, Y. He, N. Heidari, D. Hendrickson, E. F. Heuston, J. A. Hilton, B. C. Hitz, A. Hochman, C. Holgren, L. Hou, S. Hou, Y.-H. E. Hsiao, S. Hsu, H. Huang, T. J. Hubbard, J. Huey, T. R. Hughes, T. Hunt, S. Ibarrientos, R. Issner, M. Iwata, O. Izuogu, T. Jaakkola, N. Jameel, C. Jansen, L. Jiang, P. Jiang, A. Johnson, R. Johnson, I. Jungreis, M. Kadaba, M. Kasowski, M. Kasparian, M. Kato, R. Kaul, T. Kawli, M. Kay, J. C. Keen, S. Keles, C. A. Keller, D. Kelley, M. Kellis, P. Kheradpour, D. S. Kim, A. Kirilusha, R. J. Klein, B. Knoechel, S. Kuan, M. J. Kulik, S. Kumar, A. Kundaje, T. Kutyavin, J. Lagarde, B. R. Lajoie, N. J. Lambert, J. Lazar, A. Y. Lee, D. Lee, E. Lee, J. W. Lee, K. Lee, C. S. Leslie, S. Levy, B. Li, H. Li, N. Li, S. Li, X. Li, Y. I. Li, Y. Li, Y. Li, Y. Li, J. Lian, M. W. Libbrecht, S. Lin, Y. Lin, D. Liu, J. Liu, P. Liu, T. Liu, X. S. Liu, Y. Liu, Y. Liu, M. Long, S. Lou, J. Loveland, A. Lu, Y. Lu, E. Lécuyer, L. Ma, M. Mackiewicz, B. J. Mannion, M. Mannstadt, D. Manthravadi, G. K. Marinov, F. J. Martin, E. Mattei, K. McCue, M. McEown, G. McVicker, S. K. Meadows, A. Meissner, E. M. Mendenhall, C. L. Messer, W. Meuleman, C. Meyer, S. Miller, M. G. Milton, T. Mishra, D. E. Moore, H. M. Moore, J. E. Moore, S. H. Moore, J. Moran, A. Mortazavi, J. M. Mudge, N. Munshi, R. Murad, R. M. Myers, V. Nandakumar, P. Nandi, A. M. Narasimha, A. K. Narayanan, H. Naughton, F. C. P. Navarro, P. Navas, J. Nazarovs, J. Nelson, S. Neph, F. J. Neri, J. R. Nery, A. R. Nesmith, J. S. Newberry, K. M. Newberry, V. Ngo, R. Nguyen, T. B. Nguyen, T. Nguyen, A. Nishida, W. S. Noble, C. S. Novak, E. M. Novoa, B. Nuñez, C. W. O’Donnell, S. Olson, K. C. Onate, E. Otterman, H. Ozadam, M. Pagan, T. Palden, X. Pan, Y. Park, E. C. Partridge, B. Paten, F. Pauli-Behn, M. J. Pazin, B. Pei, L. A. Pennacchio, A. R. Perez, E. H. Perry, D. D. Pervouchine, N. N. Phalke, Q. Pham, D. H. Phanstiel, I. Plajzer-Frick, G. A. Pratt, H. E. Pratt, S. Preissl, J. K. Pritchard, Y. Pritykin, M. J. Purcaro, Q. Qin, G. Quinones-Valdez, I. Rabano, E. Radovani, A. Raj, N. Rajagopal, O. Ram, L. Ramirez, R. N. Ramirez, D. Rausch, S. Raychaudhuri, J. Raymond, R. Razavi, T. E. Reddy, T. M. Reimonn, B. Ren, A. Reymond, A. Reynolds, S. K. Rhie, J. Rinn, M. Rivera, J. C. Rivera-Mulia, B. S. Roberts, J. M. Rodriguez, J. Rozowsky, R. Ryan, E. Rynes, D. N. Salins, R. Sandstrom, T. Sasaki, S. Sathe, D. Savic, A. Scavelli, J. Scheiman, C. Schlaffner, J. A. Schloss, F. W. Schmitges, L. H. See, A. Sethi, M. Setty, A. Shafer, S. Shan, E. Sharon, Q. Shen, Y. Shen, R. I. Sherwood, M. Shi, S. Shin, N. Shoresh, K. Siebenthall, C. Sisu, T. Slifer, C. A. Sloan, A. Smith, V. Snetkova, M. P. Snyder, D. V. Spacek, S. Srinivasan, R. Srivas, G. Stamatoyannopoulos, J. A. Stamatoyannopoulos, R. Stanton, D. Steffan, S.

Stehling-Sun, J. S. Strattan, A. Su, B. Sundararaman, M.-M. Suner, T. Syed, M. Szynkarek, F. Y. Tanaka, D. Tenen, M. Teng, J. A. Thomas, D. Toffey, M. L. Tress, D. E. Trout, G. Trynka, J. Tsuji, S. A. Upchurch, O. Ursu, B. Uszczynska-Ratajczak, M. C. Uziel, A. Valencia, B. V. Biber, A. G. Van Der Velde, E. L. Van Nostrand, Y. Vaydylevich, J. Vazquez, A. Victorsen, J. Vielmetter, J. Vierstra, A. Visel, A. Vlasova, C. M. Vockley, S. Volpi, S. Vong, H. Wang, M. Wang, Q. Wang, R. Wang, T. Wang, W. Wang, X. Wang, Y. Wang, N. K. Watson, X. Wei, Z. Wei, H. Weisser, S. M. Weissman, R. Welch, R. E. Welikson, Z. Weng, H.-J. Westra, J. W. Whitaker, C. White, K. P. White, A. Wildberg, B. A. Williams, D. Wine, H. N. Witt, B. Wold, M. Wolf, J. Wright, R. Xiao, X. Xiao, J. Xu, J. Xu, K.-K. Yan, Y. Yan, H. Yang, X. Yang, Y.-W. Yang, G. G. Yardımcı, B. A. Yee, G. W. Yeo, T. Young, T. Yu, F. Yue, C. Zaleski, C. Zang, H. Zeng, W. Zeng, D. R. Zerbino, J. Zhai, L. Zhan, Y. Zhan, B. Zhang, J. Zhang, J. Zhang, K. Zhang, L. Zhang, P. Zhang, Q. Zhang, X.-O. Zhang, Y. Zhang, Z. Zhang, Y. Zhao, Y. Zheng, G. Zhong, X.-Q. Zhou, Y. Zhu, J. Zimmerman, J. E. Moore, M. J. Purcaro, H. E. Pratt, C. B. Epstein, N. Shoresh, J. Adrian, T. Kawli, C. A. Davis, A. Dobin, R. Kaul, J. Halow, E. L. Van Nostrand, P. Freese, D. U. Gorkin, Y. Shen, Y. He, M. Mackiewicz, F. Pauli-Behn, B. A. Williams, A. Mortazavi, C. A. Keller, X.-O. Zhang, S. I. Elhajjajy, J. Huey, D. E. Dickel, V. Snetkova, X. Wei, X. Wang, J. C. Rivera-Mulia, J. Rozowsky, J. Zhang, S. B. Chhetri, J. Zhang, A. Victorsen, K. P. White, A. Visel, G. W. Yeo, C. B. Burge, E. Lécuyer, D. M. Gilbert, J. Dekker, J. Rinn, E. M. Mendenhall, J. R. Ecker, M. Kellis, R. J. Klein, W. S. Noble, A. Kundaje, R. Guigó, P. J. Farnham, J. M. Cherry, R. M. Myers, B. Ren, B. R. Graveley, M. B. Gerstein, L. A. Pennacchio, M. P. Snyder, B. E. Bernstein, B. Wold, R. C. Hardison, T. R. Gingeras, J. A. Stamatoyannopoulos, Z. Weng, Expanded encyclopaedias of DNA elements in the human and mouse genomes. *Nature* **583**, 699–710 (2020).

48. J. Li, G. Zhang, D. Yin, Y. Li, Y. Zhang, J. Cheng, K. Zhang, J. Ji, T. Wang, Y. Jia, S. Yin, Integrated application of multiomics strategies provides insights into the environmental hypoxia response in *Pelteobagrus vachelli* muscle. *Mol. Cell. Proteomics* **21**, 100196 (2022).

49. Q. Gao, P. Ni, Y. Wang, P. Huo, X. Zhang, S. Wang, F. Xiao, Y. Li, W. Feng, J. Yuan, T. Zhang, Q. Li, B. Fan, Y. Kan, Z. Li, Y. Qi, J. Xing, Z. Yang, H. Cheng, X. Gao, X. Feng, M. Xue, Y. Liu, Y. Luo, Z. Lu, Y. Zhao, DDAH1 promotes neurogenesis and neural repair in cerebral ischemia. *Acta Pharma. Sin. B* **14**, 2097–2118 (2024).

50. Y. Zhang, G. Jin, J. Zhang, R. Mi, Y. Zhou, W. Fan, S. Cheng, W. Song, B. Zhang, M. Ma, F. Liu, Overexpression of STAT1 suppresses angiogenesis under hypoxia by regulating VEGF-A in human glioma cells. *Biomed. Pharmacother.* **104**, 566–575 (2018).

51. Roadmap Epigenomics Consortium, A. Kundaje, W. Meuleman, J. Ernst, M. Bilenky, A. Yen, A. Heravi-Moussavi, P. Kheradpour, Z. Zhang, J. Wang, M. J. Ziller, V. Amin, J. W. Whitaker, M. D. Schultz, L. D. Ward, A. Sarkar, G. Quon, R. S. Sandstrom, M. L. Eaton, Y.-C. Wu, A. R. Pfenning, X. Wang, M. Claussnitzer, Y. Liu, C. Coarfa, R. A. Harris, N. Shoresh, C. B. Epstein, E. Gjoneska, D. Leung, W. Xie, R. D. Hawkins, R. Lister, C. Hong, P. Gascard, A. J. Mungall, R. Moore, E. Chuah, A. Tam, T. K. Canfield, R. S. Hansen, R. Kaul, P. J. Sabo, M. S. Bansal, A. Carles, J. R. Dixon, K.-H. Farh, S. Feizi, R. Karlic, A.-R. Kim, A. Kulkarni, D. Li, R. Lowdon, G. Elliott, T. R. Mercer, S. J. Neph, V. Onuchic, P. Polak, N. Rajagopal, P. Ray, R. C. Sallari, K. T. Siebenthall, N. A. Sinnott-Armstrong, M. Stevens, R. E. Thurman, J. Wu, B. Zhang, X. Zhou, A. E. Beaudet, L. A. Boyer, P. L. De Jager, P. J. Farnham, S. J. Fisher, D. Haussler, S. J. M. Jones, W. Li, M. A. Marra, M. T. McManus, S. Sunyaev, J. A. Thomson, T. D. Tlsty, L.-H. Tsai, W. Wang, R. A. Waterland, M. Q. Zhang, L. H. Chadwick, B. E. Bernstein, J. F. Costello, J. R. Ecker, M. Hirst, A. Meissner, A. Milosavljevic, B. Ren, J. A. Stamatoyannopoulos, T. Wang, M. Kellis, Integrative analysis of 111 reference human epigenomes. *Nature* **518**, 317–330 (2015).

52. D. J. Taylor, S. B. Chhetri, M. G. Tassia, A. Biddanda, S. M. Yan, G. L. Wojcik, A. Battle, R. C. McCoy, Sources of gene expression variation in a globally diverse human cohort. *Nature* **632**, 122–130 (2024).

53. A. Siepel, G. Bejerano, J. S. Pedersen, A. S. Hinrichs, M. Hou, K. Rosenbloom, H. Clawson, J. Spieth, L. W. Hillier, S. Richards, G. M. Weinstock, R. K. Wilson, R. A. Gibbs, W. J. Kent, W. Miller, D. Haussler, Evolutionarily conserved elements in vertebrate, insect, worm, and yeast genomes. *Genome Res.* **15**, 1034–1050 (2005).

54. S. Brönneke, B. Brückner, J. Söhle, R. Siegner, C. Smuda, F. Stäb, H. Wenck, L. Kolbe, E. Grönniger, M. Winnefeld, Genome-wide expression analysis of wounded skin reveals novel genes involved in angiogenesis. *Angiogenesis* **18**, 361–371 (2015).

55. J. Abramson, J. Adler, J. Dunger, R. Evans, T. Green, A. Pritzel, O. Ronneberger, L. Willmore, A. J. Ballard, J. Bambrick, S. W. Bodenstein, D. A. Evans, C.-C. Hung, M. O'Neill, D. Reiman, K. Tunyasuvunakool, Z. Wu, A. Žemgulytė, E. Arvaniti, C. Beattie, O. Bertolli, A. Bridgland, A. Cherepanov, M. Congreve, A. I. Cowen-Rivers, A. Cowie, M. Figurnov, F. B. Fuchs, H. Gladman, R. Jain, Y. A. Khan, C. M. R. Low, K. Perlin, A. Potapenko, P. Savy, S. Singh, A. Stecula, A. Thillaisundaram, C. Tong, S. Yakneen, E. D. Zhong, M. Zielinski, A. Žídek, V. Bapst, P. Kohli, M. Jaderberg, D. Hassabis, J. M. Jumper, Accurate structure prediction of biomolecular interactions with AlphaFold 3. *Nature* **630**, 493–500 (2024).

56. W. Zheng, Q. Wuyun, Y. Li, Q. Liu, X. Zhou, C. Peng, Y. Zhu, L. Freddolino, Y. Zhang, Deep-learning-based single-domain and multidomain protein structure prediction with D-I-TASSER. *Nat. Biotechnol.*, 10.1038/s41587-025-02654-4 (2025).

57. J. Yin, J. Lv, S. Yang, Y. Wang, Z. Huang, X. Wang, G. Hou, W. Zhou, Y. Liu, W. Wang, X. Lin, Y. Huang, Y. Zheng, C. Wei, Y. Yuan, Y. Huang, C. Liu, H. Tao, H. Liu, R. Liu, Y. Zhang, G. Zeng, F. Quan, X. Zhu, P. Gao, J. Xie, L. Liu, J. Cao, C. Liu, X. Jin, J. Wang, Multi-omics reveals immune response and metabolic profiles during high-altitude mountaineering. *Cell Rep.* **44**, 115134 (2025).

58. M. Guo, M. Zhang, X. Cao, X. Fang, K. Li, L. Qin, Y. He, J. Zhao, Y. Xu, X. Liu, X. Li, Notch4 mediates vascular remodeling via ERK/JNK/P38 MAPK signaling pathways in hypoxic pulmonary hypertension. *Respir. Res.* **23**, 6 (2022).

59. K. M. Chen, A. K. Wong, O. G. Troyanskaya, J. Zhou, A sequence-based global map of regulatory activity for deciphering human genetics. *Nat. Genet.* **54**, 940–949 (2022).

60. T. Hao, J. Yu, Z. Wu, J. Jiang, L. Gong, B. Wang, H. Guo, H. Zhao, B. Lu, S. Engelender, H. He, Z. Song, Hypoxia-reprogramed megamitochondrion contacts and engulfs lysosome to mediate mitochondrial self-digestion. *Nat. Commun.* **14**, 4105 (2023).

61. D. C. Borradale, M. G. Kimlin, Folate degradation due to ultraviolet radiation: Possible implications for human health and nutrition. *Nutr. Rev.* **70**, 414–422 (2012).

62. J. Song, C. Jin, Z. Shan, W. Teng, J. Li, Prevalence and risk factors of hyperuricemia and gout: A cross-sectional survey from 31 provinces in mainland China. *J. Trans. Int. Med.* **10**, 134–145 (2022).

63. A. Jain, G. Tuteja, TissueEnrich: Tissue-specific gene enrichment analysis. *Bioinformatics* **35**, 1966–1967 (2019).

64. S. Danielli, Z. Ma, E. Pantazi, A. Kumar, B. Demarco, F. A. Fischer, U. Paudel, J. Weissenrieder, R. J. Lee, S. Joyce, J. K. Foskett, J. S. Bezbradica, The ion channel CALHM6 controls bacterial infection-induced cellular cross-talk at the immunological synapse. *EMBO J.* **42**, e111450 (2023).

65. S. Okagawa, Hepatic SerpinA1 improves energy and glucose metabolism through regulation of preadipocyte proliferation and UCP1 expression. *Nat. Commun.* **15**, 9585 (2024).

66. A. Sulovari, R. Li, P. A. Audano, D. Porubsky, M. R. Vollger, G. A. Logsdon, Human Genome Structural Variation Consortium, W. C. Warren, A. A. Pollen, M. J. P. Chaisson, E. E. Eichler, M. J. P. Chaisson, A. D. Sanders, X. Zhao, A. Malhotra, D. Porubsky, T. Rausch, E. J. Gardner, O. L. Rodriguez, L. Guo, R. L. Collins, X. Fan, J. Wen, R. E. Handsaker, S. Fairley, Z. N. Kronenberg, X. Kong, F. Hormozdiari, D. Lee, A. M. Wenger, A. R. Hastie, D. Antaki, T. Anantharaman, P. A. Audano, H. Brand, S. Cantsilieris, H. Cao, E. Cerveira, C. Chen, X. Chen, C.-S. Chin, Z. Chong, N. T. Chuang, C. C. Lambert, D. M. Church, L. Clarke, A. Farrell, J. Flores, T. Galeey, D. U. Gorkin, M. Gujral, V. Guryev, W. H. Heaton, J. Korlach, S. Kumar, J. Y. Kwon, E. T. Lam, J. E. Lee, J. Lee, W.-P. Lee, S. P. Lee, S. Li, P. Marks, K. Viaud-Martinez, S. Meiers, K. M. Munson, F. C. P. Navarro, B. J. Nelson, C. Nodzak, A. Noor, S. Kyriazopoulou-Panagiotopoulou, A. W. C. Pang, Y. Qiu, G. Rosanio, M. Ryan, A. Stütz, D. C. J. Spierings, A. Ward, A. E. Welch, M. Xiao, W. Xu, C. Zhang, Q. Zhu, X. Zheng-Bradley, E. Lowy, S. Yakneen, S. McCarroll, G. Jun, L. Ding, C. L. Koh, B. Ren, P. Flicek, K. Chen, M. B. Gerstein, P.-Y. Kwok, P. M. Lansdorp, G. T. Marth, J. Sebat, X. Shi, A. Bashir, K. Ye, S. E. Devine, M. E. Talkowski, R. E. Mills, T. Marschall, J. O. Korbel, E. E. Eichler, C. Lee, Human-specific tandem repeat expansion and differential gene expression during primate evolution. *Proc. Natl. Acad. Sci. U.S.A.* **116**, 23243–23253 (2019).

67. E. Dolzhenko, V. Deshpande, F. Schlesinger, P. Krusche, R. Petrovski, S. Chen, D. Emig-Agius, A. Gross, G. Narzisi, B. Bowman, K. Scheffler, J. J. F. A. Van Vugt, C. French, A. Sanchis-Juan, K. Ibáñez, A. Tucci, B. R. Lajoie, J. H. Veldink, F. L. Raymond, R. J. Taft, D. R. Bentley, M. A. Eberle, ExpansionHunter: A sequence-graph-based tool to analyze variation in short tandem repeat regions. *Bioinformatics* **35**, 4754–4756 (2019).

68. J. J. Adashek, C. Pandya, N. J. Maragakis, P. De, P. R. Cohen, S. Kato, R. Kurzrock, Neuregulin-1 and ALS19 (ERBB4): At the crossroads of amyotrophic lateral sclerosis and cancer. *BMC Med.* **22**, 74 (2024).

69. X. Wang, Y. Zhou, L. Ning, J. Chen, H. Chen, X. Li, Knockdown of ANXA10 induces ferroptosis by inhibiting autophagy-mediated TFRC degradation in colorectal cancer. *Cell Death Dis.* **14**, 588 (2023).

70. M. Wagner, J. Lévy, S. Jung-Klawitter, S. Bakhtiari, F. Monteiro, R. Maroofian, T. Bierhals, M. Hempel, M. Elmaleh-Bergès, J. P. Kitajima, C. A. Kim, J. G. Salomao, D. J. Amor, M. S. Cooper, L. Perrin, E. Pipiras, A. Neu, M. Doosti, E. G. Karimiani, M. B. Toosi, H. Houlden, S. C. Jin, Y. C. Si, L. H. Rodan, H. Venselaar, M. C. Kruer, F. Kok, G. F. Hoffmann, T. M. Strom, S. B. Wortmann, A.-C. Tabet, T. Opladen, Loss of TNR causes a nonprogressive neurodevelopmental disorder with spasticity and transient opisthotonus. *Genet. Med.* **22**, 1061–1068 (2020).

71. T. Almeida, I. Alonso, S. Martins, E. M. Ramos, L. Azevedo, K. Ohno, Ancestral origin of the ATTCT repeat expansion in spinocerebellar ataxia type 10 (SCA10). *PLOS ONE* **4**, e4553 (2009).

72. K. Ibañez, B. Jadhav, M. Zanovello, D. Gagliardi, C. Clarkson, S. Facchini, P. Garg, A. Martin-Trujillo, S. J. Gies, V. Galassi Deforie, A. Dalmia, D. J. Hensman Moss, J. Vandrovcova, C. Rocca, L. Moutsianas, C. Marini-Bettolo, H. Walker, C. Turner, M. Shoai, J. D. Long, P. Fratta, D. R. Langbehn, S. J. Tabrizi, M. J. Caulfield, A. Cortese, V. Escott-Price, J. Hardy, H. Houlden, A. J. Sharp, A. Tucci, Increased frequency of repeat expansion mutations across different populations. *Nat. Med.* **30**, 3357–3368 (2024).

73. L. Y. Brown, S. Odent, V. David, M. Blayau, C. Dubourg, C. Apacik, M. A. Delgado, B. D. Hall, J. F. Reynolds, A. Sommer, D. Wieczorek, S. A. Brown, M. Muenke, Holoprosencephaly due to mutations in ZIC2: Alanine tract expansion mutations may be caused by parental somatic recombination. *Hum. Mol. Genet.* **10**, 791–796 (2001).

74. A. Buniello, J. A. L. MacArthur, M. Cerezo, L. W. Harris, J. Hayhurst, C. Malangone, A. McMahon, J. Morales, E. Mountjoy, E. Sollis, D. Suveges, O. Vrousgou, P. L. Whetzel, R. Amode, J. A. Guillen, H. S. Riat, S. J. Trevanion, P. Hall, H. Junkins, P. Flicek, T. Burdett, L. A. Hindorff, F. Cunningham, H. Parkinson, The NHGRI-EBI GWAS catalog of published genome-wide association studies, targeted arrays and summary statistics 2019. *Nucleic Acids Res.* **47**, D1005–D1012 (2019).

75. N. Shrine, A. G. Izquierdo, J. Chen, R. Packer, R. J. Hall, A. L. Guyatt, C. Batini, R. J. Thompson, C. Pavuluri, V. Malik, B. D. Hobbs, M. Moll, W. Kim, R. Tal-Singer, P. Bakke, K. A. Fawcett, C. John, K. Coley, N. N. Piga, A. Pozarickij, K. Lin, I. Y. Millwood, Z. Chen, L. Li, China Kadoorie Biobank Collaborative Group, S. R. A. Wijnant, L. Lahousse, G. Brusselle, A. G. Uitterlinden, A. Manichaikul, E. C. Oelsner, S. S. Rich, R. G. Barr, S. M. Kerr, V. Vitart, M. R. Brown, M. Wielscher, M. Imboden, A. Jeong, T. M. Bartz, S. A. Gharib, C. Flexeder, S. Karrasch, C. Gieger, A. Peters, B. Stubbe, X. Hu, V. E. Ortega, D. A. Meyers, E. R. Bleecker, S. B. Gabriel, N. Gupta, A. V. Smith, J. Luan, J.-H. Zhao, A. F. Hansen, A. Langhammer, C. Willer, L. Bhatta, D. Porteous, B. H. Smith, A. Campbell, T. Sofer, J. Lee, M. L. Daviglus, B. Yu, E. Lim, H. Xu, G. T. O'Connor, G. Thareja, O. M. E. Albagha, Qatar Genome Program Research (QGPR) Consortium, K. Suhre, R. Granell, T. O. Faquih, P. S. Hiemstra, A. M. Slats, B. H. Mullin, J. Hui, A. James, J. Beilby, K. Patasova, P. Hysi, J. T. Koskela, A. B. Wyss, J. Jin, S. Sikdar, M. Lee, S. May-Wilson, N. Pirastu, K. A. Kentistou, P. K. Joshi, P. R. H. J. Timmers, A. T. Williams, R. C. Free, X. Wang, J. L. Morrison, F. D. Gilliland, Z. Chen, C. A. Wang, R. E. Foong, S. E. Harris, A. Taylor, P. Redmond, J. P. Cook, A. Mahajan, L. Lind, T. Palviainen, T. Lehtimäki, O. T. Raitakari, J. Kaprio, T. Rantanen, K. H. Pietiläinen, S. R. Cox, C. E. Pennell, G. L. Hall, W. J. Gauderman, C. Brightling, J. F. Wilson, T. Vasankari, T. Laitinen, V. Salomaa, D. O. Mook-Kanamori, N. J. Timpson, E. Zeggini, J. Dupuis, C. Hayward, B. Brumpton, C. Langenberg, S. Weiss, G. Homuth, C. O. Schmidt, N. Probst-Hensch, M.-R. Jarvelin, A. C. Morrison, O. Polasek, I. Rudan, J.-H. Lee, I. Sayers, E. L. Rawlins, F. Dudbridge, E. K. Silverman, D. P. Strachan, R. G. Walters, A. P.

Morris, S. J. London, M. H. Cho, L. V. Wain, I. P. Hall, M. D. Tobin, Multi-ancestry genome-wide association analyses improve resolution of genes and pathways influencing lung function and chronic obstructive pulmonary disease risk. *Nat. Genet.* **55**, 410–422 (2023).

76. S. Koyama, K. Ito, C. Terao, M. Akiyama, M. Horikoshi, Y. Momozawa, H. Matsunaga, H. Ieki, K. Ozaki, Y. Onouchi, A. Takahashi, S. Nomura, H. Morita, H. Akazawa, C. Kim, J. Seo, K. Higasa, M. Iwasaki, T. Yamaji, N. Sawada, S. Tsugane, T. Koyama, H. Ikezaki, N. Takashima, K. Tanaka, K. Arisawa, K. Kuriki, M. Naito, K. Wakai, S. Suna, Y. Sakata, H. Sato, M. Hori, Y. Sakata, K. Matsuda, Y. Murakami, H. Aburatani, M. Kubo, F. Matsuda, Y. Kamatani, I. Komuro, Population-specific and trans-ancestry genome-wide analyses identify distinct and shared genetic risk loci for coronary artery disease. *Nat. Genet.* **52**, 1169–1177 (2020).

77. Y. Cui, W. Ye, J. S. Li, J. J. Li, E. Vilain, T. Sallam, W. Li, A genome-wide spectrum of tandem repeat expansions in 338,963 humans. *Cell* **187**, 2336–2341.e5 (2024).

78. R. Albalat, C. Cañestro, Evolution by gene loss. *Nat. Rev. Genet.* **17**, 379–391 (2016).

79. Y.-C. Xu, Y.-L. Guo, Less is more, natural loss-of-function mutation is a strategy for adaptation. *Plant Commun.* **1**, 100103 (2020).

80. S. F. Fotsing, J. Margoliash, C. Wang, S. Saini, R. Yanicky, S. Shleizer-Burko, A. Goren, M. Gymrek, The impact of short tandem repeat variation on gene expression. *Nat. Genet.* **51**, 1652–1659 (2019).

81. C. A. Horton, A. M. Alexandari, M. G. B. Hayes, E. Marklund, J. M. Schaepe, A. K. Aditham, N. Shah, P. H. Suzuki, A. Shrikumar, A. Afek, W. J. Greenleaf, R. Gordân, J. Zeitlinger, A. Kundaje, P. M. Fordyce, Short tandem repeats bind transcription factors to tune eukaryotic gene expression. *Science* **381**, eadd1250 (2023).

82. S. Hussain, N. Sadouni, D. van Essen, L. T. M. Dao, Q. Ferré, G. Charbonnier, M. Torres, F. Gallardo, C.-H. Lecellier, T. Sexton, S. Saccani, S. Spicuglia, Short tandem repeats are important contributors to silencer elements in T cells. *Nucleic Acids Res.* **51**, 4845–4866 (2023).

83. I.-S. Rajan-Babu, J. J. Peng, R. Chiu, IMAGINE Study, P. Birch, M. Couse, C. Guimond, A. Lehman, J. Mwenifumbo, C. Van Karnebeek, J. Friedman, CAUSES Study, S. Adam, C. D. Souich, A. Elliott, A. Lehman, J. Mwenifumbo, T. Nelson, C. Van Karnebeek, J. Friedman, C. Li, A. Mohajeri, E. Dolzhenko, M. A. Eberle, I. Birol, J. M. Friedman, Genome-wide sequencing as a first-tier screening test for short tandem repeat expansions. *Genome Med.* **13**, 126 (2021).

84. A. C. English, E. Dolzhenko, H. Ziaei Jam, S. K. McKenzie, N. D. Olson, W. De Coster, J. Park, B. Gu, J. Wagner, M. A. Eberle, M. Gymrek, M. J. P. Chaisson, J. M. Zook, F. J. Sedlazeck, Analysis and benchmarking of small and large genomic variants across tandem repeats. *Nat. Biotechnol.* **43**, 431–442 (2025).

85. T.-Y. Lu, The Human Genome Structural Variation Consortium, K. M. Munson, A. P. Lewis, Q. Zhu, L. J. Tallon, S. E. Devine, C. Lee, E. E. Eichler, M. J. P. Chaisson, Profiling variable-number tandem repeat variation across populations using repeat-pangenome graphs. *Nat. Commun.* **12**, 4250 (2021).

86. E. Dolzhenko, A. English, H. Dashnow, G. De Sena Brandine, T. Mokveld, W. J. Rowell, C. Karniski, Z. Kronenberg, M. C. Danzi, W. A. Cheung, C. Bi, E. Farrow, A. Wenger, K. P. Chua, V. Martínez-Cerdeño, T. D. Bartley, P. Jin, D. L. Nelson, S. Zuchner, T. Pastinen, A. R. Quinlan, F. J. Sedlazeck, M. A. Eberle, Characterization and visualization of tandem repeats at genome scale. *Nat. Biotechnol.* **42**, 1606–1614 (2024).

87. Y. Cui, Multi-omic quantitative trait loci link tandem repeat size variation to gene regulation in human brain. *Nat. Genet.* **57**, 369–378 (2025).

88. J. E. Rood, S. Wynne, L. Robson, A. Hupalowska, J. Randell, S. A. Teichmann, A. Regev, The human cell atlas from a cell census to a unified foundation model. *Nature* **637**, 1065–1071 (2025).

89. S. Chen, Y. Zhou, Y. Chen, J. Gu, fastp: An ultra-fast all-in-one FASTQ preprocessor. *Bioinformatics* **34**, i884–i890 (2018).

90. H. Li, Aligning sequence reads, clone sequences and assembly contigs with BWA-MEM. arXiv:1303.3997 [q-bio.GN] (2013).

91. P. Danecek, J. K. Bonfield, J. Liddle, J. Marshall, V. Ohan, M. O. Pollard, A. Whitwham, T. Keane, S. A. McCarthy, R. M. Davies, H. Li, Twelve years of SAMtools and BCFtools. *GigaScience* **10**, giab008 (2021).

92. A. R. Quinlan, I. M. Hall, BEDTools: A flexible suite of utilities for comparing genomic features. *Bioinformatics* **26**, 841–842 (2010).

93. N. Mousavi, J. Margoliash, N. Pusarla, S. Saini, R. Yanicky, M. Gymrek, TRTools: A toolkit for genome-wide analysis of tandem repeats. *Bioinformatics* **37**, 731–733 (2021).

94. P. Danecek, A. Auton, G. Abecasis, C. A. Albers, E. Banks, M. A. DePristo, R. E. Handsaker, G. Lunter, G. T. Marth, S. T. Sherry, G. McVean, R. Durbin, 1000 Genomes Project Analysis Group, The variant call format and VCFtools. *Bioinformatics* **27**, 2156–2158 (2011).

95. M. A. DePristo, E. Banks, R. Poplin, K. V. Garimella, J. R. Maguire, C. Hartl, A. A. Philippakis, G. Del Angel, M. A. Rivas, M. Hanna, A. McKenna, T. J. Fennell, A. M. Kernytsky, A. Y. Sivachenko, K. Cibulskis, S. B. Gabriel, D. Altshuler, M. J. Daly, A framework for variation discovery and genotyping using next-generation DNA sequencing data. *Nat. Genet.* **43**, 491–498 (2011).

96. S. Purcell, B. Neale, K. Todd-Brown, L. Thomas, M. A. R. Ferreira, D. Bender, J. Maller, P. Sklar, P. I. W. de Bakker, M. J. Daly, P. C. Sham, PLINK: A tool set for whole-genome association and population-based linkage analyses. *Am. J. Hum. Genet.* **81**, 559–575 (2007).

97. J. Staples, D. A. Nickerson, J. E. Below, Utilizing graph theory to select the largest set of unrelated individuals for genetic analysis. *Genet. Epidemiol.* **37**, 136–141 (2013).

98. T. Wu, E. Hu, S. Xu, M. Chen, P. Guo, Z. Dai, T. Feng, L. Zhou, W. Tang, L. Zhan, X. Fu, S. Liu, X. Bo, G. Yu, clusterProfiler 4.0: A universal enrichment tool for interpreting omics data. *The*. *Innovation* **2**, 100141 (2021).

99. Z. Li, J. Meisner, A. Albrechtsen, Fast and accurate out-of-core PCA framework for large scale biobank data. *Genome Res.* **33**, 1599–1608 (2023).

100. L. McInnes, J. Healy, J. Melville, UMAP: Uniform Manifold Approximation and Projection for dimension reduction. arXiv:1802.03426 [stat.ML] (2018).

101. A. B. Conley, L. Rishishwar, M. Ahmad, S. Sharma, E. T. Norris, I. K. Jordan, L. Mariño-Ramírez, Rye: Genetic ancestry inference at biobank scale. *Nucleic Acids Res.* **51**, e44 (2023).

102. D. H. Alexander, J. Novembre, K. Lange, Fast model-based estimation of ancestry in unrelated individuals. *Genome Res.* **19**, 1655–1664 (2009).

103. M. Slatkin, A measure of population subdivision based on microsatellite allele frequencies. *Genetics* **139**, 457–462 (1995).

104. R. Schefzik, J. Flesch, A. Goncalves, Fast identification of differential distributions in single-cell RNA-sequencing data with waddR. *Bioinformatics* **37**, 3204–3211 (2021).

105. A. Heger, C. Webber, M. Goodson, C. P. Ponting, G. Lunter, GAT: A simulation framework for testing the association of genomic intervals. *Bioinformatics* **29**, 2046–2048 (2013).

106. O. Stegle, L. Parts, R. Durbin, J. Winn, A bayesian framework to account for complex non-genetic factors in gene expression levels greatly increases power in eQTL studies. *PLoS Comput. Biol.* **6**, e1000770 (2010).

107. R Core Team, *R: A Language and Environment for Statistical Computing (R Foundation for Statistical Computing* (2024); www.R-project.org/.

108. S. E. Fick, R. J. Hijmans, WorldClim 2: New 1-km spatial resolution climate surfaces for global land areas. *Int. J. Climatol.* **37**, 4302–4315 (2017).

109. S. M. Urbut, G. Wang, P. Carbonetto, M. Stephens, Flexible statistical methods for estimating and testing effects in genomic studies with multiple conditions. *Nat. Genet.* **51**, 187–195 (2019).

110. A. L. M. Reis, M. Rapadas, J. M. Hammond, H. Gamaarachchi, I. Stevanovski, M. A. Kumaheri, S. R. Chintalaphani, D. S. B. Dissanayake, O. M. Siggs, A. W. Hewitt, B. Llamas, A. Brown, G. Baynam, G. J. Mann, B. J. McMorran, S. Easteal, A. Hermes, M. R. Jenkins, The National Centre for Indigenous Genomics, G. Pearson, Y. Roe, J. Mohamed, B. Murray, L. Ormond-Parker, E. Kneipp, K. Nugent, G. Mann, H. R. Patel, I. W. Deveson, The landscape of genomic structural variation in Indigenous Australians. *Nature* **624**, 602–610 (2023).

111. M. O. Press, R. C. McCoy, A. N. Hall, J. M. Akey, C. Queitsch, Massive variation of short tandem repeats with functional consequences across strains of *Arabidopsis thaliana*. *Genome Res.* **28**, 1169–1178 (2018).

112. Z. Gu, R. Eils, M. Schlesner, Complex heatmaps reveal patterns and correlations in multidimensional genomic data. *Bioinformatics* **32**, 2847–2849 (2016).

113. A. Halman, E. Dolzhenko, A. Oshlack, STRipy: A graphical application for enhanced genotyping of pathogenic short tandem repeats in sequencing data. *Hum. Mutat.* **43**, 859–868 (2022).

114. E. Dolzhenko, B. Weisburd, K. Ibañez, I.-S. Rajan-Babu, C. Anyansi, M. F. Bennett, K. Billingsley, A. Carroll, S. Clamons, M. C. Danzi, V. Deshpande, J. Ding, S. Fazal, A. Halman, B. Jadhav, Y. Qiu, P. A. Richmond, C. T. Saunders, K. Scheffler, J. J. F. A. van Vugt, R. R. A. J. Zwamborn, S. S. Chong, J. M. Friedman, A. Tucci, H. L. Rehm, M. A. Eberle, Genomics England Research Consortium, REViewer: Haplotype-resolved visualization of read alignments in and around tandem repeats. *Genome Med.* **14**, 84 (2022).

115. Y. Zou, P. Carbonetto, G. Wang, M. Stephens, Fine-mapping from summary data with the “sum of single effects” model. *PLOS Genet.* **18**, e1010299 (2022).

116. Y. Huang, M. Wang, C. Liu, G. He, Comprehensive landscape of non-CODIS STRs in global populations provides new insights into challenging DNA profiles. *Forensic Sci. Int. Genet.* **70**, 103010 (2024).

117. G. Benson, Tandem repeats finder: A program to analyze DNA sequences. *Nucleic Acids Res.* **27**, 573–580 (1999).
